# Integrated analysis of lncRNA and mRNA transcriptomes reveals the potential regulatory role of lncRNA in kiwifruit ripening and softening

**DOI:** 10.1038/s41598-021-81155-1

**Published:** 2021-01-18

**Authors:** Yiting Chen, Chunzhen Cheng, Xin Feng, Ruilian Lai, Minxia Gao, Wenguang Chen, Rujian Wu

**Affiliations:** 1 Fruit Research Institute, Fujian Academy of Agricultural Sciences, Fuzhou, 350013 Fujian China; 2 Research Centre for Engineering Technology of Fujian Deciduous Fruits, Fuzhou, 350013 Fujian China; 3 College of Horticulture, Fujian Agriculture and Forestry University, Fuzhou, 350002 Fujian China

**Keywords:** Plant sciences, Plant molecular biology

## Abstract

Kiwifruit has gained increasing attention worldwide for its unique flavor and high nutritional value. Rapid softening after harvest greatly shortens its shelf-life and reduces the commercial value. Therefore, it is imperative and urgent to identify and clarify its softening mechanism. This study aimed to analyze and compare the long noncoding RNA (lncRNA) and mRNA expression patterns in ABA-treated (ABA) and room temperature (RT)-stored fruits with those in freshly harvested fruits (CK) as control. A total of 697 differentially expressed genes (DEGs) and 81 differentially expressed lncRNAs (DELs) were identified while comparing ABA with CK, and 458 DEGs and 143 DELs were detected while comparing RT with CK. The Kyoto Encyclopedia of Genes and Genomes analysis of the identified DEGs and the target genes of DELs revealed that genes involved in starch and sucrose metabolism, brassinosteroid biosynthesis, plant hormone signal transduction, and flavonoid biosynthesis accounted for a large part. The co-localization networks, including 38 DEGs and 31 DELs in ABA vs. CK, and 25 DEGs and 25 DELs in RT vs. CK, were also performed. Genes related to fruit ripening, such as genes encoding β-galactosidase, mannan endo-1,4-β-mannosidase, pectinesterase/pectinesterase inhibitor, and NAC transcription factor, were present in the co-localization network, suggesting that lncRNAs were involved in regulating kiwifruit ripening. Notably, several ethylene biosynthesis- and signaling-related genes, including one 1-aminocyclopropane-1-carboxylic acid oxidase gene and three ethylene response factor genes, were found in the co-localization network of ABA vs. CK, suggesting that the promoting effect of ABA on ethylene biosynthesis and fruit softening might be embodied by increasing the expression of these lncRNAs. These results may help understand the regulatory mechanism of lncRNAs in ripening and ABA-induced fruit softening of kiwifruit.

## Introduction

Kiwifruit has gained increasing attention for its unique flavor and high vitamin C content. However, rapid softening during the ripening process reduces its commercial value. Fruits are generally categorized as climacteric and nonclimacteric based on their respiratory rates and associated ethylene biosynthesis profiles during ripening^1^. Kiwifruit has been classified as a climacteric fruit, but its ripening process differs a lot from typical climacteric fruits, such as tomato and banana; the initiation of softening, ethylene production, and climacteric during kiwifruit ripening are temporally separate. The ripening of kiwifruit can be categorized into three distinct phases: slow softening Phase I, rapid softening Phase II, and ethylene-dependent Phase III^2^. In Phase I, the ripening process is initiated but the firmness of kiwifruit reduces slowly. In Phase II, the fruit firmness rapidly reduces to ~ 20% of the harvest value. In Phase III, the production of internal ethylene starts, and fruits undergo respiratory climacteric. Healthy intact kiwifruit can soften without exogenous ethylene treatment. The first two ripening phases of kiwifruit occur in the apparent absence of ethylene production and prior to the respiratory climacteric, revealing that the early ripening phases of kiwifruit are ethylene independent, and some other regulators may exist during these phases^3^. The pivotal role of abscisic acid (ABA) in regulating fruit ripening and softening has been identified in many climacteric fruits^4–6^. Exogenous ABA treatment can induce the expression of genes encoding 1-aminocyclopropane-1-carboxylic acid synthase (ACS) and oxidase (ACO), consequently promoting ethylene synthesis and fruit ripening^7^. The role of ABA in kiwifruit ripening and softening is found to be more important than that of ethylene, and therefore ABA is considered as a key type of fruit-ripening hormone^8^.

The distinctive ripening and softening process of kiwifruit has gained immense interests. Postharvest ripening indices for kiwifruit mainly include starch degradation, cell wall metabolism, ethylene biosynthesis, and aroma generation^3^. Numerous studies have been performed to clarify the ripening mechanism of kiwifruit from different aspects, including chemical and histochemical analyses, enzyme activity changes, and gene and protein expression levels^9–11^. Kiwifruit is one of the high starch-containing fruits, which can degrade starch into soluble sugar after harvest^3^. Multiple genes encoding enzymes involved in starch and sucrose metabolism during kiwifruit ripening have been isolated, such as genes encoding α-amylase and β-amylase^12^. The changes of cell wall composition is related to the softening of kiwifruit. A number of structural genes related to cell wall metabolism have been characterized in kiwifruit, including genes encoding polygalacturonase, xyloglucan endotransglycosylase/hydrolase, pectinesterase, and expansin^11,13,14^. Ethylene production in the later stage accelerates the softening and decay of kiwifruit. ACS and ACO are two key enzymes in the biosynthesis pathway of ethylene^15^. The RNAi transgene of *ACO* gene in kiwifruit significantly inhibited ethylene production and fruit softening^3^. Ethylene signaling components, such as ethylene receptor genes and ethylene response factor genes, were also demonstrated to participate in kiwifruit ripening^16,17^. However, the exact regulatory mechanism remains unclear. One reason may be that kiwifruit ripening is a complex process regulated by various factors, such as transcription factors and noncoding RNAs, at the transcriptional and post-transcriptional levels. Next-generation sequencing is a powerful tool for analyzing gene expression changes during certain processes or under certain treatments, which is widely used to elucidate the mechanism of fruit ripening and softening^18,19^.

Long noncoding RNAs (lncRNAs) are a class of transcripts comprising more than 200 nucleotides, which lack protein-coding ability. LncRNAs can be classified into three types according to their genomic positions and orientations relative to adjacent protein-coding genes: long intergenic lncRNA (lincRNA), intronic lncRNA, and antisense lncRNA^20^. LncRNAs were reported to play important roles in various biological processes, including chromatin remodeling, gene transcription, post-transcriptional regulation, and protein modification^20,21^. With the rapid development of sequencing technology, many lncRNAs have been identified from various plants, such as *Arabidopsis thaliana*^22^, *Brassica rapa*^23^, *Oryza sativa*^24^, and *Solanum lycopersicum*^25^. For example, 152, 233, and 197 differentially expressed lncRNAs (DELs) in three ovule developmental stages were identified in rice^26^. LncRNAs are not only abundant in plants but also involved in plant growth, development, and stress responses. An lncRNA, transcribed from the antisense strand of its neighboring gene (leucine-rich repeat receptor kinase, LRK), was overexpressed in rice, which increased rice grain yield and upregulated the expression of several LRK genes^27^. In tomato, ethylene production and lycopene accumulation were largely repressed when lncRNA1459 was knocked out^28^, while lncRNA16397 induced glutaredoxin expression to reduce reactive oxygen species accumulation, conferring resistance to *Phytophthora infestans*^29^. However, the knowledge of plant lncRNAs is limited due to the diversity and low sequence conservation. Therefore, studies on lncRNAs of additional plant species with representative characteristics are required.

Given the importance of ABA in fruit softening and the limited knowledge of lncRNAs involved in kiwifruit ripening, genome-wide expression analysis of genes and lncRNAs was necessary. The whole-genome sequences of *Actinidia chinensis* were made available to the public^30^, facilitating the exploration and identification of lncRNAs. In this study, comparative transcriptome analysis was performed to identify differentially expressed genes (DEGs) and lncRNAs in ABA-treated and room temperature-stored fruits compared with freshly harvested fruits. The integrated analysis of lncRNA and mRNA expression profiles might help understand the ripening mechanism of kiwifruit and provide clues for further elucidation of the role of ABA and lncRNAs in fruit ripening and softening.

## Results

### Physiological properties of kiwifruit during ripening and softening

**Figure 1:**
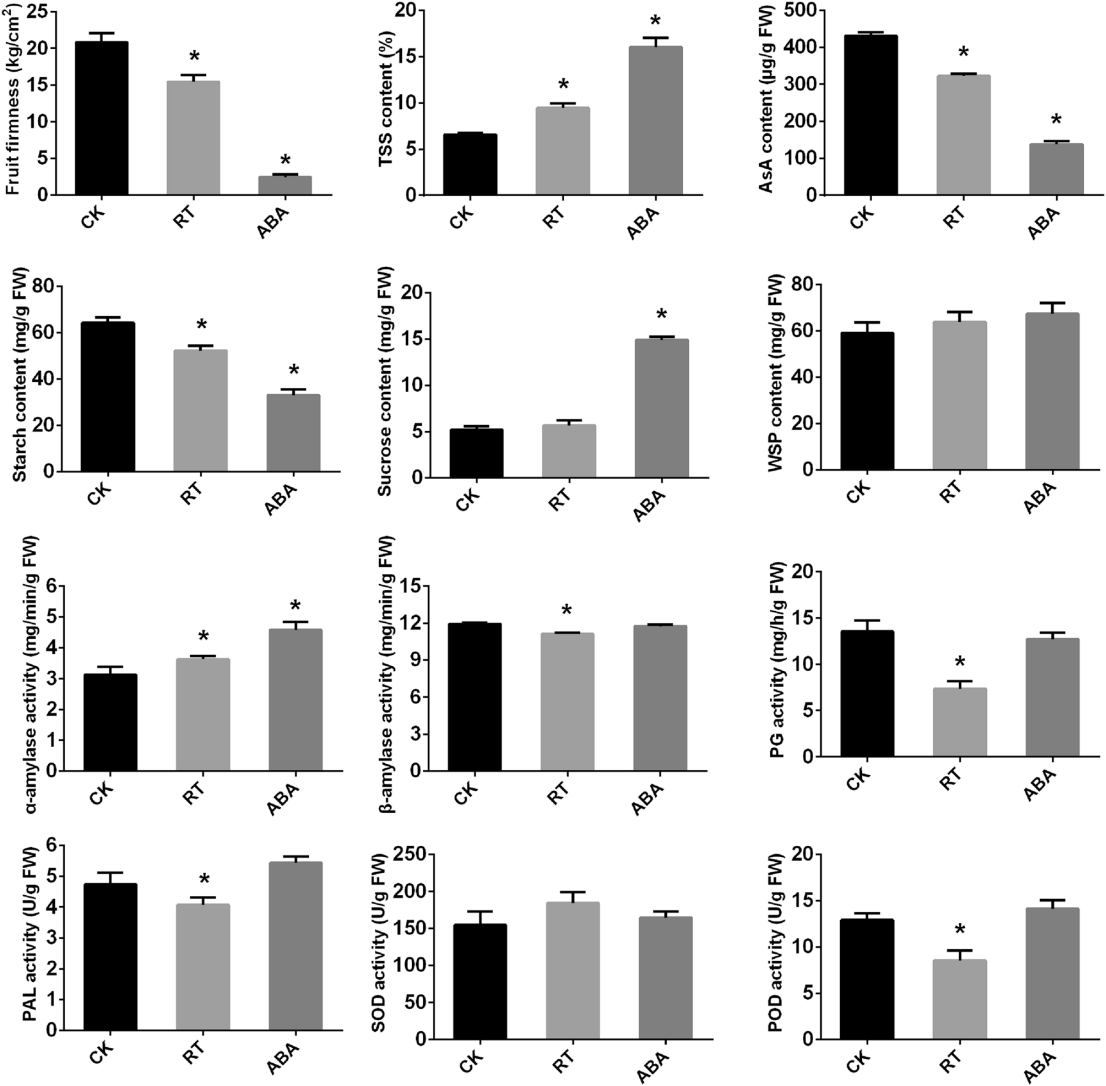
Physiological changes in kiwifruits under different treatments. AsA, ascorbic acid; FW, fresh weight; PAL, phenylalnine ammonialyase; PG, polygalacturonase; POD, peroxidase; SOD, superoxide dismutase; TSS, total soluble solid; WPS, water soluble pectin. CK, freshly harvested fruits (fruit maturity stage); RT, fruits stored at 25 °C for 7 days (postharvest ripening stage); ABA, fruits treated with 50 mg/L ABA for 2 min and then stored at 25 °C for 7 days (softening stage). One-way analysis of variance (ANOVA) in the SPSS software was used for statistical analysis. The asterisk indicates significant differences (*P* < 0.05).

Fruit inclusions and enzyme activities changed during kiwifruit ripening and softening (Fig. 1). In most fruits, firmness gradually decreased with postharvest ripening. The fruit firmness of kiwifruit decreased from 20.84 kg/cm^2^ in the CK group to 15.47 kg/cm^2^ in the RT group, while in the ABA group, it declined rapidly to 2.47 kg/cm^2^. Starch degradation is generally considered to be the first sign of kiwifruit postharvest ripening, which also contributes to the increase in total soluble solid (TSS). The changes in starch and ascorbic acid (AsA) contents were like those in fruit firmness. The starch content decreased from 64.26 mg/g in the CK group to 52.32 mg/g in the RT group, and to 32.93 mg/g in the ABA group. The AsA content dropped from 430.62 μg/g in the CK group to 322.19 μg/g in the RT group, while in the ABA group, it decreased to 138.28 μg/g. TSS, sucrose content, and water soluble pectin (WPS) content followed change trends opposite to those of starch content. TSS increased from 6.55% in the CK group to 9.44% in the RT group, and rapidly increased to 16.03% in the ABA group. Similarly, the sucrose content increased from 5.22 mg/g in the CK group to 5.67 mg/g in the RT group, and abruptly to 14.93 mg/g in the ABA group. The WPS content in both RT and ABA groups increased, but not significantly. The amylases are key enzymes in the starch degradation pathway, and the polygalacturonase is involved in fruit ripening by breaking down pectin in cell wall^31^. The α-amylase activity significantly increased in both RT and ABA groups, showing an opposite pattern to that of the starch content, while the β-amylase and polygalacturonase (PG) activities decreased in the RT group. Fruit ripening also involves the associated cellular oxidation and peroxidation processes, as well as the activity changes of defense-related enzymes^32^. Both phenylalnine ammonialyase (PAL) and peroxidase (POD) activities markedly decreased in the RT group but slightly increased in the ABA group. The superoxide dismutase (SOD) activity showed a slight increase in both RT and ABA groups without any obvious differences.

### High-throughput sequencing and genome-wide identification of lncRNA in kiwifruit

A total of 288,869,672 raw sequence reads were obtained from CK, RT, and ABA-treated samples using strand-specific RNA-seq to investigate the underlying changes in genes and lncRNAs during kiwifruit ripening and softening. More than 96% of raw reads were clean reads. Of these clean reads, 68.68%, 70.85%, and 62.39% were mapped reads in the ABA, RT, and CK groups, respectively, and 66.20%, 68.45%, and 59.63% were unique mapped reads (Table S1). After matching with the kiwifruit reference genome, 28,976 known mRNAs, 12,388 novel mRNAs, and 27,402 lncRNAs were identified.

**Figure 2:**
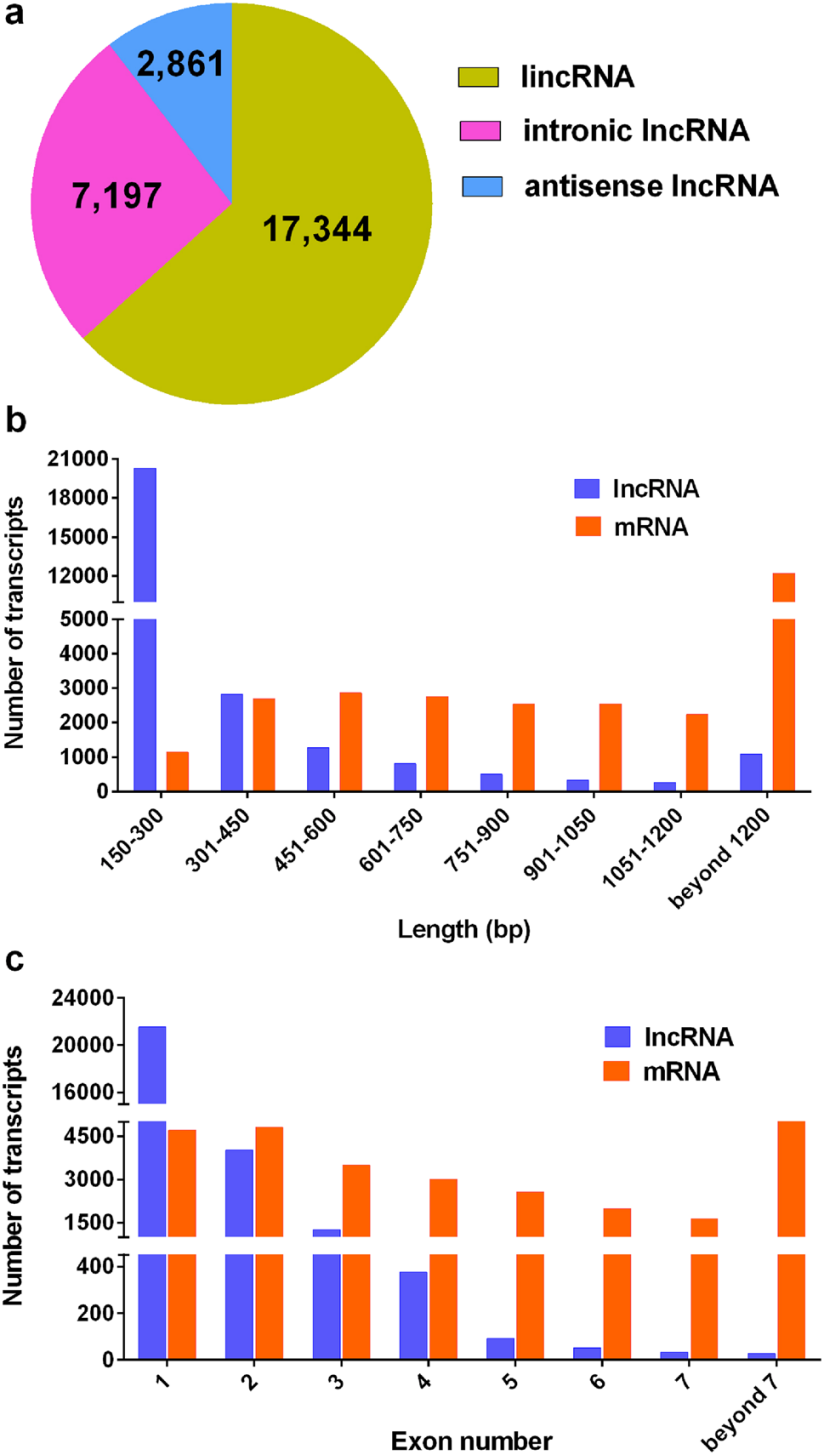
Classification, length distribution, and exon number of lncRNAs and mRNAs in *A. deliciosa* “Miliang-1”. (**a**) Pie chart of lncRNAs categorized as lincRNAs, intronic lncRNAs, and antisense lncRNAs. (**b**) Length distribution of lncRNAs and mRNAs. (**c**) Exon numbers of lncRNAs and mRNAs.

Further, 27,402 lncRNAs were classified into three types (lincRNAs, intronic lncRNAs, and antisense lncRNAs) based on their genomic positions and orientations relative to adjacent protein-coding genes. A majority of lncRNAs (63.29%) were located in intergenic regions, while only 26.26% and 10.44% were intronic lncRNAs and antisense lncRNAs, respectively (Fig. 2a). Moreover, the length and exon number distributions of lncRNAs were compared with those of known mRNAs. The results showed that 84.40% of lncRNAs were 150–450 bp, 11.60% were 451–1200 bp, and only 4.0% were > 1200 bp in length. In contrast, 42.12% of mRNAs were > 1200 bp, 44.64% were 451–1200 bp, and only 13.24% were 150–450 bp in length (Fig. 2b). Additionally, 93.29% of lncRNAs harbored only one or two exons, whereas 67.13% of mRNAs contained more than two exons (Fig. 2c).

### Expression profiles of genes and lncRNAs during kiwifruit ripening and softening

**Figure 3:**
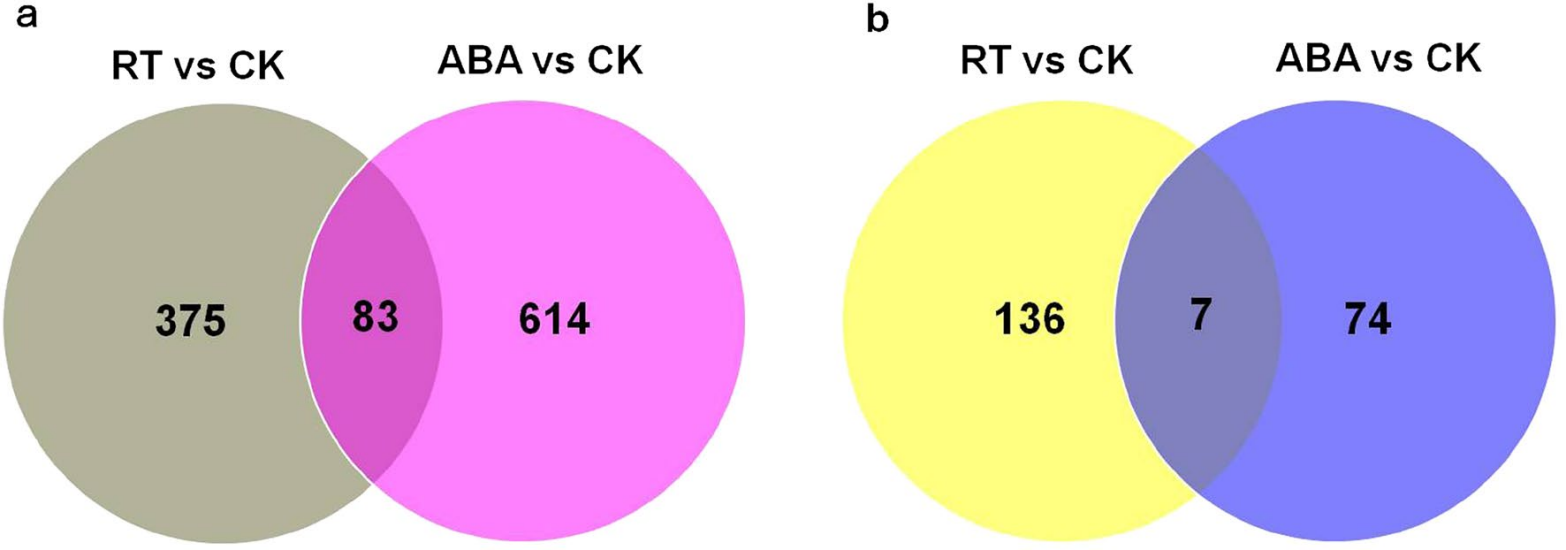
Venn diagrams of differentially expressed genes (**a**) and lncRNAs (**b**).

**Figure 4:**
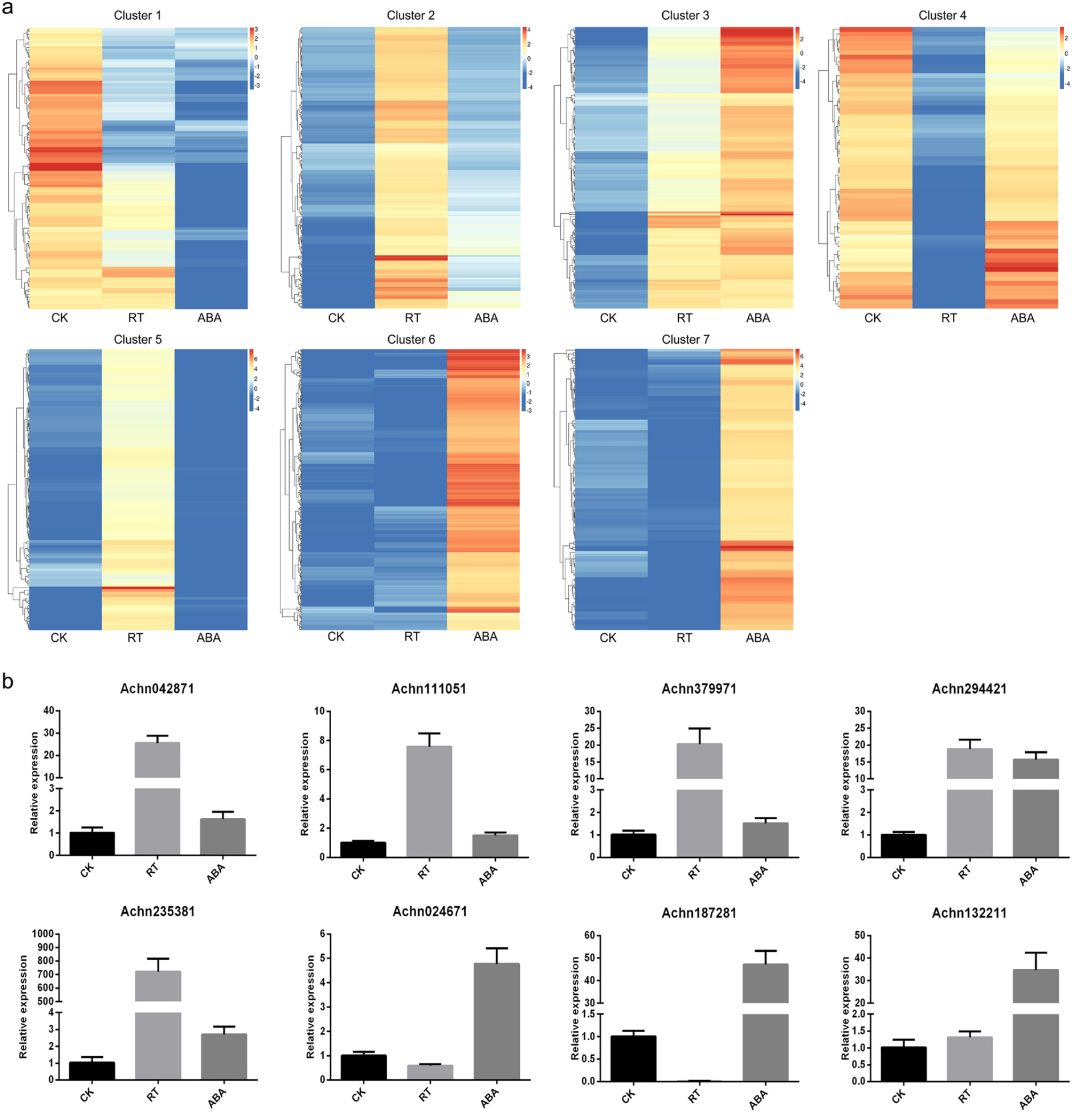
Clustering analysis (**a**) and qRT-PCR validation (**b**) of differentially expressed genes in kiwifruit. Achn042871, mannan endo-1,4-β-mannosidase 2; Achn111051, NAC transcription factor; Achn379971, cytochrome P450; Achn294421, β-galactosidase; Achn235381, MADS-box domain protein; Achn024671 and Achn187281, ethylene response factor; Achn132211, peroxidase. CK, freshly harvested fruits (fruit maturity stage); RT, fruits stored at 25 °C for 7 days (postharvest ripening stage); ABA, fruits treated with 50 mg/L ABA for 2 min and then stored at 25 °C for 7 days (softening stage).

After the expression level for each gene was normalized to fragments per kilobase of exon per million fragments mapped (FPKM), 458 and 697 DEGs were identified while comparing RT vs. CK (Table S2) and ABA vs. CK (Table S3), respectively. The Venn diagram in Fig. 3a showed the number of shared and exclusive DEGs between groups. Only 83 (7.74%) of the DEGs were differentially expressed in both comparisons, while 375 (34.98%) were exclusively expressed in RT vs. CK comparison and 614 (57.28%) in ABA vs. CK comparison. As shown in Fig. 4a, the expression patterns of DEGs in the three groups were divided into seven major categories. DEGs in cluster 1 exhibited a declining expression trend during kiwifruit ripening and softening, while the expression levels of DEGs in clusters 2 and 3, such as the mannan endo-1,4-β-mannosidase 2-like gene (Achn042871), NAC transcription factor gene (Achn111051), cytochrome P450 gene (Achn379971), and β-galactosidase gene (Achn294421; Fig. 4b), were upregulated in both RT and ABA groups. The expression levels of DEGs in cluster 4 were downregulated while the expression levels of DEGs in cluster 5, such as Achn235381 (Fig. 4b), were significantly upregulated in only the RT group. Also, 438 DEGs were highly expressed in the ABA group but suppressed in the CK and RT groups (clusters 6 and 7), although the expression trends in clusters 6 and 7 were somewhat different (Fig. S1). These genes might be the reason for the promoting effect of ABA on kiwifruit softening. For example, the expression levels of ethylene response factor genes (Achn024671 and Achn187281) and peroxidase gene (Achn132211) were markedly upregulated in only the ABA group (Fig. 4b). Ethylene response factors, which control the final step in ethylene signaling, played roles in the transcriptional regulation of ripening-related genes and in the regulation of kiwifruit-ripening processes^17,33^. The peroxidase, acting as an enzymatic antioxidant, catalyzes the redox reactions for a wide range of substrates during fruit growth and ripening^34^.

**Figure 5:**
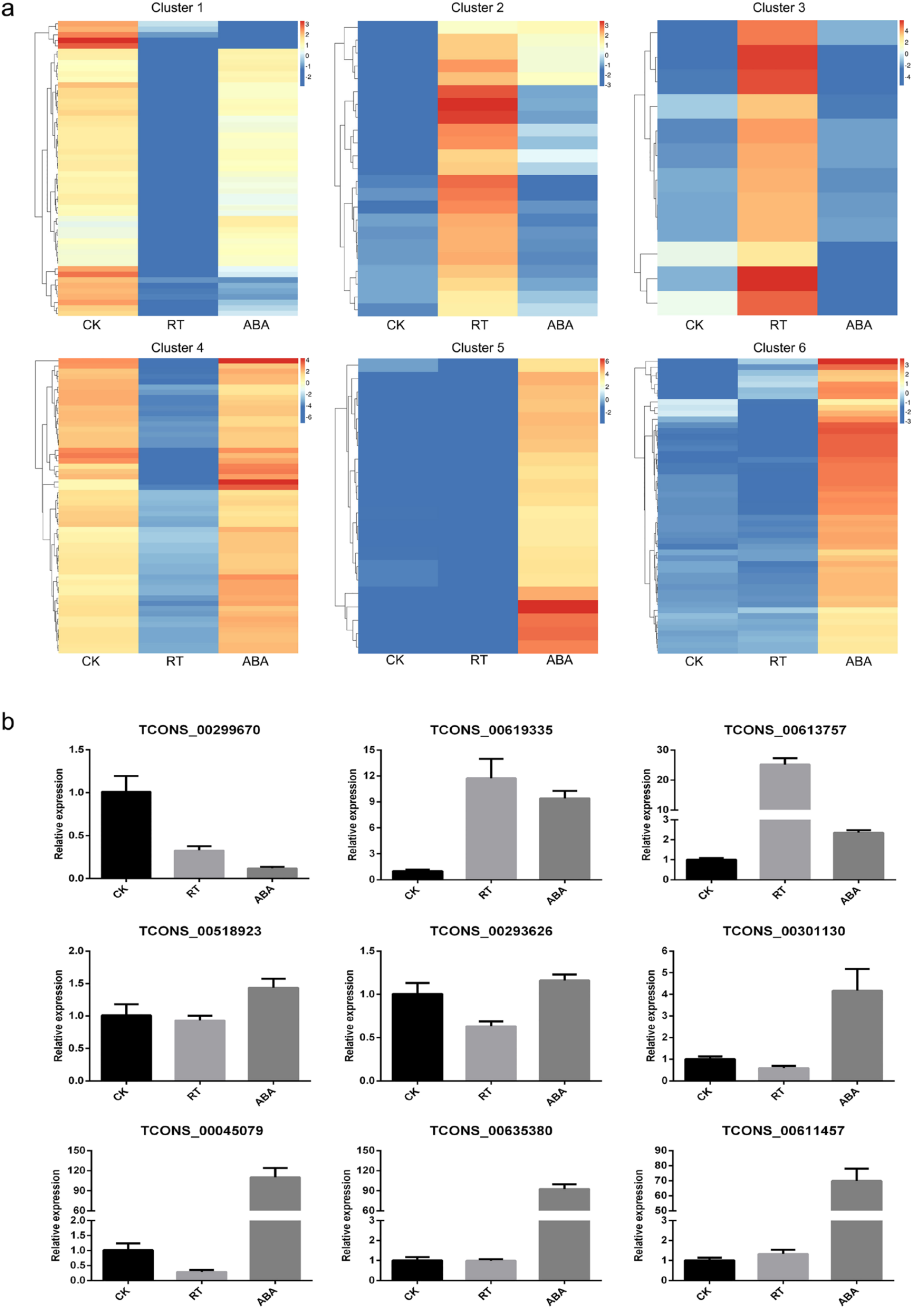
Clustering analysis (**a**) and qRT-PCR validation (**b**) of differentially expressed lncRNAs in kiwifruit. CK, freshly harvested fruits (fruit maturity stage); RT, fruits stored at 25 °C for 7 days (postharvest ripening stage); ABA, fruits treated with 50 mg/L ABA for 2 min and then stored at 25 °C for 7 days (softening stage).

The intersections among lncRNAs detected in the three samples were shown in Fig. 3b. A total of 143 and 81 lncRNAs were found to be differentially expressed in the comparisons of RT vs. CK (Table S4) and ABA vs. CK (Table S5), respectively. Seven (3.23%) of the DELs were differentially expressed in all groups, while 136 (62.67%) DELs were exclusively expressed in RT vs. CK and 74 (34.10%) in ABA vs. CK. The cluster analysis showed that DELs could be divided into six different expression patterns in the three samples (Fig. 5a). DELs in cluster 1, such as TCONS_00299670 (Fig. 5b), were downregulated in both RT and ABA groups. On the contrary, DELs in cluster 2, such as TCONS_00619335 and TCONS_00613757 (Fig. 5b), were upregulated in both RT and ABA groups. DELs in cluster 3 showed high expression in the RT group, while DELs in cluster 4 were suppressed in the RT group, such as TCONS_00518923 and TCONS_00293626 (Fig. 5b). Although the expression trends in clusters 5 and 6 were somewhat different (Fig. S2), the expression levels of DELs in clusters 5 and 6, such as TCONS_00301130, TCONS_00045079, TCONS_00635380, and TCONS_00611457 (Fig. 5b), were obviously upregulated in only ABA-treated fruits, indicating that these lncRNAs might be associated with the promoting effect of ABA on kiwifruit softening.

### Enrichment analyses of differentially expressed genes

**Figure 6:**
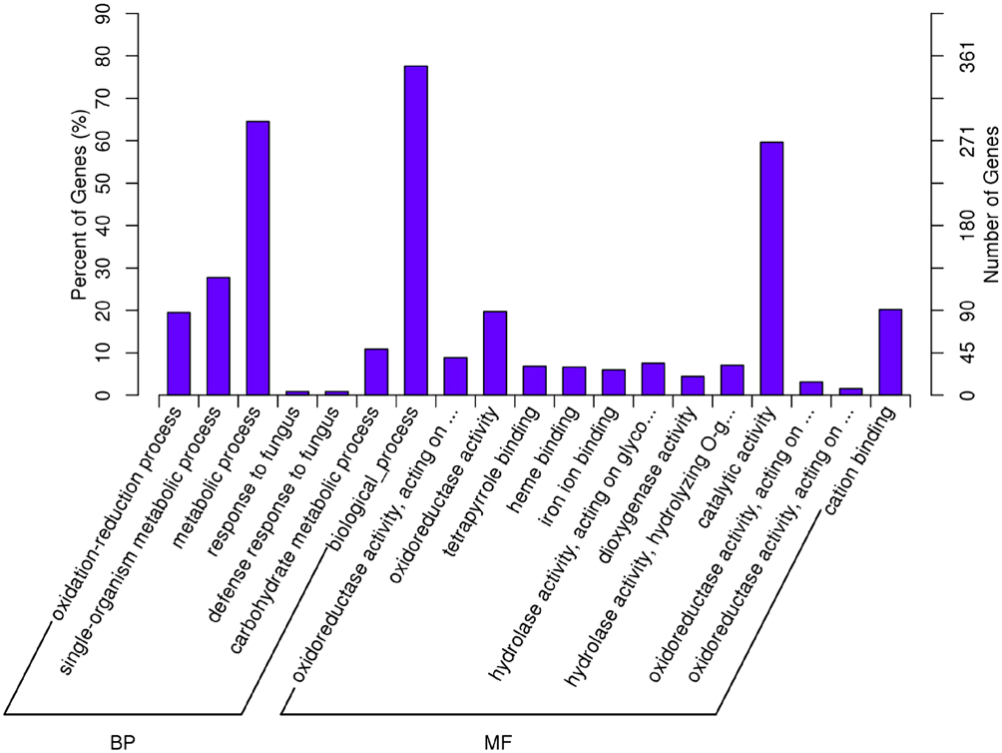
Gene Ontology analysis of differentially expressed genes in ABA vs. CK.

Gene Ontology (GO) and Kyoto Encyclopedia of Genes and Genomes (KEGG) analyses were performed to illuminate the potential functions of DEGs. In ABA vs. CK, DEGs associated with biological processes and molecular function were significantly enriched (Fig. 6). In the category of biological processes, seven terms including oxidation–reduction process, single-organism metabolic process, metabolic process, response to fungus, defense response to fungus, carbohydrate metabolic process, and biological process, were significantly enriched. In the category of molecular function, 12 terms were significantly enriched, including oxidoreductase activity acting on paired donors, oxidoreductase activity, tetrapyrrole binding, heme binding, iron ion binding, hydrolase activity acting on glycosyl bonds, dioxygenase activity, hydrolase activity hydrolyzing O-glycosyl compounds, catalytic activity, oxidoreductase activity acting on paired donors, oxidoreductase activity acting on single donors, and cation binding. The KEGG analysis revealed that 131 DEGs in ABA vs. CK were enriched in 70 pathways (Table S6). The most frequently predicted pathways were involved in phenylalanine metabolism, phenylpropanoid biosynthesis, brassinosteroid biosynthesis, biosynthesis of secondary metabolites, linoleic acid metabolism, alpha-linolenic acid metabolism, plant-pathogen interaction, carotenoid biosynthesis, alanine, aspartate and glutamate metabolism, flavonoid biosynthesis, starch and sucrose metabolism, and plant hormone signal transduction, and so forth.

Although no enriched GO term was identified in RT vs. CK, 57 DEGs were found to be enriched in 49 pathways using the KEGG database (Table S7). The top 20 enriched pathways included starch and sucrose metabolism; brassinosteroid biosynthesis; linoleic acid metabolism; plant circadian rhythm; flavonoid biosynthesis; carotenoid biosynthesis; alpha-linolenic acid metabolism; biosynthesis of secondary metabolites; amino sugar and nucleotide sugar metabolism; pentose and glucuronate interconversions; glutathione metabolism; cysteine and methionine metabolism; glycerolipid metabolism; plant hormone signal transduction; galactose metabolism; glycolysis/gluconeogenesis; limonene and pinene degradation; stilbenoid, diarylheptanoid, and gingerol biosynthesis; other glycan degradation; and histidine metabolism.

More important, in the ABA group, 4 DEGs were found to encode key enzymes related to ethylene biosynthesis, 17 related to ethylene signaling pathway, 11 related to starch and sucrose degradation, 24 related to cell wall degradation, and 3 related to membrane lipid peroxidation (Table S8). However, in the RT group, only 4, 3, 10, 7, and 2 DEGs, respectively, were found to encode enzymes involved in ethylene biosynthesis, ethylene signaling pathway, starch and sucrose degradation, cell wall degradation, and membrane lipid peroxidation (Table S8). Additionally, three genes (Achn315541, Achn338411, and Achn143751) associated with brassinosteroid biosynthesis were differentially expressed in kiwifruit after ABA treatment, while only two (Achn379971 and Achn143751) showed differential expression in the RT group. Moreover, 12 DEGs related to phenylalanine metabolism were identified after ABA treatment, while only one was found in the RT group. Transcription factor genes, such as *bZIP*, *MYB*, *NAC*, and *WRKY*, showed differential expression in both ABA and RT groups (Table S8). A zinc finger protein gene (Achn014701) showed significantly upregulated expression in ABA-treated fruits, which was newly found to interact physically with the β-amylase promoter and thus participated in the starch degradation of kiwifruit^35^.

### LncRNA target prediction and functional annotation

**Figure 7:**
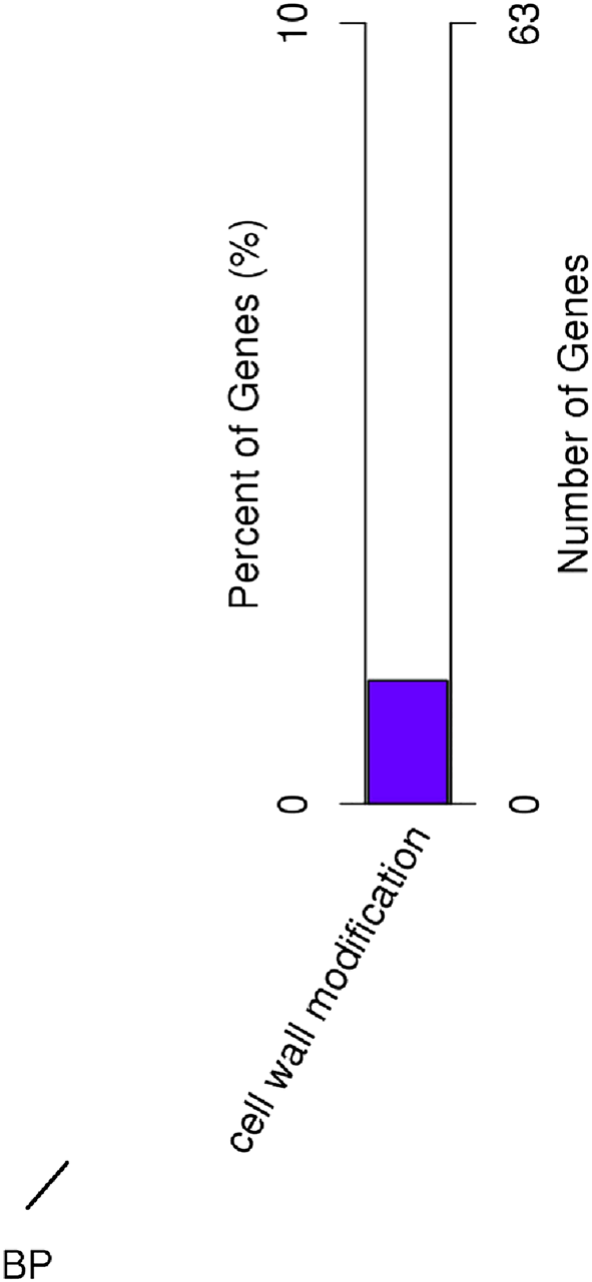
Gene Ontology analysis of the target genes of lncRNAs in ABA vs. CK.

**Figure 8:**
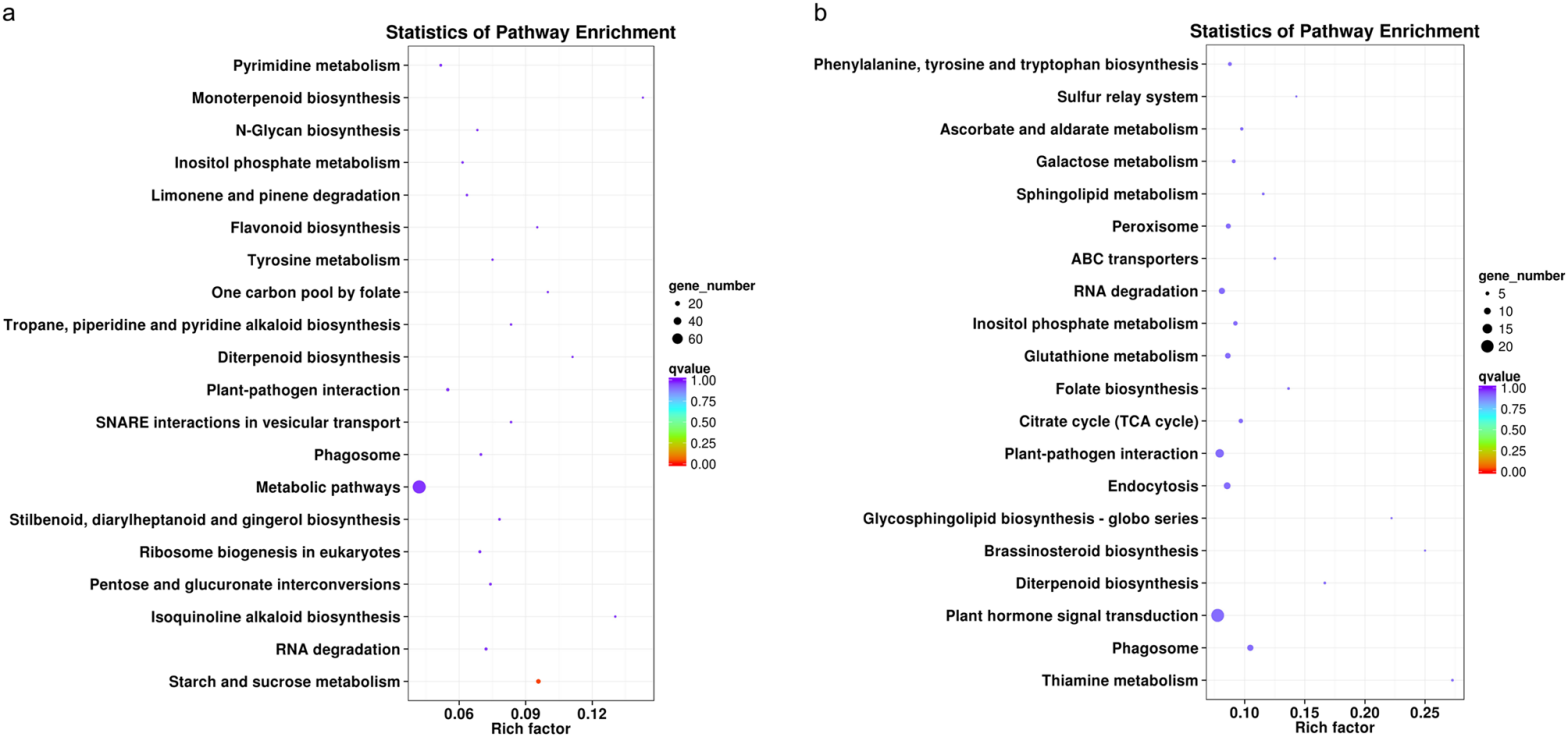
Kyoto Encyclopedia of Genes and Genomes analysis of target genes of DELs in ABA vs. CK (**a**) and RT vs. CK (**b**). The dot size indicates the number of enriched target genes of DELs in each pathway, and the different color of the dot represents the corrected *P*-value of each pathway. The top 20 enriched KEGG terms ranked by the corrected *P*-value are shown.

LncRNAs were found to be preferentially located next to genes they regulated^36^. To reveal potential functions of lncRNAs in kiwifruit ripening and softening, putative target genes of DELs were carefully analyzed. The GO analysis of predicated target genes of DELs in ABA vs. CK showed that 10 putative *cis*-target genes of lncRNAs were significantly enriched for the cell wall modification process (Fig. 7). The KEGG pathway analysis showed that ABA treatment significantly changed the expression of lncRNAs regulating genes involved in starch and sucrose metabolism (Fig. 8a). Besides, the target genes of DELs in ABA vs. CK were also associated with isoquinoline alkaloid biosynthesis, RNA degradation, pentose and glucuronate interconversions, plant-pathogen interaction, diterpenoid biosynthesis, flavonoid biosynthesis, N-glycan biosynthesis, monoterpenoid biosynthesis, and so on (Table S9). However, the GO analysis of the target genes of DELs in RT vs. CK identified no enriched GO terms. The KEGG enrichment analysis revealed that the most frequently predicted pathways were involved in thiamine metabolism, plant hormone signal transduction, diterpenoid biosynthesis, brassinosteroid biosynthesis, plant-pathogen interaction, glutathione metabolism, peroxisome, galactose metabolism, ascorbate and aldarate metabolism, and so forth (Fig. 8b and Table S10).

### Co-localization networks of the DELs and DEGs

**Figure 9:**
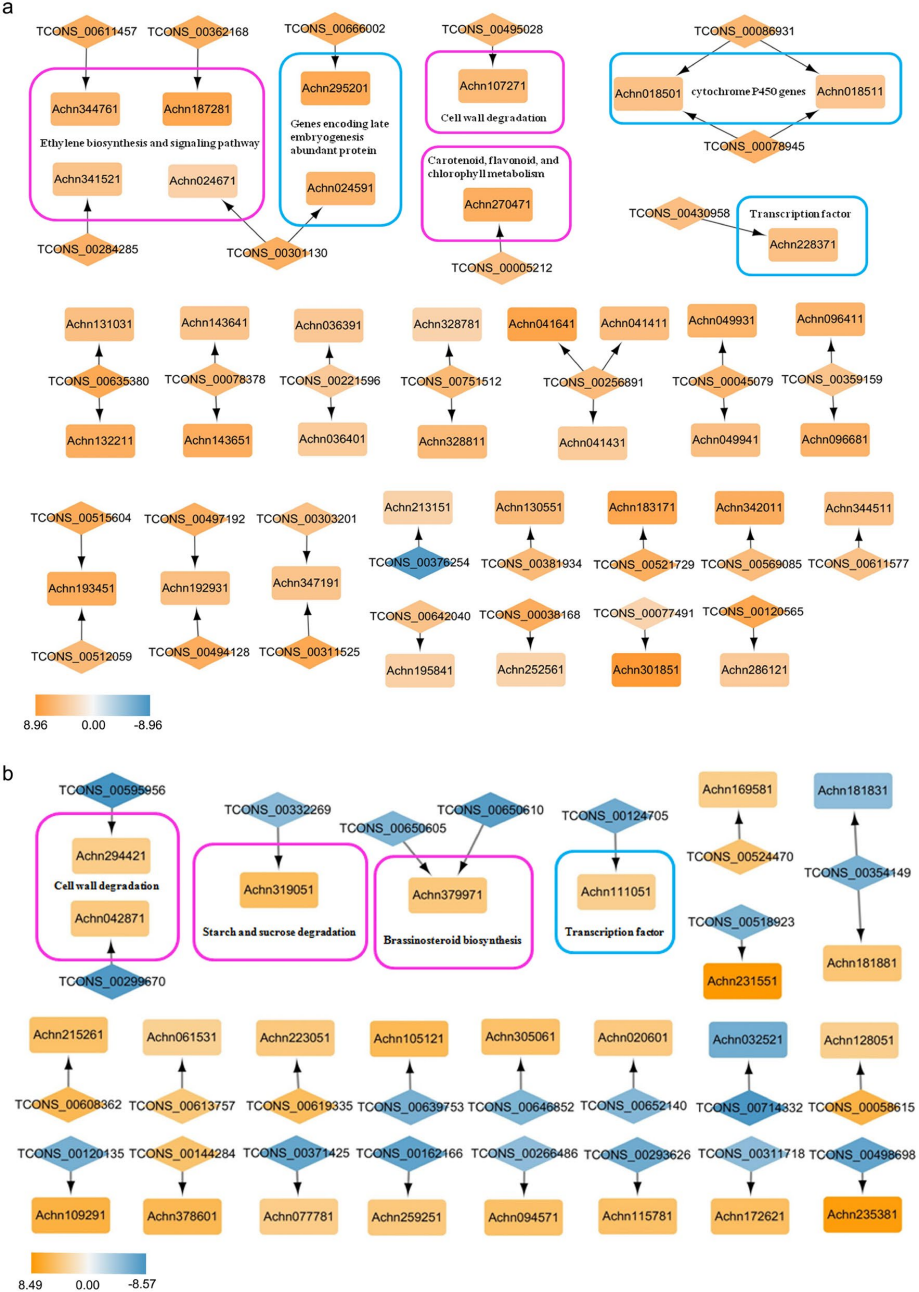
Co-localization network of DELs and DEGs in ABA vs. CK (**a**) and RT vs. CK (**b**). DELs were marked with diamonds, and DEGs were marked with rectangles. Upregulated genes were indicated in orange, while downregulated genes were indicated in blue. The orientations of the predicted regulatory relationship were indicated by the directions of the arrows.

The co-localization networks of the DELs and DEGs were constructed using Cytoscape 3.7.2 to further understand the regulation roles of lncRNAs in kiwifruit ripening and softening. A total of 31 DELs and 38 DEGs were identified in ABA vs. CK, and 42.86% of the regulated relationships had more than two nodes as shown in Fig. 9a, indicating the complexity of ABA-regulated softening. Except TCONS_00376254, all DELs showed upregulated expression levels like their target genes. Notably, three ethylene response factor genes (Achn024671, Achn187281, and Achn344761) related to the ethylene signaling pathway were targeted by TCONS_00301130, TCONS_00362168, and TCONS_00611457, respectively. One ACC oxidase gene (Achn341521) involved in ethylene biosynthesis was targeted by TCONS_00284285, and one secondary cell wall-related glycosyltransferase gene (Achn107271) associated with cell wall degradation was targeted by TCONS_00495028. Additionally, two cytochrome P450 genes (Achn018501 and Achn018511), which were reported to participate in avocado fruit ripening^37^, were targeted by both TCONS_00086931 and TCONS_00078945. Another two genes encoding late embryogenesis abundant proteins (Achn024591 and Achn295201), responding to brassinosteroids and ABA during the development of fruits in *Fragaria chiloenisis*^38^, were targeted by TCONS_00301130 and TCONS_00666002, respectively. A p-coumarate 3-hydroxylase gene (Achn270471), associated with carotenoid, flavonoid, and chlorophyll metabolism, was targeted by TCONS_00005212. An *MYB* gene (Achn228371) and a peroxidase gene (Achn132211), respectively, were targeted by TCONS_00430958 and TCONS_00635380. Therefore, ABA might promote fruit softening by regulating the expression of aforementioned lncRNAs and their target genes.

In RT vs. CK, 25 DELs together with 25 DEGs were identified in the co-localization network, and 91.66% of the regulated relationships had fewer than three nodes as shown in Fig. 9b. More than half of the DELs in the RT group had expression levels opposite to those of their target genes. Moreover, two genes (β-galactosidase and mannan endo-1,4-β-mannosidase) related to cell wall degradation were targeted by TCONS_00595956 and TCONS_00299670, respectively. One gene (pectinesterase/pectinesterase inhibitor) involved in starch and sucrose degradation was targeted by TCONS_00332269. Besides, a cytochrome P450 gene (Achn379971) related to brassinosteroid biosynthesis was targeted by both TCONS_00650605 and TCONS_00650610. One transcription factor *NAC* (Achn111051) was targeted by TCONS_00124705. Additionally, the target genes of seven DELs expressed in all samples were analyzed (Table S11). Among these, TCONS_00330037 targeted a pectinesterase inhibitor gene (Achn102711); both of these were upregulated in the RT and ABA groups. Among the predicted target genes of TCONS_00781977, five were abscisic stress-ripening protein genes (Achn387301, Achn387331, Achn387341, Achn387351, and Achn387361), and three (Achn387301, Achn387331, and Achn387341) were differentially expressed. These targets of DELs, therefore, were considered to play fundamental roles in kiwifruit ripening.

## Discussion

Fruit texture has a great influence on postharvest storage and transportation. Most fruits undergo softening when pick from the tree, resulting from the degradation of cell wall and the decline of intracellular adhesion^39^. In some fruits, like kiwifruit and banana, starch degradation usually occurs in the initial stages of postharvest softening^3^. In this study, the starch content and fruit firmness of kiwifruit markedly decreased in both room temperature-stored and ABA-treated fruits, while their amylase activities significantly increased (Fig. 1). This is consistent with that genes involved in starch and sucrose metabolism increasingly expressed (Table S8). PG is an important hydrolytic enzyme responsible for pectin degradation and thus participates in cell wall degradation and fruit softening^40^. In kiwifruit “Donghong”, a total of 51 *PG* genes were identified, and 11 *PG* genes were highly or moderately expressed in softening fruit^11^. Here, one *PG* gene (Achn080501) showed significantly upregulated expression in ABA treated-fruits, while no differentially expressed *PG* genes was detected in the RT group. This is consistent with that the PG activity of the RT group was lower than that of the ABA group. Previous studies demonstrated crucial roles of ABA in kiwifruit softening^8^. The physiological properties of ABA-treated kiwifruit were compared with those of nontreated fruits (Fig. 1). In ABA-treated fruits, the firmness and starch and AsA contents sharply declined, while the TSS and sucrose contents, as well as the α-amylase activity, significantly increased. These results confirmed that ABA treatment promoted the softening of kiwifruit as previously stated.

Then, transcriptomes were obtained from ABA-treated (ABA) and room temperature (RT)-stored fruits, as well as freshly harvested fruits (CK). Further, 458 and 697 DEGs, respectively, were identified in RT vs. CK and ABA vs. CK (Fig. 3). The enrichment analysis revealed that these DEGs were mainly involved in the processes of starch and sucrose metabolism, brassinosteroid biosynthesis, plant hormone signal transduction, linoleic acid metabolism, flavonoid biosynthesis, and carotenoid biosynthesis (Tables S6 and S7). Quite a few genes have been demonstrated to play key roles in the ripening process of kiwifruit, such as lipoxygenase genes^41^, ethylene response factors^33^, and starch degradation-related genes^10^. DEGs involved in ethylene biosynthesis, ethylene signaling pathway, starch and sucrose metabolism, cell wall degradation, and membrane lipid peroxidation were present in both RT vs. CK and ABA vs. CK. However, gene numbers and exact members were somewhat different (Table S8), implying the involvement of different regulatory mechanisms in kiwifruit ripening and ABA-regulated softening. β-Galactosidase, acting as a cell wall-modifying enzyme, plays a crucial role in fruit ripening and softening^42^. qPCR validated that the expression level of β-galactosidase gene (Achn294421) was upregulated in both RT and ABA groups while the transcripts of two ethylene response factor genes (Achn024671 and Achn187281) increased in only the ABA group. He et al. reported that endogenous brassinosteroid contents gradually increased during persimmon fruit ripening; 24-epibrassinolide (EBR) treatment resulted in rapid fruit softening, and brassinazole (a brassinosteroid biosynthesis inhibitor) treatment delayed persimmon fruit ripening^43^. Four genes (Achn315541, Achn338411, Achn379971, and Achn143751) associated with brassinosteroid biosynthesis showed upregulated expression in the RT and/or ABA groups, suggesting the involvement of brassinosteroids in kiwifruit ripening and softening. Additionally, the increased expression levels of *bZIP* and *MYB*, as well as other transcription factor genes, suggested their participation in kiwifruit ripening and softening. However, their exact roles remain to be studied. In banana fruit, the *bZIP* was reported to participate in fruit ripening via activating the transcription of aroma biosynthetic genes^44^. Also, *MYBs* could bind to the promoters of cell wall degradation- and carotenoid biosynthesis-related genes to participate in papaya fruit softening and carotenoid accumulation^45^. A larger number of DEGs were detected in ABA vs. CK (Fig. 3), revealing that ABA treatment promoted the softening of kiwifruit by modulating the expression of more protein-coding genes.

A large number of studies showed critical roles of lncRNAs in the regulation of various biological processes in plants. Many lncRNAs have been identified from model plants, such as *Arabidopsis,* rice, maize, and tomato. However, studies on lncRNAs in kiwifruit were limited. Wang et al.^46^ has predicted 14,845 transcripts from 12,280 loci as putative lncRNAs based on transcriptomic analysis of *Pseudomonas syringae* pv. *actinidiae*-infected kiwifruit leaves. Although 7051 lncRNAs were identified from fruits of *A. chinensis* in three development stages^47^, the validation and discussion on the regulation of these lncRNAs involved in fruit ripening were very limited. In this study, 27,402 lncRNAs were identified in the comprehensive set of kiwifruit transcripts obtained from postharvest fruits in three stages (fruit maturity, ripening, and softening). Of these, 63.29% were lincRNAs, which was similar to the proportion of lincRNAs in rice^48^. The lncRNAs were shorter than known mRNAs as previously reported in other plants^49^, also the average number of exons was less.

LncRNAs have recently been found to regulate fruit development and ripening. In *Cucumis* melo, six lncRNAs were found to correlate with fruit ripening and climacteric, and might participate in the regulation of ethylene biosynthesis and metabolism and the ABA signaling pathway^50^. Ou et al.^51^ reported that 1066 lncRNAs were differentially expressed during pepper fruit development, and some of their target genes were associated with cell wall formation, carotenoid biosynthesis, and plant hormone signal transduction. In the present study, 143 and 81 lncRNAs, respectively, were found to be differentially expressed in the comparisons of RT vs. CK and ABA vs. CK (Fig. 3), indicating a possible involvement of lncRNAs in the regulation of kiwifruit ripening and softening. And the target genes of DELs were mainly involved in cell wall modification process, starch and sucrose metabolism, plant hormone signal transduction, and so forth. These results were consistent with previous reports that genes participating in cell wall degradation, starch and sucrose metabolism, and ethylene signal transduction were increasingly expressed during kiwifruit ripening and softening^2,10,11,52^. In ABA vs. CK, the 10 *cis*-target genes of DELs that significantly enriched for the cell wall modification process were pectinesterase genes. Pectinesterase catalyses the demethylesterification of cell-wall polygalacturonans, which promotes the hydrolysis of polygalacturonic acid by PG^19^. Moreover, the co-localization network showed that 25 DEGs and 25 DELs were present in RT vs. CK and 38 DEGs and 31 DELs in ABA vs. CK (Fig. 9). Genes related to fruit ripening, such as genes encoding β-galactosidase, mannan endo-1,4-β-mannosidase, pectinesterase/pectinesterase inhibitor, and NAC transcription factor, were present in the co-localization network. For example, TCONS_00595956 regulated the gene Achn294421 (β-galactosidase) that could alter galactose metabolism and promote ripening in tomato fruit^53^. XLOC_060185 *cis* targeted the gene Achn111051 (NAC transcription factor), which was reported to regulate pectin metabolism during fruit softening in *Fragaria chiloensis*^54^. These results revealed that lncRNAs regulated the expression levels of protein-coding genes during kiwifruit ripening. The last step in ethylene biosynthesis is the conversion of ACC to ethylene by the ACO enzyme^3^. Exogenous ABA treatment increased the ABA concentration and the expression levels of both *ACS* and *ACO* genes, promoting ethylene synthesis and fruit ripening in tomato^7^. Notably, several ethylene biosynthesis- and signaling pathway-related genes, including one *ACC* oxidase gene and three ethylene response factor genes, were found in the co-localization network of ABA vs. CK, suggesting ABA might promote ethylene biosynthesis and fruit softening by regulating the expression of these lncRNAs.

## Materials and methods

### Plant materials and treatments

Fruits of *A. chinensis* var. *deliciosa* “Miliang-1” were harvested from a commercial orchard in Fujian, China, with a mean TSS of 6.5%. Uniform and intact fruits without mechanical injuries were divided into three groups: CK, RT, and ABA. In the CK group, freshly harvested fruits with TSS of 6.0–7.0% (fruit maturity stage) were sampled. In the RT group, fruits stored at room temperature (25 °C) for 7 days with a mean TSS of 9.0–10.0% (postharvest ripening stage) were sampled. In the ABA group, fruits were soaked with 50 mg/L ABA for 2 min and then stored at 25 °C for 7 days with a mean TSS of 15.5–16.5% (softening stage) were sampled. Each group contained three biological replicates of approximately 100 fruits. The outer pericarps (without skin and seeds) of three fruits in each group were cut into small pieces and mixed, which were then rapidly frozen in liquid nitrogen and stored at − 80 °C for further experiments.

### Physiological properties of fruit

A number of kiwifruit postharvest properties were measured, including firmness, total soluble solid (TSS) content, ascorbic acid (AsA) content, sucrose content, water soluble pectin (WSP) content and enzyme activities of α-/β-amylase, polygalacturonase (PG), phenylalanine ammonialyase (PAL), superoxide dismutase (SOD) and peroxidase (POD). The firmness of 10 fruits without skin in each group was measured using a GY texture analyzer following the manufacturer’s protocol (Shsiwi, Zhejiang, China). The TSS content of 10 fruits in each group were detected using an digital hand-held refractometer (Atago, Japan). Each fruit was cut into four pieces, and then the juice was squeezed from each piece onto the refractometer. The contents of AsA, sucrose, and WSP, respectively, were measured on three replicates of 0.1-g frozen samples using an AsA assay kit, a sucrose assay kit, and a WSP assay kit provided by Suzhou Comin Biotechnology Co., Ltd (Jiangshu, China). The activities of α-/β-amylase, PG, PAL, SOD, and POD, respectively, were detected on three replicates of 0.1-g frozen samples using an α-amylase assay kit, a β-amylase assay kit, a PG assay kit, a PAL assay kit, an SOD assay kit, and a POD assay kit supplied by Suzhou Comin Biotechnology Co., Ltd. Fruit inclusions and enzyme activities were measured with three biological replicates. One-way ANOVA in the SPSS software was used for statistical analysis.

### Library construction and sequencing

Total RNAs were extracted from different samples using an RNAprep pure Plant Kit (Tiangen, Beijing, China). Then, RNA integrity was checked on 1% agarose gels. RNA quantity and quality were measured using a bioanalyzer system (Agilent Technologies, CA, USA). RNA purity and concentration, respectively, were checked using a NanoPhotometer spectrophotometer (Implen, CA, USA) and a Qubit RNA assay kit in a Qubit2.0 Flurometer (Life Technologies, CA, USA). Further, 3 µg RNA per sample was used as the input material to remove ribosomal RNA (rRNA) using an Epicentre Ribo-Zero rRNA removal kit (Epicentre, USA). After the rRNA free residue was cleaned up by ethanol precipitation, sequencing libraries were constructed using the rRNA-depleted RNA with an NEBNext Ultra Directional RNA library prep kit for Illumina (NEB, USA) following the manufacturer’s instructions. After assessing the library quality, a cBot cluster generation system was used to cluster the index-coded samples with a TruSeq PE cluster kit v3-cBot-HS (Illumina) following the manufacturer’s recommendations. Then, the libraries were sequenced on the Illumina HiseqX10 platform, and 150-bp paired-end reads were generated. The sequencing results were deposited in the Sequence Read Archive (http://www.ncbi.nlm.nih.gov/sra/) at the National Center for Biotechnology Information database under accession number PRJNA639739.

### Read mapping and transcriptome assembling

After removing reads containing the adapter or ploy-N as well as low quality reads, clean data (clean reads) were obtained and mapped to the kiwifruit reference genome^30^ using TopHat2 software^55^. The mapped reads were then used for reference-guided assemblies with both Scripture^56^ and Cufflinks^57^. The assembled transcripts from the three groups were then merged together to produce a final transcriptome.

All assembled transcripts were predicted with coding potential using Coding Potential Calculator (CPC)^58^ and Pfamscan (PFAM)^59^. These transcripts, without coding potential and longer than 200 bp, were selected as long noncoding RNAs (lncRNAs) by a previously described method^60^. Then, culffcompare software was used to analyze different types of lncRNAs, including long intergenic noncoding RNAs (lincRNAs), intronic lncRNAs, and antisense lncRNAs.

### Differentially expressed genes and lncRNAs

The expression levels of genes and lncRNAs were measured with normalized counts of reads by their respective lengths using Cufflinks^57^. Fragments per kilobase of exon per million fragments mapped (FPKM) was applied to represent the normalized expression value of genes and lncRNAs. DEGseq package was used to identify the DEGs and DELs in two groups with a corrected *P* value < 0.05 and an absolute value of log_2_FC > 3. The FPKM of RT (or ABA) divided by the FPKM of CK was described as a fold change (FC) expression level. Log_2_-transformed FPKM values of the differential expressed genes and lncRNAs were used for K-means clustering in BMKCloud software (http://www.biocloud.net).

### Prediction of lncRNA target genes

One of the main functions of lncRNAs was to regulate the expression of neighboring coding genes^29,61^. Thus, coding genes within 100 kb upstream or downstream of the lncRNAs were selected as their target genes. Interaction networks between differential expressed genes and lncRNAs were constructed according to their genomic co-localizations using Cytoscape software.

### Enrichment analysis

Gene Ontology (GO) enrichment analyses of the differential expression genes and target genes of lncRNAs were performed using the GOseq R package. GO terms with the corrected *P*-value < 0.05 were considered significantly enriched. KOBAS software was used to test the statistical enrichment of DEGs and target genes of DELs based on the KEGG database^62,63^.

### Real-time quantitative PCR analysis

Eight differential expression genes and nine differential expression lncRNAs were randomly selected to perform real-time quantitative PCR (qPCR) to detect their relative expression levels so as to validate the results obtained by RNA-seq. Total RNAs from different samples were reverse transcribed with a PrimeScript RT reagent kit (Perfect Real Time; TaKaRa, Japan) into cDNAs for qPCR analysis. The actin gene (Achn107181) was used as an internal control for the normalization of the qPCR data. Gene expression was analyzed using an Eppendorf PCR detection instrument with TB Green Premix Ex Taq II (Tli RNaseH Plus; TaKaRa, Japan) following the manufacturer’s protocol. PCR reactions included an initial denaturation at 95 °C for 30 s, followed by 40 cycles at 95 °C for 5 s, 60 °C for 20 s, and 72 °C for 10 s, as well as a default melt curve program. The relative expression levels of genes were calculated using the 2^−∆∆Ct^ method. Each sample was analyzed with three biological replicates. The primers used in this study were summarized in Table S12.

## Supplementary Information

Supplementary Figure S1.

Supplementary Figure S2.

Supplementary Table S1.

Supplementary Table S2.

Supplementary Table S3.

Supplementary Table S4.

Supplementary Table S5.

Supplementary Table S6.

Supplementary Table S7.

Supplementary Table S8.

Supplementary Table S9.

Supplementary Table S10.

Supplementary Table S11.

Supplementary Table S12.
