# Supplementary material for: Integrated analysis of lncRNA and mRNA transcriptomes reveals the potential regulatory role of lncRNA in kiwifruit ripening and softening
Source: Sci Rep. 2021 Jan 18;11:1671. doi: 10.1038/s41598-021-81155-1 (PMC7814023; doi:10.1038/s41598-021-81155-1)
Supplement: Supplementary file 2 — Supplementary Figure S2. [file 41598_2021_81155_MOESM2_ESM.doc]

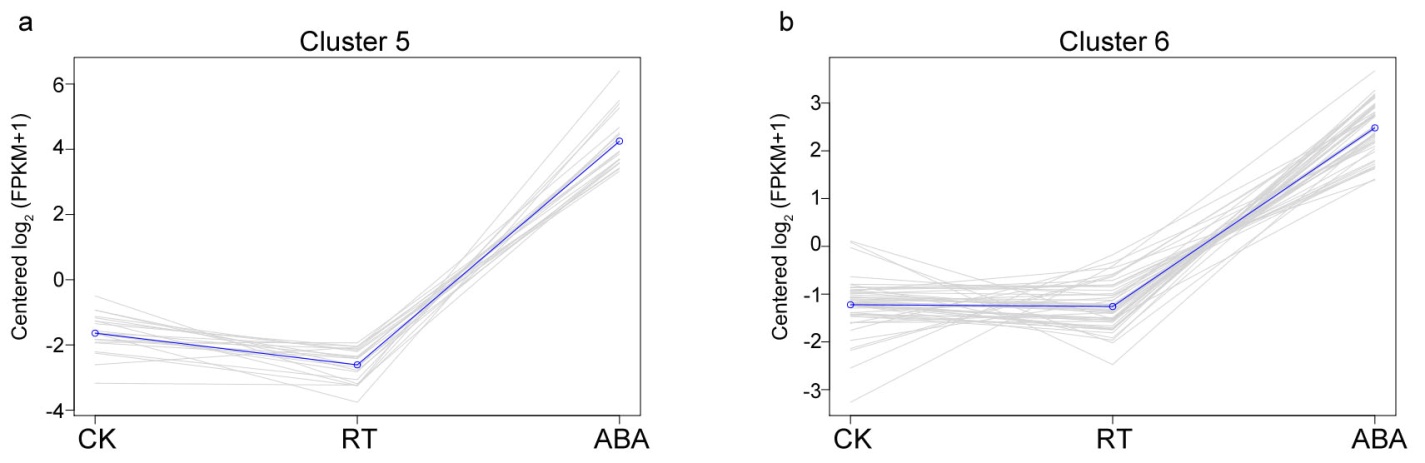


Figure S2. Expression trend of DELs in cluster 5 (a) and cluster 6 (b). The abscissa represents the samples, and the ordinate represents the expression level normalized by the centered logarithmic value. A gray line indicates the expression trend of a lncRNA, and the blue line indicates the expression trend of this type of lncRNAs.
