# Supplementary material for: Integrated analysis of lncRNA and mRNA transcriptomes reveals the potential regulatory role of lncRNA in kiwifruit ripening and softening
Source: Sci Rep. 2021 Jan 18;11:1671. doi: 10.1038/s41598-021-81155-1 (PMC7814023; doi:10.1038/s41598-021-81155-1)
Supplement: Supplementary file 3 — Supplementary Table S1. [file 41598_2021_81155_MOESM3_ESM.doc]

**Table S1.** Statistical data of the RNA-Seq reads for three samples

| **Samples** | **Raw reads** | **Clean reads** | **Clean bases** | **Q20(%)^a^** | **Q30(%)^b^** | **Mapped reads** | **Unique mapped reads** |
| --- | --- | --- | --- | --- | --- | --- | --- |
| ABA | 94,635,972 | 92,813,106 | 13.92G | 96.97 | 92.49 | 63,746,019 (68.68%) | 61,444,302 (66.20%) |
| RT | 93,051,346 | 89,889,436 | 13.48G | 96.22 | 90.83 | 63,689,387 (70.85%) | 61,526,914 (68.45%) |
| CK | 101,182,354 | 96,299,434 | 14.44G | 97.41 | 93.39 | 60,085,895 (62.39%) | 57,427,356 (59.63%) |

^a^ A Q20 value is the percentage of bases with a Phred value greater than 20 to the total bases; greater than 95% indicates that the sequencing result is of high quality.

b A Q30 value is the percentage of bases with a Phred value greater than 30 to the total bases; greater than 90% indicates that the sequencing result is of high quality.
