# Supplementary material for: Integrated analysis of lncRNA and mRNA transcriptomes reveals the potential regulatory role of lncRNA in kiwifruit ripening and softening
Source: Sci Rep. 2021 Jan 18;11:1671. doi: 10.1038/s41598-021-81155-1 (PMC7814023; doi:10.1038/s41598-021-81155-1)
Supplement: Supplementary file 4 — Supplementary Table S2. [file 41598_2021_81155_MOESM4_ESM.doc]

**Table S2. Differentially expressed genes in RT vs CK**

| **Transcript ID** | **Gene ID** | **FPKM (CK)** | **FPKM (RT)** | **FPKM (ABA)** | **log_2_ FPKM (RTvsCK)** | **corrected *P* value (RTvsCK)** | **log_2_ FPKM (ABAvsCK)** | **corrected *P* value (ABAvsCK)** |
| --- | --- | --- | --- | --- | --- | --- | --- | --- |
| Achn002181 | Achn002181 | 2.46 | 49.48 | 5.07 | 4.33 | 0.0065 | 1.04 | 0.6939 |
| Achn003701 | Achn003701 | 9.05 | 178.51 | 6.54 | 4.30 | 0.0170 | -0.47 | 0.9249 |
| Achn006311 | Achn006311 | 11.55 | 137.84 | 6.90 | 3.58 | 0.0217 | -0.74 | 0.7925 |
| Achn008991 | Achn008991 | 5.83 | 249.21 | 4.22 | 5.42 | 0.0003 | -0.47 | 0.9254 |
| Achn009551 | Achn009551 | 0.73 | 23.67 | 0.26 | 5.01 | 0.0128 | -1.48 | 0.7310 |
| Achn009751 | Achn009751 | 0.44 | 23.13 | 0.24 | 5.73 | 0.0002 | -0.87 | 1.0000 |
| Achn010181 | Achn010181 | 11.45 | 0.09 | 1.38 | -7.01 | 0.0040 | -3.05 | 0.4242 |
| Achn011431 | Achn011431 | 1.92 | 30.35 | 0.29 | 3.99 | 0.0422 | -2.71 | 0.4242 |
| Achn011961 | Achn011961 | 1.53 | 64.61 | 2.07 | 5.40 | 0.0002 | 0.43 | 0.9257 |
| Achn014271 | Achn014271 | 1.10 | 38.65 | 0.99 | 5.14 | 0.0014 | -0.15 | 0.9832 |
| Achn015151 | Achn015151 | 0.80 | 26.67 | 14.64 | 5.06 | 0.0193 | 4.19 | 0.1334 |
| Achn016841 | Achn016841 | 17.25 | 312.93 | 113.91 | 4.18 | 0.0027 | 2.72 | 0.2027 |
| Achn021371 | Achn021371 | 5.43 | 65.99 | 0.46 | 3.60 | 0.0329 | -3.55 | 0.2466 |
| Achn023381 | Achn023381 | 0.27 | 41.99 | 0.51 | 7.29 | 0.0006 | 0.94 | 0.8729 |
| Achn025461 | Achn025461 | 1.73 | 77.83 | 2.47 | 5.49 | 0.0023 | 0.52 | 0.9251 |
| Achn026081 | Achn026081 | 2.96 | 51.62 | 7.05 | 4.13 | 0.0154 | 1.25 | 0.5928 |
| Achn029661 | Achn029661 | 66.73 | 2.12 | 34.54 | -4.98 | 0.0019 | -0.95 | 0.6391 |
| Achn030261 | Achn030261 | 5.58 | 0.05 | 0.92 | -6.80 | 0.0066 | -2.59 | 0.4242 |
| Achn032521 | Achn032521 | 16.28 | 0.20 | 44.78 | -6.36 | 0.0170 | 1.46 | 0.4381 |
| Achn035981 | Achn035981 | 7.76 | 332.64 | 30.23 | 5.42 | 0.0001 | 1.96 | 0.4242 |
| Achn036081 | Achn036081 | 5.67 | 183.95 | 2.16 | 5.02 | 0.0006 | -1.39 | 0.5737 |
| Achn037231 | Achn037231 | 17.70 | 172.43 | 21.74 | 3.28 | 0.0448 | 0.30 | 0.9387 |
| Achn038551 | Achn038551 | 4.04 | 106.18 | 15.96 | 4.71 | 0.0033 | 1.98 | 0.4242 |
| Achn042871 | Achn042871 | 10.34 | 175.62 | 26.20 | 4.09 | 0.0048 | 1.34 | 0.4419 |
| Achn046761 | Achn046761 | 25.19 | 317.74 | 34.58 | 3.66 | 0.0148 | 0.46 | 0.8824 |
| Achn046891 | Achn046891 | 35.58 | 460.44 | 46.11 | 3.69 | 0.0138 | 0.37 | 0.9183 |
| Achn048101 | Achn048101 | 5.62 | 149.21 | 32.10 | 4.73 | 0.0043 | 2.51 | 0.4242 |
| Achn048111 | Achn048111 | 0.74 | 67.57 | 6.98 | 6.52 | 0.0109 | 3.24 | 0.4242 |
| Achn049441 | Achn049441 | 5.62 | 95.39 | 9.94 | 4.09 | 0.0075 | 0.82 | 0.7690 |
| Achn049921 | Achn049921 | 35.15 | 2.41 | 27.35 | -3.87 | 0.0478 | -0.36 | 0.9206 |
| Achn057591 | Achn057591 | 17.03 | 1.00 | 7.41 | -4.09 | 0.0166 | -1.20 | 0.4911 |
| Achn060041 | Achn060041 | 11.83 | 777.85 | 21.73 | 6.04 | 0.0000 | 0.88 | 0.7339 |
| Achn061531 | Achn061531 | 20.21 | 222.79 | 9.67 | 3.46 | 0.0262 | -1.06 | 0.5700 |
| Achn061621 | Achn061621 | 0.17 | 14.40 | 0.48 | 6.43 | 0.0126 | 1.52 | 1.0000 |
| Achn062351 | Achn062351 | 3.63 | 47.64 | 0.98 | 3.71 | 0.0338 | -1.89 | 0.4242 |
| Achn062691 | Achn062691 | 2.62 | 0.03 | 0.42 | -6.52 | 0.0124 | -2.63 | 0.4242 |
| Achn064001 | Achn064001 | 0.37 | 24.93 | 0.70 | 6.07 | 0.0004 | 0.92 | 0.8290 |
| Achn067551 | Achn067551 | 13.17 | 200.37 | 117.57 | 3.93 | 0.0170 | 3.16 | 0.1428 |
| Achn067571 | Achn067571 | 1.20 | 52.28 | 5.32 | 5.45 | 0.0005 | 2.15 | 0.4242 |
| Achn068281 | Achn068281 | 0.03 | 1.69 | 0.39 | 6.04 | 0.0336 | 3.91 | 1.0000 |
| Achn072671 | Achn072671 | 0.31 | 24.66 | 5.77 | 6.31 | 0.0001 | 4.21 | 0.0775 |
| Achn075401 | Achn075401 | 0.18 | 9.25 | 0.11 | 5.68 | 0.0032 | -0.68 | 1.0000 |
| Achn077101 | Achn077101 | 10.22 | 112.65 | 60.60 | 3.46 | 0.0284 | 2.57 | 0.2905 |
| Achn077451 | Achn077451 | 0.96 | 38.29 | 0.65 | 5.31 | 0.0032 | -0.57 | 0.9184 |
| Achn077781 | Achn077781 | 3.21 | 44.80 | 4.81 | 3.80 | 0.0125 | 0.58 | 0.8558 |
| Achn078941 | Achn078941 | 0.15 | 13.32 | 0.92 | 6.44 | 0.0057 | 2.58 | 0.4242 |
| Achn081471 | Achn081471 | 65.48 | 868.00 | 289.50 | 3.73 | 0.0202 | 2.14 | 0.4242 |
| Achn082221 | Achn082221 | 1.40 | 22.95 | 2.31 | 4.03 | 0.0399 | 0.72 | 0.8557 |
| Achn083791 | Achn083791 | 192.56 | 5.43 | 28.46 | -5.15 | 0.0006 | -2.76 | 0.2279 |
| Achn090191 | Achn090191 | 69.30 | 864.62 | 193.97 | 3.64 | 0.0175 | 1.48 | 0.4242 |
| Achn091071 | Achn091071 | 0.51 | 25.67 | 1.67 | 5.65 | 0.0064 | 1.71 | 0.5614 |
| Achn093111 | Achn093111 | 1.45 | 32.46 | 0.33 | 4.49 | 0.0058 | -2.13 | 0.4242 |
| Achn094571 | Achn094571 | 3.11 | 99.89 | 17.96 | 5.00 | 0.0004 | 2.53 | 0.4242 |
| Achn095091 | Achn095091 | 1.62 | 27.73 | 5.24 | 4.10 | 0.0171 | 1.69 | 0.4242 |
| Achn097971 | Achn097971 | 1.45 | 328.87 | 3.79 | 7.83 | 0.0000 | 1.39 | 0.7280 |
| Achn102081 | Achn102081 | 2.21 | 31.29 | 6.41 | 3.82 | 0.0437 | 1.54 | 0.4623 |
| Achn105121 | Achn105121 | 0.41 | 19.64 | 1.10 | 5.59 | 0.0003 | 1.43 | 0.5642 |
| Achn106811 | Achn106811 | 3.49 | 192.76 | 0.21 | 5.79 | 0.0000 | -4.05 | 0.2277 |
| Achn107921 | Achn107921 | 110.41 | 1294.76 | 47.59 | 3.55 | 0.0197 | -1.21 | 0.5347 |
| Achn108051 | Achn108051 | 0.21 | 11.07 | 0.03 | 5.69 | 0.0021 | -2.90 | 1.0000 |
| Achn109291 | Achn109291 | 0.02 | 1.79 | 0.11 | 6.30 | 0.0201 | 2.23 | 1.0000 |
| Achn111051 | Achn111051 | 110.38 | 1389.85 | 317.28 | 3.65 | 0.0224 | 1.52 | 0.4242 |
| Achn114351 | Achn114351 | 2.20 | 75.07 | 2.26 | 5.09 | 0.0022 | 0.04 | 0.9974 |
| Achn115461 | Achn115461 | 2.23 | 86.48 | 0.44 | 5.28 | 0.0003 | -2.33 | 0.4242 |
| Achn115781 | Achn115781 | 0.45 | 14.25 | 0.43 | 4.98 | 0.0070 | -0.09 | 1.0000 |
| Achn116381 | Achn116381 | 20.40 | 0.75 | 19.48 | -4.77 | 0.0380 | -0.07 | 0.9923 |
| Achn116401 | Achn116401 | 0.26 | 20.69 | 0.94 | 6.30 | 0.0028 | 1.83 | 0.5513 |
| Achn116421 | Achn116421 | 0.49 | 13.55 | 0.83 | 4.79 | 0.0154 | 0.77 | 0.8642 |
| Achn116921 | Achn116921 | 0.52 | 37.06 | 0.32 | 6.16 | 0.0000 | -0.68 | 1.0000 |
| Achn117881 | Achn117881 | 4.39 | 0.05 | 0.38 | -6.57 | 0.0112 | -3.51 | 0.3144 |
| Achn123621 | Achn123621 | 0.09 | 6.35 | 0.78 | 6.10 | 0.0126 | 3.08 | 0.4242 |
| Achn126191 | Achn126191 | 8.41 | 152.72 | 34.35 | 4.18 | 0.0040 | 2.03 | 0.4242 |
| Achn128051 | Achn128051 | 6.62 | 80.58 | 6.91 | 3.61 | 0.0260 | 0.06 | 0.9930 |
| Achn129601 | Achn129601 | 0.04 | 4.48 | 0.15 | 6.82 | 0.0064 | 1.91 | 1.0000 |
| Achn129841 | Achn129841 | 2.37 | 41.06 | 25.80 | 4.11 | 0.0107 | 3.44 | 0.0769 |
| Achn129911 | Achn129911 | 33.26 | 550.19 | 67.62 | 4.05 | 0.0043 | 1.02 | 0.5737 |
| Achn130451 | Achn130451 | 21.91 | 1061.58 | 41.47 | 5.60 | 0.0000 | 0.92 | 0.6560 |
| Achn130531 | Achn130531 | 70.66 | 1324.89 | 50.13 | 4.23 | 0.0128 | -0.50 | 0.8652 |
| Achn131571 | Achn131571 | 0.15 | 7.34 | 0.05 | 5.58 | 0.0393 | -1.67 | 1.0000 |
| Achn131681 | Achn131681 | 24.46 | 0.30 | 0.00 | -6.37 | 0.0138 | -17.90 | 0.4242 |
| Achn132241 | Achn132241 | 44.30 | 447.09 | 26.78 | 3.34 | 0.0428 | -0.73 | 0.7578 |
| Achn134021 | Achn134021 | 0.83 | 52.79 | 2.61 | 5.98 | 0.0001 | 1.65 | 0.4751 |
| Achn135011 | Achn135011 | 1.54 | 31.86 | 3.14 | 4.37 | 0.0073 | 1.03 | 0.7142 |
| Achn137101 | Achn137101 | 5.50 | 0.04 | 23.36 | -7.00 | 0.0042 | 2.09 | 0.4242 |
| Achn138591 | Achn138591 | 0.58 | 46.18 | 5.83 | 6.32 | 0.0000 | 3.34 | 0.2965 |
| Achn140681 | Achn140681 | 2.77 | 57.37 | 2.42 | 4.37 | 0.0085 | -0.19 | 0.9750 |
| Achn143801 | Achn143801 | 30.99 | 691.95 | 16.36 | 4.48 | 0.0010 | -0.92 | 0.6986 |
| Achn144051 | Achn144051 | 1.40 | 43.44 | 2.03 | 4.96 | 0.0066 | 0.53 | 0.9163 |
| Achn144171 | Achn144171 | 0.51 | 22.60 | 12.02 | 5.47 | 0.0235 | 4.55 | 0.1432 |
| Achn148261 | Achn148261 | 0.98 | 16.01 | 0.86 | 4.03 | 0.0341 | -0.20 | 0.9749 |
| Achn150221 | Achn150221 | 3.19 | 42.51 | 5.85 | 3.74 | 0.0344 | 0.87 | 0.7649 |
| Achn150611 | Achn150611 | 4.41 | 426.30 | 20.42 | 6.60 | 0.0000 | 2.21 | 0.4242 |
| Achn150741 | Achn150741 | 0.42 | 11.05 | 1.63 | 4.72 | 0.0102 | 1.96 | 0.4242 |
| Achn152391 | Achn152391 | 27.82 | 312.93 | 10.92 | 3.49 | 0.0394 | -1.35 | 0.5332 |
| Achn161011 | Achn161011 | 16.82 | 199.69 | 4.25 | 3.57 | 0.0194 | -1.99 | 0.4242 |
| Achn161141 | Achn161141 | 31.39 | 571.13 | 59.40 | 4.19 | 0.0028 | 0.92 | 0.6417 |
| Achn161881 | Achn161881 | 10.45 | 0.30 | 10.69 | -5.14 | 0.0148 | 0.03 | 0.9970 |
| Achn162021 | Achn162021 | 1.22 | 19.80 | 0.25 | 4.03 | 0.0229 | -2.28 | 0.4242 |
| Achn163081 | Achn163081 | 1.57 | 23.25 | 1.85 | 3.89 | 0.0262 | 0.24 | 0.9651 |
| Achn163791 | Achn163791 | 0.09 | 16.82 | 0.38 | 7.49 | 0.0003 | 2.03 | 1.0000 |
| Achn165611 | Achn165611 | 35.69 | 1.62 | 19.20 | -4.46 | 0.0422 | -0.89 | 0.7329 |
| Achn169581 | Achn169581 | 8.44 | 112.92 | 3.75 | 3.74 | 0.0157 | -1.17 | 0.5984 |
| Achn169961 | Achn169961 | 6.76 | 257.60 | 29.48 | 5.25 | 0.0001 | 2.12 | 0.4242 |
| Achn171731 | Achn171731 | 2.46 | 86.35 | 50.65 | 5.13 | 0.0170 | 4.36 | 0.0975 |
| Achn172621 | Achn172621 | 2.20 | 80.08 | 3.14 | 5.19 | 0.0004 | 0.51 | 0.9035 |
| Achn173901 | Achn173901 | 1.20 | 64.15 | 3.02 | 5.74 | 0.0008 | 1.33 | 0.6585 |
| Achn174161 | Achn174161 | 10.43 | 0.09 | 0.72 | -6.83 | 0.0050 | -3.85 | 0.0874 |
| Achn176331 | Achn176331 | 0.84 | 44.24 | 0.41 | 5.72 | 0.0029 | -1.04 | 0.8522 |
| Achn177571 | Achn177571 | 2.67 | 120.19 | 3.93 | 5.49 | 0.0001 | 0.56 | 0.8936 |
| Achn181451 | Achn181451 | 0.07 | 5.13 | 0.02 | 6.20 | 0.0102 | -1.68 | 1.0000 |
| Achn181831 | Achn181831 | 19.28 | 0.59 | 83.52 | -5.04 | 0.0089 | 2.12 | 0.4242 |
| Achn181881 | Achn181881 | 1.00 | 22.53 | 5.11 | 4.50 | 0.0149 | 2.36 | 0.4242 |
| Achn182961 | Achn182961 | 14.05 | 158.78 | 30.83 | 3.50 | 0.0253 | 1.13 | 0.5394 |
| Achn182971 | Achn182971 | 23.43 | 317.48 | 65.40 | 3.76 | 0.0138 | 1.48 | 0.4242 |
| Achn183441 | Achn183441 | 0.13 | 16.95 | 1.94 | 7.01 | 0.0033 | 3.88 | 0.4242 |
| Achn185171 | Achn185171 | 15.03 | 1045.32 | 10.56 | 6.12 | 0.0000 | -0.51 | 0.8832 |
| Achn188241 | Achn188241 | 31.28 | 361.13 | 18.74 | 3.53 | 0.0212 | -0.74 | 0.7736 |
| Achn188921 | Achn188921 | 0.42 | 7.69 | 1.64 | 4.20 | 0.0249 | 1.97 | 0.4242 |
| Achn191451 | Achn191451 | 0.38 | 96.29 | 1.80 | 8.00 | 0.0000 | 2.26 | 0.4242 |
| Achn193321 | Achn193321 | 0.51 | 13.13 | 11.12 | 4.69 | 0.0306 | 4.45 | 0.0539 |
| Achn196931 | Achn196931 | 0.18 | 6.55 | 4.50 | 5.16 | 0.0143 | 4.62 | 0.0538 |
| Achn199111 | Achn199111 | 6.16 | 129.83 | 12.03 | 4.40 | 0.0035 | 0.97 | 0.7134 |
| Achn199771 | Achn199771 | 1.80 | 59.20 | 15.97 | 5.04 | 0.0008 | 3.15 | 0.2221 |
| Achn200591 | Achn200591 | 0.16 | 67.40 | 0.15 | 8.72 | 0.0000 | -0.09 | 1.0000 |
| Achn202551 | Achn202551 | 5.49 | 66.27 | 31.02 | 3.59 | 0.0248 | 2.50 | 0.3923 |
| Achn203501 | Achn203501 | 15.56 | 183.16 | 3.38 | 3.56 | 0.0229 | -2.20 | 0.4242 |
| Achn204141 | Achn204141 | 0.81 | 28.58 | 2.26 | 5.14 | 0.0011 | 1.48 | 0.5329 |
| Achn205721 | Achn205721 | 2.38 | 36.77 | 29.42 | 3.95 | 0.0324 | 3.63 | 0.0767 |
| Achn207521 | Achn207521 | 0.14 | 3.82 | 0.06 | 4.79 | 0.0278 | -1.32 | 1.0000 |
| Achn209091 | Achn209091 | 19.10 | 431.13 | 121.23 | 4.50 | 0.0010 | 2.67 | 0.2142 |
| Achn209831 | Achn209831 | 1.56 | 32.72 | 1.54 | 4.39 | 0.0084 | -0.02 | 0.9991 |
| Achn209841 | Achn209841 | 2.01 | 30.83 | 0.83 | 3.94 | 0.0273 | -1.27 | 0.6380 |
| Achn209851 | Achn209851 | 0.40 | 13.69 | 2.90 | 5.11 | 0.0074 | 2.87 | 0.4242 |
| Achn209971 | Achn209971 | 1.84 | 55.23 | 2.30 | 4.91 | 0.0010 | 0.32 | 0.9492 |
| Achn210351 | Achn210351 | 8.15 | 176.42 | 8.37 | 4.44 | 0.0015 | 0.04 | 0.9963 |
| Achn210381 | Achn210381 | 3.21 | 205.35 | 0.76 | 6.00 | 0.0000 | -2.07 | 0.4242 |
| Achn212251 | Achn212251 | 0.73 | 23.67 | 0.26 | 5.01 | 0.0128 | -1.48 | 0.7310 |
| Achn213731 | Achn213731 | 0.62 | 17.26 | 2.63 | 4.81 | 0.0070 | 2.10 | 0.4242 |
| Achn214751 | Achn214751 | 27.94 | 293.06 | 62.19 | 3.39 | 0.0317 | 1.15 | 0.5046 |
| Achn215261 | Achn215261 | 1.40 | 44.64 | 0.20 | 4.99 | 0.0174 | -2.82 | 0.4242 |
| Achn223211 | Achn223211 | 64.56 | 0.79 | 28.03 | -6.36 | 0.0003 | -1.20 | 0.5179 |
| Achn227461 | Achn227461 | 5.78 | 176.22 | 12.32 | 4.93 | 0.0006 | 1.09 | 0.6580 |
| Achn227711 | Achn227711 | 5.48 | 225.16 | 5.75 | 5.36 | 0.0001 | 0.07 | 0.9923 |
| Achn227861 | Achn227861 | 2.18 | 45.95 | 0.32 | 4.40 | 0.0044 | -2.77 | 0.4242 |
| Achn229131 | Achn229131 | 3.44 | 113.53 | 14.24 | 5.05 | 0.0003 | 2.05 | 0.4242 |
| Achn229681 | Achn229681 | 1.56 | 314.99 | 0.00 | 7.65 | 0.0000 | -13.93 | 0.4242 |
| Achn229731 | Achn229731 | 1.21 | 32.30 | 0.15 | 4.74 | 0.0272 | -3.03 | 0.4242 |
| Achn230311 | Achn230311 | 49.35 | 0.91 | 13.55 | -5.77 | 0.0001 | -1.86 | 0.4242 |
| Achn231551 | Achn231551 | 0.11 | 42.66 | 0.40 | 8.57 | 0.0000 | 1.82 | 1.0000 |
| Achn233691 | Achn233691 | 0.22 | 11.71 | 2.94 | 5.72 | 0.0124 | 3.73 | 0.4242 |
| Achn235381 | Achn235381 | 0.46 | 130.17 | 1.16 | 8.14 | 0.0002 | 1.33 | 0.7998 |
| Achn238921 | Achn238921 | 266.51 | 11.54 | 63.25 | -4.53 | 0.0009 | -2.07 | 0.4242 |
| Achn239001 | Achn239001 | 0.24 | 83.39 | 0.23 | 8.44 | 0.0001 | -0.05 | 1.0000 |
| Achn240201 | Achn240201 | 0.43 | 27.12 | 0.77 | 5.99 | 0.0002 | 0.85 | 0.8374 |
| Achn241381 | Achn241381 | 2.69 | 51.24 | 5.53 | 4.25 | 0.0048 | 1.04 | 0.6619 |
| Achn245401 | Achn245401 | 3.07 | 41.96 | 2.79 | 3.77 | 0.0389 | -0.13 | 0.9840 |
| Achn245941 | Achn245941 | 1.16 | 42.31 | 5.82 | 5.18 | 0.0013 | 2.32 | 0.4242 |
| Achn246151 | Achn246151 | 7.60 | 656.44 | 23.68 | 6.43 | 0.0000 | 1.64 | 0.4242 |
| Achn246161 | Achn246161 | 8.86 | 146.74 | 48.98 | 4.05 | 0.0051 | 2.47 | 0.3959 |
| Achn246401 | Achn246401 | 55.40 | 598.77 | 47.48 | 3.43 | 0.0308 | -0.22 | 0.9553 |
| Achn252801 | Achn252801 | 0.25 | 23.34 | 4.15 | 6.53 | 0.0101 | 4.04 | 0.4242 |
| Achn254871 | Achn254871 | 3.86 | 222.50 | 12.42 | 5.85 | 0.0000 | 1.69 | 0.4242 |
| Achn256121 | Achn256121 | 1.51 | 8387.90 | 0.88 | 12.44 | 0.0000 | -0.78 | 0.9212 |
| Achn256541 | Achn256541 | 3.55 | 86.23 | 17.55 | 4.60 | 0.0125 | 2.30 | 0.4242 |
| Achn256701 | Achn256701 | 4.97 | 82.54 | 23.16 | 4.05 | 0.0126 | 2.22 | 0.4242 |
| Achn257861 | Achn257861 | 3.30 | 145.34 | 0.13 | 5.46 | 0.0001 | -4.63 | 0.2082 |
| Achn259251 | Achn259251 | 17.23 | 321.16 | 60.79 | 4.22 | 0.0048 | 1.82 | 0.4242 |
| Achn260411 | Achn260411 | 3.78 | 172.18 | 2.65 | 5.51 | 0.0109 | -0.51 | 0.9460 |
| Achn260481 | Achn260481 | 0.70 | 16.03 | 0.39 | 4.52 | 0.0083 | -0.83 | 0.8348 |
| Achn261701 | Achn261701 | 0.12 | 16.53 | 3.77 | 7.08 | 0.0035 | 4.95 | 0.2015 |
| Achn266221 | Achn266221 | 0.73 | 20.32 | 1.55 | 4.80 | 0.0021 | 1.09 | 0.6921 |
| Achn266941 | Achn266941 | 34.25 | 360.91 | 36.18 | 3.40 | 0.0315 | 0.08 | 0.9891 |
| Achn269761 | Achn269761 | 0.55 | 114.36 | 0.78 | 7.71 | 0.0000 | 0.51 | 0.9279 |
| Achn273801 | Achn273801 | 1.60 | 26.41 | 1.56 | 4.05 | 0.0088 | -0.03 | 0.9972 |
| Achn277471 | Achn277471 | 0.39 | 40.81 | 5.10 | 6.70 | 0.0009 | 3.70 | 0.4242 |
| Achn278991 | Achn278991 | 3.58 | 85.38 | 16.57 | 4.58 | 0.0097 | 2.21 | 0.4242 |
| Achn279661 | Achn279661 | 12.74 | 138.38 | 12.48 | 3.44 | 0.0338 | -0.03 | 0.9973 |
| Achn280371 | Achn280371 | 0.15 | 13.54 | 2.30 | 6.51 | 0.0048 | 3.95 | 0.4242 |
| Achn285831 | Achn285831 | 0.12 | 6.82 | 0.11 | 5.87 | 0.0465 | -0.05 | 1.0000 |
| Achn286371 | Achn286371 | 0.10 | 206.48 | 2.15 | 11.05 | 0.0000 | 4.46 | 0.3768 |
| Achn286941 | Achn286941 | 2.25 | 28.59 | 3.65 | 3.67 | 0.0297 | 0.70 | 0.8259 |
| Achn288501 | Achn288501 | 86.33 | 1.10 | 357.62 | -6.30 | 0.0000 | 2.05 | 0.4242 |
| Achn289291 | Achn289291 | 58.04 | 1464.21 | 425.42 | 4.66 | 0.0008 | 2.87 | 0.1293 |
| Achn289681 | Achn289681 | 16.19 | 510.70 | 12.71 | 4.98 | 0.0005 | -0.35 | 0.9423 |
| Achn289691 | Achn289691 | 32.15 | 1138.30 | 28.02 | 5.15 | 0.0001 | -0.20 | 0.9690 |
| Achn290511 | Achn290511 | 0.15 | 9.68 | 0.00 | 5.97 | 0.0321 | -10.59 | 1.0000 |
| Achn290521 | Achn290521 | 0.37 | 12.72 | 0.00 | 5.11 | 0.0262 | -11.85 | 1.0000 |
| Achn292051 | Achn292051 | 1.18 | 31.02 | 0.70 | 4.72 | 0.0022 | -0.76 | 0.8398 |
| Achn293761 | Achn293761 | 2.12 | 46.72 | 0.55 | 4.46 | 0.0072 | -1.95 | 0.4242 |
| Achn296581 | Achn296581 | 0.71 | 21.94 | 1.02 | 4.95 | 0.0152 | 0.52 | 0.9258 |
| Achn297911 | Achn297911 | 1.12 | 34.43 | 0.22 | 4.95 | 0.0100 | -2.37 | 0.4588 |
| Achn298101 | Achn298101 | 0.70 | 18.96 | 0.56 | 4.76 | 0.0402 | -0.31 | 0.9668 |
| Achn298691 | Achn298691 | 0.06 | 4.09 | 0.39 | 6.05 | 0.0143 | 2.64 | 1.0000 |
| Achn300531 | Achn300531 | 4.13 | 87.83 | 7.48 | 4.41 | 0.0041 | 0.86 | 0.7744 |
| Achn301111 | Achn301111 | 36.80 | 1.37 | 164.39 | -4.74 | 0.0084 | 2.16 | 0.4242 |
| Achn301951 | Achn301951 | 0.07 | 4.60 | 0.20 | 6.07 | 0.0316 | 1.51 | 1.0000 |
| Achn302571 | Achn302571 | 1.87 | 41.41 | 0.59 | 4.47 | 0.0026 | -1.66 | 0.4244 |
| Achn302941 | Achn302941 | 193.08 | 7.98 | 195.34 | -4.60 | 0.0007 | 0.02 | 0.9984 |
| Achn303421 | Achn303421 | 4.53 | 0.04 | 0.39 | -6.87 | 0.0057 | -3.52 | 0.2620 |
| Achn304011 | Achn304011 | 9.52 | 144.50 | 32.34 | 3.92 | 0.0087 | 1.76 | 0.4242 |
| Achn305061 | Achn305061 | 0.33 | 9.75 | 0.94 | 4.88 | 0.0298 | 1.51 | 0.6260 |
| Achn306671 | Achn306671 | 55.11 | 691.98 | 450.95 | 3.65 | 0.0144 | 3.03 | 0.0880 |
| Achn308371 | Achn308371 | 1.31 | 34.27 | 0.86 | 4.71 | 0.0067 | -0.62 | 0.8962 |
| Achn311801 | Achn311801 | 1.14 | 172.20 | 11.51 | 7.24 | 0.0000 | 3.34 | 0.3779 |
| Achn314441 | Achn314441 | 2.16 | 0.04 | 0.10 | -5.91 | 0.0425 | -4.43 | 0.2739 |
| Achn315711 | Achn315711 | 8.10 | 154.65 | 51.44 | 4.25 | 0.0043 | 2.67 | 0.3534 |
| Achn319051 | Achn319051 | 2.51 | 93.19 | 0.91 | 5.22 | 0.0002 | -1.45 | 0.5436 |
| Achn322221 | Achn322221 | 0.94 | 551.38 | 10.23 | 9.20 | 0.0000 | 3.44 | 0.2016 |
| Achn323261 | Achn323261 | 9.36 | 109.84 | 89.12 | 3.55 | 0.0279 | 3.25 | 0.0673 |
| Achn328421 | Achn328421 | 1.97 | 67.39 | 4.47 | 5.10 | 0.0012 | 1.18 | 0.6683 |
| Achn329911 | Achn329911 | 0.05 | 3.75 | 0.21 | 6.37 | 0.0174 | 2.24 | 1.0000 |
| Achn332071 | Achn332071 | 20.03 | 232.41 | 208.36 | 3.54 | 0.0329 | 3.38 | 0.0523 |
| Achn332421 | Achn332421 | 25.78 | 443.27 | 3.62 | 4.10 | 0.0034 | -2.83 | 0.3170 |
| Achn333401 | Achn333401 | 0.41 | 13.03 | 0.54 | 4.98 | 0.0052 | 0.38 | 0.9468 |
| Achn335471 | Achn335471 | 0.48 | 25.19 | 2.04 | 5.71 | 0.0303 | 2.09 | 0.5160 |
| Achn336161 | Achn336161 | 3.03 | 649.10 | 80.14 | 7.74 | 0.0006 | 4.72 | 0.2599 |
| Achn338861 | Achn338861 | 4.34 | 117.32 | 6.98 | 4.76 | 0.0011 | 0.68 | 0.8426 |
| Achn340271 | Achn340271 | 9.07 | 109.46 | 2.74 | 3.59 | 0.0201 | -1.73 | 0.4242 |
| Achn345331 | Achn345331 | 2.81 | 60.17 | 28.33 | 4.42 | 0.0212 | 3.34 | 0.2717 |
| Achn345471 | Achn345471 | 4.08 | 0.17 | 0.57 | -4.58 | 0.0395 | -2.83 | 0.4242 |
| Achn346401 | Achn346401 | 6.35 | 103.90 | 11.22 | 4.03 | 0.0089 | 0.82 | 0.7695 |
| Achn346471 | Achn346471 | 0.27 | 21.90 | 5.50 | 6.35 | 0.0072 | 4.35 | 0.3014 |
| Achn346941 | Achn346941 | 1.40 | 28.46 | 1.87 | 4.35 | 0.0095 | 0.42 | 0.9302 |
| Achn347351 | Achn347351 | 133.55 | 2814.00 | 321.31 | 4.40 | 0.0189 | 1.27 | 0.6386 |
| Achn351111 | Achn351111 | 0.03 | 8.14 | 0.16 | 7.94 | 0.0004 | 2.23 | 1.0000 |
| Achn351261 | Achn351261 | 13.79 | 186.01 | 27.80 | 3.75 | 0.0134 | 1.01 | 0.6283 |
| Achn351321 | Achn351321 | 5.56 | 141.35 | 2.46 | 4.67 | 0.0006 | -1.18 | 0.5878 |
| Achn351451 | Achn351451 | 1.39 | 20.07 | 3.54 | 3.85 | 0.0430 | 1.34 | 0.5578 |
| Achn351641 | Achn351641 | 0.95 | 20.33 | 0.57 | 4.42 | 0.0082 | -0.74 | 0.8527 |
| Achn352601 | Achn352601 | 14.98 | 381.29 | 23.38 | 4.67 | 0.0005 | 0.64 | 0.8112 |
| Achn355971 | Achn355971 | 8.15 | 118.29 | 20.94 | 3.86 | 0.0344 | 1.36 | 0.5448 |
| Achn357061 | Achn357061 | 26.04 | 244.53 | 131.17 | 3.23 | 0.0496 | 2.33 | 0.4183 |
| Achn357651 | Achn357651 | 2.70 | 37.68 | 16.67 | 3.80 | 0.0321 | 2.63 | 0.4242 |
| Achn357921 | Achn357921 | 0.14 | 2.90 | 0.66 | 4.37 | 0.0405 | 2.22 | 0.4242 |
| Achn359441 | Achn359441 | 8.35 | 0.06 | 19.99 | -7.24 | 0.0018 | 1.26 | 0.4630 |
| Achn361271 | Achn361271 | 0.11 | 7.20 | 1.90 | 6.04 | 0.0339 | 4.12 | 0.4242 |
| Achn363671 | Achn363671 | 1.07 | 38.50 | 0.28 | 5.18 | 0.0039 | -1.93 | 0.5635 |
| Achn364321 | Achn364321 | 13.83 | 162.55 | 2.56 | 3.55 | 0.0236 | -2.44 | 0.4242 |
| Achn366321 | Achn366321 | 55.19 | 727.28 | 25.46 | 3.72 | 0.0121 | -1.12 | 0.5471 |
| Achn366631 | Achn366631 | 0.31 | 6.49 | 0.04 | 4.40 | 0.0297 | -2.79 | 1.0000 |
| Achn369361 | Achn369361 | 13.48 | 236.85 | 26.97 | 4.14 | 0.0036 | 1.00 | 0.6308 |
| Achn370261 | Achn370261 | 1.61 | 20.55 | 1.13 | 3.68 | 0.0432 | -0.51 | 0.9051 |
| Achn371471 | Achn371471 | 27.19 | 786.76 | 26.55 | 4.85 | 0.0003 | -0.03 | 0.9964 |
| Achn374191 | Achn374191 | 73.61 | 2.36 | 186.06 | -4.97 | 0.0020 | 1.34 | 0.4242 |
| Achn374421 | Achn374421 | 4.09 | 49.70 | 11.89 | 3.60 | 0.0418 | 1.54 | 0.4242 |
| Achn375011 | Achn375011 | 29.27 | 330.19 | 82.66 | 3.50 | 0.0280 | 1.50 | 0.4242 |
| Achn378471 | Achn378471 | 5.46 | 0.07 | 4.31 | -6.20 | 0.0243 | -0.34 | 0.9430 |
| Achn378651 | Achn378651 | 8.38 | 170.56 | 63.52 | 4.35 | 0.0022 | 2.92 | 0.1698 |
| Achn379971 | Achn379971 | 0.83 | 15.67 | 1.65 | 4.24 | 0.0313 | 1.00 | 0.7654 |
| Achn380481 | Achn380481 | 0.63 | 24.81 | 0.34 | 5.30 | 0.0070 | -0.87 | 0.8750 |
| Achn380691 | Achn380691 | 1.28 | 25.72 | 1.10 | 4.33 | 0.0204 | -0.23 | 0.9715 |
| Achn380931 | Achn380931 | 1.50 | 27.20 | 1.87 | 4.18 | 0.0065 | 0.31 | 0.9465 |
| Achn383461 | Achn383461 | 8.87 | 89.54 | 24.05 | 3.34 | 0.0373 | 1.44 | 0.4242 |
| Achn383841 | Achn383841 | 11.03 | 191.84 | 70.23 | 4.12 | 0.0033 | 2.67 | 0.2150 |
| Achn385661 | Achn385661 | 35.05 | 1.83 | 26.49 | -4.26 | 0.0243 | -0.40 | 0.9095 |
| Achn387971 | Achn387971 | 0.02 | 12.71 | 0.11 | 9.02 | 0.0000 | 2.23 | 1.0000 |
| Achn313551 | Achn313551 | 0.10 | 54.37 | 364.95 | 9.11 | 0.0000 | 11.86 | 0.0000 |
| Achn069511 | Achn069511 | 0.12 | 6.55 | 116.06 | 5.72 | 0.0294 | 9.86 | 0.0000 |
| Achn342471 | Achn342471 | 0.12 | 21.15 | 57.08 | 7.47 | 0.0014 | 8.90 | 0.0000 |
| Achn260601 | Achn260601 | 0.38 | 154.40 | 100.62 | 8.66 | 0.0000 | 8.04 | 0.0000 |
| Achn086221 | Achn086221 | 71.63 | 11541.30 | 18719.90 | 7.33 | 0.0000 | 8.03 | 0.0000 |
| Achn227791 | Achn227791 | 0.20 | 18.85 | 41.61 | 6.57 | 0.0091 | 7.72 | 0.0005 |
| Achn154461 | Achn154461 | 0.04 | 7.99 | 8.43 | 7.61 | 0.0009 | 7.69 | 0.0007 |
| Achn085041 | Achn085041 | 0.13 | 90.87 | 21.15 | 9.49 | 0.0000 | 7.39 | 0.0005 |
| Achn119071 | Achn119071 | 0.09 | 12.87 | 13.00 | 7.11 | 0.0032 | 7.13 | 0.0030 |
| Achn142621 | Achn142621 | 0.60 | 24.45 | 79.53 | 5.35 | 0.0004 | 7.05 | 0.0000 |
| Achn159091 | Achn159091 | 0.02 | 2.07 | 2.41 | 6.65 | 0.0094 | 6.87 | 0.0056 |
| Achn092641 | Achn092641 | 0.23 | 8.81 | 26.00 | 5.24 | 0.0025 | 6.80 | 0.0000 |
| Achn260671 | Achn260671 | 0.13 | 10.23 | 13.97 | 6.33 | 0.0025 | 6.78 | 0.0007 |
| Achn111311 | Achn111311 | 0.03 | 2.45 | 3.15 | 6.36 | 0.0177 | 6.72 | 0.0079 |
| Achn100671 | Achn100671 | 0.30 | 153.64 | 24.73 | 9.01 | 0.0000 | 6.37 | 0.0000 |
| Achn269061 | Achn269061 | 14.50 | 170.37 | 1051.22 | 3.55 | 0.0219 | 6.18 | 0.0000 |
| Achn126261 | Achn126261 | 0.23 | 38.15 | 16.87 | 7.35 | 0.0019 | 6.18 | 0.0256 |
| Achn345841 | Achn345841 | 0.03 | 6.73 | 2.16 | 7.80 | 0.0005 | 6.16 | 0.0260 |
| Achn362941 | Achn362941 | 8.12 | 194.24 | 534.46 | 4.58 | 0.0021 | 6.04 | 0.0000 |
| Achn361021 | Achn361021 | 0.44 | 124.60 | 28.28 | 8.15 | 0.0002 | 6.01 | 0.0296 |
| Achn171281 | Achn171281 | 0.70 | 75.32 | 42.08 | 6.75 | 0.0085 | 5.91 | 0.0442 |
| Achn130551 | Achn130551 | 11.65 | 231.40 | 637.88 | 4.31 | 0.0052 | 5.77 | 0.0000 |
| Achn328811 | Achn328811 | 4.82 | 155.12 | 261.67 | 5.01 | 0.0011 | 5.76 | 0.0001 |
| Achn359661 | Achn359661 | 0.77 | 87.64 | 41.02 | 6.84 | 0.0001 | 5.74 | 0.0026 |
| Achn060311 | Achn060311 | 0.64 | 15.72 | 34.11 | 4.61 | 0.0144 | 5.73 | 0.0003 |
| Achn292671 | Achn292671 | 9.81 | 0.22 | 492.81 | -5.47 | 0.0228 | 5.65 | 0.0000 |
| Achn304301 | Achn304301 | 6.65 | 454.18 | 334.08 | 6.09 | 0.0000 | 5.65 | 0.0002 |
| Achn131421 | Achn131421 | 2.23 | 36.83 | 109.41 | 4.04 | 0.0259 | 5.61 | 0.0001 |
| Achn315151 | Achn315151 | 0.61 | 72.45 | 27.48 | 6.89 | 0.0000 | 5.49 | 0.0004 |
| Achn191341 | Achn191341 | 0.19 | 17.11 | 6.84 | 6.49 | 0.0002 | 5.17 | 0.0135 |
| Achn281461 | Achn281461 | 1.29 | 311.83 | 42.53 | 7.92 | 0.0000 | 5.05 | 0.0012 |
| Achn169421 | Achn169421 | 1.05 | 518.30 | 33.36 | 8.95 | 0.0000 | 4.99 | 0.0057 |
| Achn209941 | Achn209941 | 17.89 | 225.03 | 560.87 | 3.65 | 0.0178 | 4.97 | 0.0002 |
| Achn325961 | Achn325961 | 3.00 | 306.48 | 89.09 | 6.68 | 0.0000 | 4.89 | 0.0019 |
| Achn353821 | Achn353821 | 8.73 | 118.26 | 241.69 | 3.76 | 0.0256 | 4.79 | 0.0008 |
| Achn141771 | Achn141771 | 10.16 | 155.66 | 270.29 | 3.94 | 0.0080 | 4.73 | 0.0005 |
| Achn276401 | Achn276401 | 0.20 | 10.21 | 5.31 | 5.65 | 0.0035 | 4.71 | 0.0435 |
| Achn294421 | Achn294421 | 2.84 | 40.28 | 63.16 | 3.83 | 0.0228 | 4.47 | 0.0026 |
| Achn378601 | Achn378601 | 66.71 | 3421.91 | 1388.42 | 5.68 | 0.0001 | 4.38 | 0.0142 |
| Achn054961 | Achn054961 | 3.13 | 158.65 | 64.32 | 5.66 | 0.0001 | 4.36 | 0.0069 |
| Achn020601 | Achn020601 | 1.79 | 31.04 | 33.36 | 4.12 | 0.0177 | 4.22 | 0.0125 |
| Achn341891 | Achn341891 | 1.53 | 73.88 | 26.97 | 5.60 | 0.0005 | 4.14 | 0.0475 |
| Achn374391 | Achn374391 | 1.94 | 34.33 | 29.99 | 4.14 | 0.0138 | 3.95 | 0.0248 |
| Achn366681 | Achn366681 | 67.32 | 817.61 | 994.83 | 3.60 | 0.0170 | 3.89 | 0.0072 |
| Achn182861 | Achn182861 | 21.35 | 236.65 | 311.44 | 3.47 | 0.0321 | 3.87 | 0.0094 |
| Achn015131 | Achn015131 | 1.94 | 24.01 | 27.14 | 3.63 | 0.0289 | 3.80 | 0.0168 |
| Achn026311 | Achn026311 | 5.44 | 104.17 | 72.32 | 4.26 | 0.0033 | 3.73 | 0.0192 |
| Achn143751 | Achn143751 | 40.74 | 678.36 | 525.83 | 4.06 | 0.0048 | 3.69 | 0.0150 |
| Achn223051 | Achn223051 | 33.48 | 606.08 | 380.45 | 4.18 | 0.0031 | 3.51 | 0.0244 |
| Achn314301 | Achn314301 | 7.28 | 113.82 | 74.68 | 3.97 | 0.0065 | 3.36 | 0.0442 |
| Achn123601 | Achn123601 | 12.12 | 0.37 | 0.86 | -5.01 | 0.0036 | -3.82 | 0.0467 |
| Achn084101 | Achn084101 | 295.56 | 23.68 | 20.27 | -3.64 | 0.0191 | -3.87 | 0.0094 |
| Achn141711 | Achn141711 | 6611.88 | 404.45 | 410.23 | -4.03 | 0.0331 | -4.01 | 0.0356 |
| Achn203261 | Achn203261 | 91.74 | 1.37 | 5.52 | -6.07 | 0.0000 | -4.05 | 0.0090 |
| Achn095551 | Achn095551 | 48.39 | 1709.33 | 2.78 | 5.14 | 0.0001 | -4.12 | 0.0227 |
| Achn012091 | Achn012091 | 95.69 | 7.75 | 5.16 | -3.63 | 0.0245 | -4.21 | 0.0046 |
| Achn282341 | Achn282341 | 11.69 | 379.14 | 0.57 | 5.02 | 0.0002 | -4.35 | 0.0377 |
| Achn124951 | Achn124951 | 45.98 | 2.35 | 1.99 | -4.29 | 0.0113 | -4.53 | 0.0057 |
| Achn347891 | Achn347891 | 279.56 | 28.75 | 10.89 | -3.28 | 0.0498 | -4.68 | 0.0008 |
| Achn105301 | Achn105301 | 54.25 | 1.54 | 1.57 | -5.14 | 0.0015 | -5.11 | 0.0014 |
| Achn289831 | Achn289831 | 11.42 | 0.32 | 0.29 | -5.17 | 0.0070 | -5.30 | 0.0049 |
| Achn140301 | Achn140301 | 4.63 | 146.73 | 0.06 | 4.99 | 0.0005 | -6.20 | 0.0245 |
| Achn372361 | Achn372361 | 255.99 | 19.25 | 3.47 | -3.73 | 0.0120 | -6.21 | 0.0000 |
| Achn017741 | Achn017741 | 12.20 | 0.13 | 0.08 | -6.51 | 0.0048 | -7.20 | 0.0021 |
| Novel_000139 | XLOC_003340 | 0.25 | 15.06 | 0.49 | 5.93 | 0.0102 | 0.98 | 1.0000 |
| Novel_000202 | XLOC_005156 | 21.18 | 0.10 | 19.39 | -7.68 | 0.0435 | -0.13 | 0.9816 |
| Novel_000223 | XLOC_005681 | 0.16 | 6.25 | 0.00 | 5.32 | 0.0329 | -10.61 | 1.0000 |
| Novel_000487 | XLOC_014501 | 70.73 | 1.22 | 39.33 | -5.86 | 0.0001 | -0.85 | 0.6926 |
| Novel_000667 | XLOC_019366 | 1.17 | 22.08 | 6.04 | 4.24 | 0.0388 | 2.37 | 0.4242 |
| Novel_000723 | XLOC_020768 | 0.55 | 17.19 | 6.19 | 4.97 | 0.0042 | 3.50 | 0.2082 |
| Novel_000942 | XLOC_027646 | 0.48 | 32.75 | 1.36 | 6.10 | 0.0002 | 1.50 | 0.5783 |
| Novel_001096 | XLOC_030747 | 0.01 | 7.67 | 1.07 | 9.37 | 0.0040 | 6.53 | 0.2024 |
| Novel_001160 | XLOC_032166 | 0.65 | 19.33 | 0.80 | 4.89 | 0.0323 | 0.30 | 0.9654 |
| Novel_001766 | XLOC_049154 | 0.82 | 42.72 | 1.53 | 5.71 | 0.0098 | 0.91 | 0.8618 |
| Novel_001779 | XLOC_049705 | 182.23 | 13.84 | 63.71 | -3.72 | 0.0365 | -1.52 | 0.4242 |
| Novel_001839 | XLOC_051159 | 33.48 | 1.30 | 209.77 | -4.68 | 0.0038 | 2.65 | 0.2185 |
| Novel_001995 | XLOC_054842 | 0.51 | 13.19 | 1.01 | 4.70 | 0.0317 | 1.00 | 0.8228 |
| Novel_002149 | XLOC_059169 | 3.18 | 53.54 | 0.54 | 4.07 | 0.0051 | -2.56 | 0.4242 |
| Novel_002163 | XLOC_059527 | 61.58 | 1.12 | 29.80 | -5.78 | 0.0245 | -1.05 | 0.6627 |
| Novel_002249 | XLOC_062900 | 12.58 | 197.38 | 54.05 | 3.97 | 0.0244 | 2.10 | 0.4242 |
| Novel_002335 | XLOC_064445 | 0.12 | 12.07 | 0.19 | 6.65 | 0.0164 | 0.68 | 1.0000 |
| Novel_002336 | XLOC_064445 | 0.27 | 45.64 | 0.00 | 7.40 | 0.0004 | -11.40 | 1.0000 |
| Novel_002778 | XLOC_074062 | 0.22 | 6.55 | 1.22 | 4.89 | 0.0438 | 2.47 | 0.4242 |
| Novel_002960 | XLOC_078304 | 1.08 | 25.48 | 0.27 | 4.57 | 0.0057 | -1.99 | 0.4242 |
| Novel_002996 | XLOC_079879 | 0.07 | 99.63 | 0.15 | 10.41 | 0.0000 | 0.98 | 1.0000 |
| Novel_002997 | XLOC_079879 | 29.56 | 459.57 | 10.84 | 3.96 | 0.0238 | -1.45 | 0.4242 |
| Novel_003241 | XLOC_083493 | 218.58 | 1.19 | 395.31 | -7.52 | 0.0000 | 0.85 | 0.6875 |
| Novel_003329 | XLOC_086227 | 219.94 | 15.38 | 32.82 | -3.84 | 0.0158 | -2.74 | 0.2027 |
| Novel_003369 | XLOC_087550 | 20.86 | 0.10 | 36.51 | -7.69 | 0.0435 | 0.81 | 0.7494 |
| Novel_003386 | XLOC_088171 | 0.08 | 2.69 | 0.30 | 5.04 | 0.0248 | 1.85 | 1.0000 |
| Novel_003407 | XLOC_088596 | 1.98 | 0.05 | 0.67 | -5.19 | 0.0423 | -1.55 | 0.4242 |
| Novel_003438 | XLOC_089442 | 0.19 | 6.61 | 0.29 | 5.14 | 0.0170 | 0.64 | 1.0000 |
| Novel_003467 | XLOC_090377 | 0.36 | 17.44 | 0.95 | 5.59 | 0.0174 | 1.40 | 0.7219 |
| Novel_003595 | XLOC_093394 | 0.88 | 56.56 | 1.12 | 6.00 | 0.0002 | 0.34 | 0.9537 |
| Novel_003627 | XLOC_093766 | 2.09 | 47.78 | 3.18 | 4.51 | 0.0056 | 0.60 | 0.8766 |
| Novel_003693 | XLOC_095221 | 1.26 | 35.68 | 1.52 | 4.83 | 0.0206 | 0.27 | 0.9688 |
| Novel_003799 | XLOC_097749 | 24.62 | 1174.48 | 56.21 | 5.58 | 0.0000 | 1.19 | 0.5176 |
| Novel_004166 | XLOC_105797 | 0.99 | 396.52 | 0.00 | 8.65 | 0.0000 | -13.27 | 0.4242 |
| Novel_004167 | XLOC_105797 | 5.03 | 325.15 | 0.00 | 6.01 | 0.0014 | -15.62 | 0.4242 |
| Novel_004272 | XLOC_107048 | 58.69 | 674.39 | 18.93 | 3.52 | 0.0228 | -1.63 | 0.4242 |
| Novel_004329 | XLOC_108102 | 16.45 | 178.29 | 15.71 | 3.44 | 0.0292 | -0.07 | 0.9913 |
| Novel_004337 | XLOC_108476 | 0.10 | 9.95 | 1.73 | 6.60 | 0.0214 | 4.07 | 0.4242 |
| Novel_004342 | XLOC_108492 | 0.89 | 18.56 | 1.07 | 4.38 | 0.0103 | 0.27 | 0.9598 |
| Novel_004390 | XLOC_110703 | 0.31 | 10.04 | 0.00 | 5.00 | 0.0380 | -11.61 | 1.0000 |
| Novel_004393 | XLOC_110825 | 26.76 | 0.36 | 4.52 | -6.21 | 0.0001 | -2.57 | 0.3702 |
| Novel_004406 | XLOC_111005 | 0.27 | 6.89 | 0.13 | 4.70 | 0.0457 | -1.08 | 1.0000 |
| Novel_004522 | XLOC_113683 | 3.28 | 67.63 | 5.84 | 4.36 | 0.0348 | 0.83 | 0.8373 |
| Novel_004565 | XLOC_115278 | 2.26 | 63.18 | 4.18 | 4.80 | 0.0015 | 0.89 | 0.7740 |
| Novel_004695 | XLOC_119976 | 6.73 | 78.71 | 8.96 | 3.55 | 0.0494 | 0.41 | 0.9218 |
| Novel_004841 | XLOC_124135 | 142.28 | 1.80 | 158.18 | -6.31 | 0.0072 | 0.15 | 0.9752 |
| Novel_004845 | XLOC_124184 | 113.39 | 5.41 | 33.30 | -4.39 | 0.0075 | -1.77 | 0.4242 |
| Novel_004926 | XLOC_126861 | 0.36 | 13.21 | 0.37 | 5.19 | 0.0154 | 0.04 | 1.0000 |
| Novel_004950 | XLOC_127424 | 0.72 | 14.81 | 4.14 | 4.37 | 0.0127 | 2.53 | 0.4242 |
| Novel_005103 | XLOC_130776 | 0.56 | 9.62 | 2.47 | 4.10 | 0.0394 | 2.14 | 0.4242 |
| Novel_005104 | XLOC_130776 | 0.81 | 19.16 | 1.02 | 4.56 | 0.0140 | 0.32 | 0.9579 |
| Novel_005139 | XLOC_131175 | 17.05 | 277.84 | 18.07 | 4.03 | 0.0045 | 0.08 | 0.9879 |
| Novel_005140 | XLOC_131206 | 178.83 | 1.06 | 249.74 | -7.40 | 0.0000 | 0.48 | 0.8770 |
| Novel_005398 | XLOC_138584 | 22.45 | 0.19 | 10.40 | -6.86 | 0.0046 | -1.11 | 0.6140 |
| Novel_005673 | XLOC_142441 | 9.87 | 128.04 | 11.47 | 3.70 | 0.0453 | 0.22 | 0.9668 |
| Novel_005792 | XLOC_144731 | 0.73 | 173.87 | 13.32 | 7.89 | 0.0000 | 4.19 | 0.1973 |
| Novel_005794 | XLOC_144731 | 0.22 | 730.82 | 0.61 | 11.70 | 0.0000 | 1.48 | 0.8370 |
| Novel_006020 | XLOC_152034 | 15.96 | 0.70 | 3.09 | -4.50 | 0.0386 | -2.37 | 0.4242 |
| Novel_006096 | XLOC_154016 | 0.33 | 12.31 | 5.48 | 5.22 | 0.0070 | 4.05 | 0.1276 |
| Novel_006622 | XLOC_170454 | 9.15 | 0.13 | 10.17 | -6.19 | 0.0205 | 0.15 | 0.9773 |
| Novel_006651 | XLOC_171362 | 0.13 | 34.50 | 0.78 | 8.01 | 0.0003 | 2.54 | 0.4293 |
| Novel_006757 | XLOC_173849 | 74.96 | 0.24 | 11.33 | -8.26 | 0.0188 | -2.73 | 0.4242 |
| Novel_006850 | XLOC_175830 | 0.09 | 5.88 | 0.04 | 5.96 | 0.0445 | -1.15 | 1.0000 |
| Novel_006895 | XLOC_176878 | 32.09 | 1.78 | 49.08 | -4.17 | 0.0169 | 0.61 | 0.8172 |
| Novel_006898 | XLOC_177102 | 9.55 | 106.62 | 24.36 | 3.48 | 0.0467 | 1.35 | 0.4733 |
| Novel_007148 | XLOC_183043 | 0.19 | 7.10 | 0.48 | 5.26 | 0.0366 | 1.38 | 1.0000 |
| Novel_007155 | XLOC_183269 | 65.29 | 0.20 | 116.56 | -8.36 | 0.0000 | 0.84 | 0.7099 |
| Novel_007156 | XLOC_183381 | 26.78 | 0.91 | 7.23 | -4.88 | 0.0284 | -1.89 | 0.4242 |
| Novel_007157 | XLOC_183382 | 36.78 | 1.05 | 6.45 | -5.13 | 0.0151 | -2.51 | 0.4242 |
| Novel_007504 | XLOC_191890 | 5.03 | 84.14 | 0.36 | 4.06 | 0.0265 | -3.80 | 0.3189 |
| Novel_007518 | XLOC_192811 | 0.35 | 39.13 | 5.39 | 6.82 | 0.0006 | 3.96 | 0.3573 |
| Novel_007558 | XLOC_195102 | 0.24 | 42.37 | 0.28 | 7.45 | 0.0001 | 0.19 | 1.0000 |
| Novel_007590 | XLOC_196610 | 268.17 | 16.50 | 282.12 | -4.02 | 0.0342 | 0.07 | 0.9899 |
| Novel_008079 | XLOC_210803 | 0.14 | 13.81 | 0.10 | 6.63 | 0.0063 | -0.54 | 1.0000 |
| Novel_008080 | XLOC_210816 | 0.73 | 27.68 | 0.00 | 5.25 | 0.0016 | -12.83 | 0.4242 |
| Novel_008121 | XLOC_212033 | 0.15 | 4.69 | 1.66 | 4.97 | 0.0189 | 3.47 | 0.3869 |
| Novel_008299 | XLOC_217127 | 0.07 | 3.96 | 0.40 | 5.83 | 0.0497 | 2.51 | 1.0000 |
| Novel_008632 | XLOC_225501 | 0.62 | 18.35 | 0.25 | 4.88 | 0.0265 | -1.34 | 0.8036 |
| Novel_008690 | XLOC_226979 | 1.28 | 30.14 | 3.36 | 4.56 | 0.0190 | 1.39 | 0.6318 |
| Novel_008851 | XLOC_232200 | 68.77 | 799.54 | 406.87 | 3.54 | 0.0414 | 2.56 | 0.3256 |
| Novel_008879 | XLOC_233516 | 0.30 | 17.38 | 2.96 | 5.86 | 0.0109 | 3.31 | 0.4242 |
| Novel_008952 | XLOC_234298 | 41.45 | 399.79 | 8.57 | 3.27 | 0.0491 | -2.27 | 0.4242 |
| Novel_009343 | XLOC_243716 | 3.47 | 61.56 | 31.96 | 4.15 | 0.0068 | 3.20 | 0.1120 |
| Novel_009374 | XLOC_244460 | 66.58 | 900.85 | 198.03 | 3.76 | 0.0134 | 1.57 | 0.4242 |
| Novel_009375 | XLOC_244460 | 8.98 | 143.95 | 27.76 | 4.00 | 0.0299 | 1.63 | 0.4242 |
| Novel_009622 | XLOC_250317 | 47.02 | 909.81 | 121.02 | 4.27 | 0.0019 | 1.36 | 0.4242 |
| Novel_009841 | XLOC_256882 | 0.08 | 6.91 | 0.24 | 6.35 | 0.0004 | 1.51 | 1.0000 |
| Novel_009842 | XLOC_256882 | 0.13 | 29.99 | 0.31 | 7.86 | 0.0002 | 1.27 | 1.0000 |
| Novel_009879 | XLOC_257965 | 1.00 | 88.97 | 20.08 | 6.47 | 0.0056 | 4.33 | 0.3150 |
| Novel_009880 | XLOC_257969 | 0.02 | 1.48 | 0.22 | 5.99 | 0.0499 | 3.25 | 1.0000 |
| Novel_009930 | XLOC_258981 | 33.31 | 0.32 | 241.89 | -6.69 | 0.0082 | 2.86 | 0.2292 |
| Novel_010037 | XLOC_262462 | 8.13 | 0.07 | 5.19 | -6.81 | 0.0006 | -0.65 | 0.8182 |
| Novel_010119 | XLOC_265065 | 0.29 | 18.37 | 8.82 | 5.98 | 0.0378 | 4.93 | 0.2266 |
| Novel_010309 | XLOC_270100 | 0.93 | 79.85 | 8.36 | 6.42 | 0.0001 | 3.16 | 0.4242 |
| Novel_010826 | XLOC_281652 | 185.99 | 9.89 | 160.45 | -4.23 | 0.0067 | -0.21 | 0.9572 |
| Novel_011252 | XLOC_290459 | 10.84 | 0.42 | 6.40 | -4.70 | 0.0021 | -0.76 | 0.7365 |
| Novel_011270 | XLOC_290750 | 20.26 | 0.79 | 8.18 | -4.69 | 0.0013 | -1.31 | 0.4242 |
| Novel_011308 | XLOC_291470 | 15.67 | 0.52 | 10.32 | -4.90 | 0.0015 | -0.60 | 0.8210 |
| Novel_011324 | XLOC_291596 | 1.56 | 41.23 | 0.00 | 4.73 | 0.0034 | -13.93 | 0.4242 |
| Novel_011355 | XLOC_292103 | 8.94 | 0.34 | 8.37 | -4.70 | 0.0085 | -0.09 | 0.9866 |
| Novel_011404 | XLOC_292974 | 51.80 | 0.34 | 55.06 | -7.25 | 0.0000 | 0.09 | 0.9867 |
| Novel_011415 | XLOC_293171 | 208.95 | 0.62 | 144.54 | -8.39 | 0.0000 | -0.53 | 0.8549 |
| Novel_011872 | XLOC_307039 | 0.33 | 8.51 | 1.34 | 4.68 | 0.0224 | 2.01 | 0.4242 |
| Novel_011880 | XLOC_307292 | 0.06 | 2.57 | 0.15 | 5.37 | 0.0283 | 1.23 | 1.0000 |
| Novel_011947 | XLOC_309053 | 1.94 | 29.01 | 6.07 | 3.90 | 0.0246 | 1.64 | 0.4242 |
| Novel_012157 | XLOC_314013 | 0.69 | 22.10 | 0.00 | 5.01 | 0.0082 | -12.75 | 0.4242 |
| Novel_012627 | XLOC_325684 | 2.30 | 34.16 | 2.89 | 3.89 | 0.0175 | 0.33 | 0.9425 |
| Novel_012634 | XLOC_325684 | 0.24 | 7.25 | 0.99 | 4.91 | 0.0127 | 2.03 | 0.4242 |
| Novel_012856 | XLOC_329728 | 60.11 | 2.76 | 29.81 | -4.44 | 0.0099 | -1.01 | 0.6135 |
| Novel_012930 | XLOC_330802 | 119.05 | 0.74 | 123.43 | -7.33 | 0.0000 | 0.05 | 0.9937 |
| Novel_012934 | XLOC_330823 | 40.43 | 0.24 | 11.36 | -7.38 | 0.0000 | -1.83 | 0.4242 |
| Novel_012962 | XLOC_331257 | 20.64 | 1.31 | 13.86 | -3.98 | 0.0177 | -0.57 | 0.8347 |
| Novel_012984 | XLOC_331619 | 19.32 | 0.33 | 20.97 | -5.86 | 0.0002 | 0.12 | 0.9806 |
| Novel_012988 | XLOC_331668 | 72.59 | 1.09 | 59.96 | -6.05 | 0.0000 | -0.28 | 0.9405 |
| Novel_013025 | XLOC_332429 | 77.86 | 0.83 | 51.91 | -6.55 | 0.0000 | -0.58 | 0.8265 |
| Novel_013031 | XLOC_332483 | 92.19 | 1.56 | 46.99 | -5.89 | 0.0000 | -0.97 | 0.5990 |
| Novel_013040 | XLOC_332720 | 45.50 | 0.54 | 9.99 | -6.40 | 0.0001 | -2.19 | 0.4242 |
| Novel_013041 | XLOC_332733 | 107.14 | 0.33 | 106.10 | -8.35 | 0.0000 | -0.01 | 0.9987 |
| Novel_013053 | XLOC_333007 | 86.41 | 1.39 | 60.68 | -5.96 | 0.0001 | -0.51 | 0.8614 |
| Novel_003895 | XLOC_099594 | 0.34 | 54.35 | 3460.44 | 7.31 | 0.0000 | 13.31 | 0.0000 |
| Novel_005077 | XLOC_130654 | 0.10 | 7.41 | 15.67 | 6.24 | 0.0387 | 7.32 | 0.0051 |
| Novel_008987 | XLOC_234513 | 0.57 | 89.46 | 69.10 | 7.30 | 0.0000 | 6.92 | 0.0000 |
| Novel_005442 | XLOC_139500 | 0.12 | 20.86 | 11.84 | 7.48 | 0.0010 | 6.66 | 0.0073 |
| Novel_003397 | XLOC_088497 | 0.74 | 22.48 | 61.75 | 4.92 | 0.0064 | 6.38 | 0.0000 |
| Novel_006350 | XLOC_161714 | 10.36 | 0.22 | 826.27 | -5.55 | 0.0283 | 6.32 | 0.0000 |
| Novel_004948 | XLOC_127388 | 0.76 | 105.66 | 56.40 | 7.11 | 0.0002 | 6.21 | 0.0035 |
| Novel_005796 | XLOC_144731 | 0.40 | 530.48 | 26.12 | 10.37 | 0.0000 | 6.02 | 0.0044 |
| Novel_006336 | XLOC_160937 | 0.08 | 19.50 | 5.16 | 7.92 | 0.0003 | 6.00 | 0.0296 |
| Novel_005795 | XLOC_144731 | 0.12 | 296.54 | 7.84 | 11.22 | 0.0000 | 5.98 | 0.0448 |
| Novel_000162 | XLOC_004140 | 0.69 | 22.47 | 37.18 | 5.03 | 0.0307 | 5.75 | 0.0032 |
| Novel_002253 | XLOC_062924 | 3.30 | 0.03 | 149.72 | -6.75 | 0.0074 | 5.50 | 0.0000 |
| Novel_005793 | XLOC_144731 | 0.87 | 673.58 | 34.47 | 9.60 | 0.0000 | 5.32 | 0.0307 |
| Novel_008682 | XLOC_226638 | 0.33 | 7.88 | 11.92 | 4.59 | 0.0194 | 5.19 | 0.0030 |
| Novel_007499 | XLOC_191562 | 0.41 | 40.12 | 10.98 | 6.62 | 0.0001 | 4.75 | 0.0396 |
| Novel_007541 | XLOC_194502 | 1.01 | 162.41 | 19.56 | 7.33 | 0.0000 | 4.27 | 0.0335 |
| Novel_002224 | XLOC_061996 | 12.60 | 187.96 | 0.61 | 3.90 | 0.0068 | -4.37 | 0.0167 |
| Novel_001474 | XLOC_040882 | 61.83 | 928.14 | 1.14 | 3.91 | 0.0075 | -5.76 | 0.0002 |
| Novel_011325 | XLOC_291596 | 60.09 | 783.74 | 0.91 | 3.71 | 0.0282 | -6.04 | 0.0002 |
