# Supplementary material for: Integrated analysis of lncRNA and mRNA transcriptomes reveals the potential regulatory role of lncRNA in kiwifruit ripening and softening
Source: Sci Rep. 2021 Jan 18;11:1671. doi: 10.1038/s41598-021-81155-1 (PMC7814023; doi:10.1038/s41598-021-81155-1)
Supplement: Supplementary file 5 — Supplementary Table S3. [file 41598_2021_81155_MOESM5_ESM.doc]

| **Table S3. Differentially expressed genes in ABA vs CK** | | | | | | | | |
| --- | --- | --- | --- | --- | --- | --- | --- | --- |
| **Transcript ID** | **Gene ID** | **FPKM (CK)** | **FPKM (RT)** | **FPKM (ABA)** | **log_2_ FPKM (RTvsCK)** | **corrected *P* value (RTvsCK)** | **log_2_ FPKM (ABAvsCK)** | **corrected *P* value (ABAvsCK)** |
| Achn002501 | Achn002501 | 0.56 | 3.97 | 15.18 | 2.82 | 0.4242 | 4.75 | 0.0104 |
| Achn005701 | Achn005701 | 0.17 | 0.17 | 302.46 | 0.03 | 1.0000 | 10.80 | 0.0000 |
| Achn006051 | Achn006051 | 8.07 | 4.92 | 215.85 | -0.71 | 0.8170 | 4.74 | 0.0005 |
| Achn006131 | Achn006131 | 0.07 | 0.22 | 6.50 | 1.63 | 1.0000 | 6.54 | 0.0118 |
| Achn006331 | Achn006331 | 21.99 | 3.39 | 0.40 | -2.70 | 0.4242 | -5.79 | 0.0016 |
| Achn007481 | Achn007481 | 0.87 | 0.66 | 27.42 | -0.41 | 0.9435 | 4.97 | 0.0040 |
| Achn007661 | Achn007661 | 0.08 | 0.00 | 147.28 | -9.69 | 1.0000 | 10.80 | 0.0000 |
| Achn008121 | Achn008121 | 0.72 | 6.15 | 14.69 | 3.09 | 0.4242 | 4.35 | 0.0272 |
| Achn009121 | Achn009121 | 1.16 | 0.65 | 43.44 | -0.83 | 0.8598 | 5.23 | 0.0023 |
| Achn009241 | Achn009241 | 3.86 | 2.73 | 85.58 | -0.50 | 0.9023 | 4.47 | 0.0025 |
| Achn009281 | Achn009281 | 21.20 | 9.69 | 1.24 | -1.13 | 0.5890 | -4.10 | 0.0413 |
| Achn010851 | Achn010851 | 3.63 | 0.50 | 0.06 | -2.86 | 0.4242 | -5.98 | 0.0377 |
| Achn011201 | Achn011201 | 42.60 | 29.56 | 878.54 | -0.53 | 0.8568 | 4.37 | 0.0421 |
| Achn011691 | Achn011691 | 0.40 | 1.01 | 49.99 | 1.32 | 0.7230 | 6.95 | 0.0001 |
| Achn012261 | Achn012261 | 13.62 | 26.65 | 193.42 | 0.97 | 0.6673 | 3.83 | 0.0121 |
| Achn013661 | Achn013661 | 1.84 | 4.24 | 71.84 | 1.20 | 0.7944 | 5.28 | 0.0353 |
| Achn014701 | Achn014701 | 0.12 | 0.00 | 30.81 | -10.24 | 1.0000 | 7.99 | 0.0003 |
| Achn015041 | Achn015041 | 0.03 | 0.00 | 12.62 | -8.34 | 1.0000 | 8.60 | 0.0001 |
| Achn017661 | Achn017661 | 23.03 | 19.82 | 1.01 | -0.22 | 0.9611 | -4.52 | 0.0157 |
| Achn017771 | Achn017771 | 4.19 | 12.67 | 49.96 | 1.60 | 0.4242 | 3.58 | 0.0498 |
| Achn018501 | Achn018501 | 2.03 | 2.59 | 71.03 | 0.35 | 0.9484 | 5.13 | 0.0011 |
| Achn018511 | Achn018511 | 1.29 | 1.52 | 32.08 | 0.24 | 0.9729 | 4.64 | 0.0232 |
| Achn018971 | Achn018971 | 0.36 | 0.24 | 36.09 | -0.57 | 1.0000 | 6.65 | 0.0000 |
| Achn020161 | Achn020161 | 1.43 | 1.63 | 44.63 | 0.18 | 0.9760 | 4.96 | 0.0009 |
| Achn020491 | Achn020491 | 22.68 | 20.14 | 295.64 | -0.17 | 0.9750 | 3.70 | 0.0234 |
| Achn020841 | Achn020841 | 1.42 | 1.04 | 37.37 | -0.45 | 0.9386 | 4.71 | 0.0119 |
| Achn021381 | Achn021381 | 10.08 | 31.02 | 294.32 | 1.62 | 0.4242 | 4.87 | 0.0007 |
| Achn022871 | Achn022871 | 0.10 | 0.25 | 6.91 | 1.35 | 1.0000 | 6.15 | 0.0223 |
| Achn024591 | Achn024591 | 3.69 | 3.66 | 281.19 | -0.01 | 0.9997 | 6.25 | 0.0002 |
| Achn024671 | Achn024671 | 27.39 | 27.27 | 334.64 | -0.01 | 0.9998 | 3.61 | 0.0184 |
| Achn024711 | Achn024711 | 2.83 | 1.96 | 155.67 | -0.54 | 0.9188 | 5.78 | 0.0002 |
| Achn028861 | Achn028861 | 4.53 | 0.00 | 68.10 | -15.47 | 0.4242 | 3.91 | 0.0133 |
| Achn029191 | Achn029191 | 124.89 | 45.65 | 5.77 | -1.45 | 0.4242 | -4.44 | 0.0013 |
| Achn030701 | Achn030701 | 23.67 | 9.07 | 6550.51 | -1.38 | 0.4788 | 8.11 | 0.0000 |
| Achn031281 | Achn031281 | 0.49 | 0.70 | 10.56 | 0.52 | 0.9233 | 4.44 | 0.0393 |
| Achn032181 | Achn032181 | 19.90 | 12.22 | 0.91 | -0.70 | 0.7987 | -4.46 | 0.0147 |
| Achn032981 | Achn032981 | 0.11 | 0.44 | 6.22 | 2.03 | 1.0000 | 5.85 | 0.0334 |
| Achn033441 | Achn033441 | 40.72 | 10.19 | 0.89 | -2.00 | 0.4242 | -5.52 | 0.0004 |
| Achn033491 | Achn033491 | 1.66 | 1.45 | 27.05 | -0.20 | 0.9756 | 4.03 | 0.0349 |
| Achn034361 | Achn034361 | 4.82 | 1.51 | 119.57 | -1.67 | 0.4479 | 4.63 | 0.0019 |
| Achn034691 | Achn034691 | 1.42 | 0.61 | 56.16 | -1.22 | 0.7307 | 5.31 | 0.0010 |
| Achn035591 | Achn035591 | 0.85 | 7.17 | 492.39 | 3.08 | 0.4242 | 9.18 | 0.0000 |
| Achn036391 | Achn036391 | 84.48 | 521.68 | 1957.46 | 2.63 | 0.2305 | 4.53 | 0.0009 |
| Achn036401 | Achn036401 | 148.23 | 728.88 | 2793.00 | 2.30 | 0.4242 | 4.24 | 0.0403 |
| Achn036621 | Achn036621 | 49.81 | 304.10 | 0.07 | 2.61 | 0.2340 | -9.39 | 0.0000 |
| Achn036741 | Achn036741 | 25.60 | 152.27 | 305.90 | 2.57 | 0.2555 | 3.58 | 0.0190 |
| Achn037201 | Achn037201 | 3.70 | 4.88 | 42.78 | 0.40 | 0.9139 | 3.53 | 0.0265 |
| Achn037901 | Achn037901 | 0.10 | 0.47 | 22.74 | 2.20 | 1.0000 | 7.80 | 0.0004 |
| Achn039701 | Achn039701 | 2.14 | 1.90 | 405.87 | -0.17 | 0.9789 | 7.57 | 0.0000 |
| Achn039921 | Achn039921 | 1.07 | 3.93 | 18.90 | 1.88 | 0.4242 | 4.15 | 0.0212 |
| Achn040581 | Achn040581 | 17.61 | 8.95 | 172.54 | -0.98 | 0.6456 | 3.29 | 0.0433 |
| Achn041061 | Achn041061 | 14.60 | 31.65 | 179.50 | 1.12 | 0.6316 | 3.62 | 0.0400 |
| Achn041311 | Achn041311 | 1.63 | 17.71 | 22.85 | 3.44 | 0.1116 | 3.81 | 0.0416 |
| Achn041411 | Achn041411 | 1.74 | 12.69 | 80.28 | 2.87 | 0.4242 | 5.53 | 0.0002 |
| Achn041431 | Achn041431 | 1.30 | 2.48 | 24.63 | 0.93 | 0.7936 | 4.24 | 0.0289 |
| Achn041641 | Achn041641 | 0.07 | 0.21 | 16.25 | 1.63 | 1.0000 | 7.88 | 0.0004 |
| Achn042601 | Achn042601 | 0.90 | 0.25 | 563.36 | -1.87 | 0.5471 | 9.29 | 0.0000 |
| Achn042701 | Achn042701 | 0.20 | 2.07 | 42.84 | 3.40 | 0.4242 | 7.77 | 0.0001 |
| Achn043471 | Achn043471 | 5.85 | 0.00 | 278.01 | -15.84 | 0.4242 | 5.57 | 0.0001 |
| Achn044801 | Achn044801 | 9.72 | 0.83 | 468.70 | -3.54 | 0.1654 | 5.59 | 0.0000 |
| Achn045661 | Achn045661 | 185.45 | 18.47 | 11.77 | -3.33 | 0.0627 | -3.98 | 0.0115 |
| Achn045721 | Achn045721 | 0.72 | 1.65 | 43.11 | 1.20 | 0.8144 | 5.91 | 0.0196 |
| Achn045991 | Achn045991 | 6.26 | 13.24 | 61.09 | 1.08 | 0.5418 | 3.29 | 0.0448 |
| Achn049401 | Achn049401 | 0.20 | 2.04 | 49.02 | 3.31 | 0.4208 | 7.90 | 0.0000 |
| Achn049931 | Achn049931 | 0.41 | 0.00 | 35.31 | -12.01 | 1.0000 | 6.42 | 0.0003 |
| Achn049941 | Achn049941 | 1.01 | 0.33 | 39.90 | -1.63 | 0.5946 | 5.30 | 0.0011 |
| Achn051011 | Achn051011 | 10.98 | 19.04 | 251.39 | 0.79 | 0.7844 | 4.52 | 0.0017 |
| Achn053201 | Achn053201 | 2.11 | 2.10 | 30.40 | 0.00 | 0.9999 | 3.85 | 0.0269 |
| Achn053351 | Achn053351 | 1.20 | 7.69 | 26.10 | 2.68 | 0.4242 | 4.44 | 0.0149 |
| Achn053361 | Achn053361 | 10.01 | 2.86 | 242.50 | -1.81 | 0.4242 | 4.60 | 0.0009 |
| Achn056341 | Achn056341 | 0.33 | 0.11 | 9.36 | -1.55 | 1.0000 | 4.82 | 0.0334 |
| Achn058421 | Achn058421 | 0.27 | 0.00 | 29.94 | -11.39 | 1.0000 | 6.81 | 0.0006 |
| Achn058881 | Achn058881 | 2.64 | 9.00 | 64.67 | 1.77 | 0.4242 | 4.62 | 0.0016 |
| Achn059081 | Achn059081 | 0.17 | 0.17 | 19.89 | 0.06 | 1.0000 | 6.90 | 0.0042 |
| Achn059231 | Achn059231 | 2.97 | 0.43 | 45.90 | -2.78 | 0.4242 | 3.95 | 0.0386 |
| Achn059531 | Achn059531 | 6.45 | 23.35 | 197.99 | 1.85 | 0.4242 | 4.94 | 0.0010 |
| Achn059761 | Achn059761 | 0.26 | 0.13 | 30.33 | -0.99 | 1.0000 | 6.85 | 0.0000 |
| Achn061351 | Achn061351 | 19.18 | 7.48 | 1.17 | -1.36 | 0.4570 | -4.04 | 0.0367 |
| Achn061651 | Achn061651 | 1.54 | 0.31 | 23.03 | -2.30 | 0.4242 | 3.91 | 0.0500 |
| Achn061721 | Achn061721 | 16.32 | 8.55 | 1.00 | -0.93 | 0.6827 | -4.03 | 0.0362 |
| Achn061931 | Achn061931 | 6.77 | 25.56 | 198.91 | 1.92 | 0.4242 | 4.88 | 0.0004 |
| Achn062471 | Achn062471 | 3.39 | 6.52 | 100.29 | 0.94 | 0.7627 | 4.89 | 0.0015 |
| Achn062991 | Achn062991 | 0.36 | 0.86 | 33.85 | 1.24 | 0.7778 | 6.54 | 0.0014 |
| Achn064481 | Achn064481 | 2.56 | 0.26 | 236.19 | -3.31 | 0.4242 | 6.53 | 0.0000 |
| Achn064581 | Achn064581 | 9.95 | 13.60 | 179.66 | 0.45 | 0.8953 | 4.17 | 0.0030 |
| Achn065591 | Achn065591 | 0.28 | 0.05 | 15.47 | -2.55 | 1.0000 | 5.79 | 0.0022 |
| Achn067071 | Achn067071 | 0.53 | 0.43 | 22.76 | -0.31 | 1.0000 | 5.42 | 0.0249 |
| Achn067151 | Achn067151 | 2.48 | 7.17 | 107.10 | 1.53 | 0.4242 | 5.43 | 0.0001 |
| Achn067861 | Achn067861 | 24.99 | 114.37 | 427.97 | 2.19 | 0.4242 | 4.10 | 0.0051 |
| Achn070291 | Achn070291 | 1.33 | 0.80 | 130.02 | -0.72 | 0.8641 | 6.61 | 0.0000 |
| Achn072451 | Achn072451 | 1.27 | 0.00 | 3926.68 | -13.63 | 0.4242 | 11.60 | 0.0000 |
| Achn072801 | Achn072801 | 0.17 | 0.91 | 15.26 | 2.40 | 0.4616 | 6.47 | 0.0150 |
| Achn073761 | Achn073761 | 9.52 | 0.66 | 0.20 | -3.84 | 0.1685 | -5.55 | 0.0418 |
| Achn076211 | Achn076211 | 3.52 | 0.00 | 62.44 | -15.10 | 0.4242 | 4.15 | 0.0403 |
| Achn076481 | Achn076481 | 0.11 | 0.00 | 6.78 | -10.12 | 1.0000 | 5.92 | 0.0412 |
| Achn077851 | Achn077851 | 17.23 | 96.70 | 0.43 | 2.49 | 0.3109 | -5.31 | 0.0015 |
| Achn079501 | Achn079501 | 4.91 | 28.74 | 439.78 | 2.55 | 0.4242 | 6.48 | 0.0000 |
| Achn080351 | Achn080351 | 14.85 | 95.19 | 351.41 | 2.68 | 0.3594 | 4.56 | 0.0015 |
| Achn080421 | Achn080421 | 0.17 | 0.09 | 9.91 | -0.94 | 1.0000 | 5.89 | 0.0372 |
| Achn080501 | Achn080501 | 0.13 | 0.00 | 26.53 | -10.34 | 1.0000 | 7.68 | 0.0007 |
| Achn082041 | Achn082041 | 32.42 | 22.04 | 1.24 | -0.56 | 0.8491 | -4.71 | 0.0039 |
| Achn083071 | Achn083071 | 0.07 | 0.08 | 5.10 | 0.05 | 1.0000 | 6.14 | 0.0270 |
| Achn083201 | Achn083201 | 19.23 | 12.35 | 271.76 | -0.64 | 0.8516 | 3.82 | 0.0135 |
| Achn084131 | Achn084131 | 2.96 | 4.71 | 43.17 | 0.67 | 0.8657 | 3.87 | 0.0460 |
| Achn084151 | Achn084151 | 0.69 | 0.58 | 11.49 | -0.25 | 0.9656 | 4.06 | 0.0274 |
| Achn084161 | Achn084161 | 101.34 | 24.38 | 4.12 | -2.06 | 0.4242 | -4.62 | 0.0028 |
| Achn085581 | Achn085581 | 9.35 | 6.51 | 145.68 | -0.52 | 0.8893 | 3.96 | 0.0091 |
| Achn085591 | Achn085591 | 0.23 | 0.68 | 37.09 | 1.60 | 0.6876 | 7.36 | 0.0005 |
| Achn086971 | Achn086971 | 2.40 | 4.56 | 87.26 | 0.93 | 0.7573 | 5.18 | 0.0003 |
| Achn087081 | Achn087081 | 6.04 | 4.90 | 138.88 | -0.30 | 0.9514 | 4.52 | 0.0022 |
| Achn087531 | Achn087531 | 3.90 | 6.28 | 45.55 | 0.69 | 0.8244 | 3.54 | 0.0363 |
| Achn088991 | Achn088991 | 1.13 | 0.27 | 19.58 | -2.07 | 0.4242 | 4.12 | 0.0127 |
| Achn089001 | Achn089001 | 0.59 | 0.19 | 14.47 | -1.67 | 0.6199 | 4.60 | 0.0163 |
| Achn089011 | Achn089011 | 1.25 | 0.77 | 15.66 | -0.70 | 0.8288 | 3.65 | 0.0243 |
| Achn089401 | Achn089401 | 1.57 | 0.75 | 58.61 | -1.06 | 0.7016 | 5.22 | 0.0002 |
| Achn089411 | Achn089411 | 0.53 | 0.62 | 49.94 | 0.22 | 0.9846 | 6.55 | 0.0096 |
| Achn089421 | Achn089421 | 2.94 | 0.62 | 502.56 | -2.24 | 0.4992 | 7.42 | 0.0000 |
| Achn090851 | Achn090851 | 2.57 | 16.46 | 412.16 | 2.68 | 0.4242 | 7.32 | 0.0000 |
| Achn092431 | Achn092431 | 3.62 | 1.25 | 116.83 | -1.53 | 0.5341 | 5.01 | 0.0006 |
| Achn093841 | Achn093841 | 0.35 | 0.31 | 15.90 | -0.16 | 1.0000 | 5.50 | 0.0028 |
| Achn093931 | Achn093931 | 1.20 | 8.23 | 74.23 | 2.78 | 0.4242 | 5.95 | 0.0415 |
| Achn095741 | Achn095741 | 0.51 | 5.34 | 40.53 | 3.40 | 0.2235 | 6.32 | 0.0000 |
| Achn096411 | Achn096411 | 1.62 | 3.05 | 134.19 | 0.91 | 0.9018 | 6.37 | 0.0186 |
| Achn096681 | Achn096681 | 0.57 | 0.58 | 50.48 | 0.01 | 0.9994 | 6.46 | 0.0000 |
| Achn098281 | Achn098281 | 0.12 | 0.61 | 18.51 | 2.36 | 0.4622 | 7.29 | 0.0016 |
| Achn098891 | Achn098891 | 0.56 | 0.17 | 21.41 | -1.75 | 0.6679 | 5.27 | 0.0074 |
| Achn100271 | Achn100271 | 0.36 | 0.00 | 11.76 | -11.82 | 1.0000 | 5.02 | 0.0121 |
| Achn100611 | Achn100611 | 3.99 | 3.99 | 360.51 | 0.00 | 0.9999 | 6.50 | 0.0000 |
| Achn102161 | Achn102161 | 0.53 | 0.16 | 22.41 | -1.75 | 1.0000 | 5.39 | 0.0052 |
| Achn102251 | Achn102251 | 17.12 | 0.50 | 0.22 | -5.10 | 0.1417 | -6.29 | 0.0205 |
| Achn102291 | Achn102291 | 5.01 | 6.09 | 382.19 | 0.28 | 0.9508 | 6.25 | 0.0000 |
| Achn102911 | Achn102911 | 6.93 | 1.55 | 321.84 | -2.16 | 0.4242 | 5.54 | 0.0000 |
| Achn103301 | Achn103301 | 0.15 | 0.18 | 13.00 | 0.23 | 1.0000 | 6.41 | 0.0126 |
| Achn104831 | Achn104831 | 1.23 | 7.99 | 26.13 | 2.70 | 0.4242 | 4.41 | 0.0421 |
| Achn105651 | Achn105651 | 24.28 | 1.52 | 0.85 | -4.00 | 0.1408 | -4.84 | 0.0483 |
| Achn106081 | Achn106081 | 3.67 | 4.39 | 455.13 | 0.26 | 0.9747 | 6.95 | 0.0000 |
| Achn106271 | Achn106271 | 0.42 | 0.29 | 7.28 | -0.53 | 1.0000 | 4.11 | 0.0466 |
| Achn106461 | Achn106461 | 0.21 | 3.46 | 56.70 | 4.04 | 0.3217 | 8.08 | 0.0000 |
| Achn107271 | Achn107271 | 0.07 | 0.07 | 4.17 | 0.05 | 1.0000 | 5.86 | 0.0462 |
| Achn108141 | Achn108141 | 30.64 | 6.71 | 359.04 | -2.19 | 0.4242 | 3.55 | 0.0226 |
| Achn108981 | Achn108981 | 2.01 | 0.50 | 0.03 | -2.02 | 0.4242 | -6.13 | 0.0285 |
| Achn109331 | Achn109331 | 26.25 | 41.48 | 3755.32 | 0.66 | 0.9370 | 7.16 | 0.0027 |
| Achn111671 | Achn111671 | 1.50 | 0.19 | 27.78 | -3.01 | 0.4242 | 4.22 | 0.0124 |
| Achn111981 | Achn111981 | 1.11 | 2.54 | 55.21 | 1.20 | 0.7347 | 5.64 | 0.0019 |
| Achn113131 | Achn113131 | 0.20 | 0.20 | 61.13 | 0.05 | 1.0000 | 8.28 | 0.0000 |
| Achn113721 | Achn113721 | 2.66 | 1.52 | 97.17 | -0.81 | 0.8255 | 5.19 | 0.0004 |
| Achn115201 | Achn115201 | 1.76 | 4.60 | 47.93 | 1.38 | 0.4922 | 4.76 | 0.0010 |
| Achn115911 | Achn115911 | 42.29 | 38.73 | 1.59 | -0.13 | 0.9796 | -4.73 | 0.0057 |
| Achn117451 | Achn117451 | 0.51 | 0.21 | 69.38 | -1.30 | 1.0000 | 7.08 | 0.0000 |
| Achn118371 | Achn118371 | 0.08 | 0.00 | 12.20 | -9.70 | 1.0000 | 7.20 | 0.0020 |
| Achn119231 | Achn119231 | 22.50 | 11.62 | 0.93 | -0.95 | 0.6761 | -4.60 | 0.0123 |
| Achn119681 | Achn119681 | 58.38 | 16.74 | 0.95 | -1.80 | 0.4242 | -5.94 | 0.0001 |
| Achn119831 | Achn119831 | 0.58 | 1.32 | 79.28 | 1.19 | 0.7560 | 7.09 | 0.0000 |
| Achn119841 | Achn119841 | 0.05 | 0.05 | 5.22 | 0.03 | 1.0000 | 6.79 | 0.0068 |
| Achn121521 | Achn121521 | 0.48 | 0.19 | 12.85 | -1.29 | 1.0000 | 4.75 | 0.0163 |
| Achn122211 | Achn122211 | 0.04 | 0.00 | 5.77 | -8.69 | 1.0000 | 7.12 | 0.0030 |
| Achn123051 | Achn123051 | 0.59 | 0.00 | 17.71 | -12.52 | 0.4242 | 4.92 | 0.0104 |
| Achn123061 | Achn123061 | 0.66 | 0.23 | 64.37 | -1.50 | 0.7476 | 6.60 | 0.0001 |
| Achn125151 | Achn125151 | 2.01 | 0.24 | 26.60 | -3.06 | 0.4242 | 3.72 | 0.0398 |
| Achn125831 | Achn125831 | 635.63 | 904.27 | 46.40 | 0.51 | 0.8803 | -3.78 | 0.0107 |
| Achn126731 | Achn126731 | 112.86 | 1086.08 | 1963.73 | 3.27 | 0.0740 | 4.12 | 0.0128 |
| Achn126941 | Achn126941 | 0.33 | 0.19 | 14.07 | -0.83 | 1.0000 | 5.41 | 0.0027 |
| Achn126951 | Achn126951 | 0.33 | 0.19 | 14.11 | -0.83 | 1.0000 | 5.41 | 0.0026 |
| Achn128401 | Achn128401 | 0.42 | 6.06 | 12.03 | 3.83 | 0.2232 | 4.82 | 0.0253 |
| Achn130271 | Achn130271 | 2.89 | 12.78 | 42.70 | 2.15 | 0.4242 | 3.89 | 0.0229 |
| Achn130721 | Achn130721 | 22.59 | 129.45 | 241.41 | 2.52 | 0.3144 | 3.42 | 0.0321 |
| Achn131031 | Achn131031 | 0.88 | 0.00 | 35.88 | -13.11 | 0.4242 | 5.34 | 0.0018 |
| Achn132211 | Achn132211 | 0.22 | 1.01 | 27.93 | 2.17 | 0.4857 | 6.96 | 0.0015 |
| Achn132361 | Achn132361 | 0.31 | 0.50 | 14.40 | 0.68 | 1.0000 | 5.52 | 0.0035 |
| Achn132821 | Achn132821 | 0.09 | 0.00 | 9.97 | -9.81 | 1.0000 | 6.80 | 0.0067 |
| Achn133361 | Achn133361 | 1.07 | 3.93 | 18.90 | 1.88 | 0.4242 | 4.15 | 0.0212 |
| Achn134171 | Achn134171 | 56.31 | 90.83 | 1151.87 | 0.69 | 0.7765 | 4.35 | 0.0020 |
| Achn135551 | Achn135551 | 6.90 | 19.83 | 102.34 | 1.52 | 0.4242 | 3.89 | 0.0160 |
| Achn135561 | Achn135561 | 12.13 | 140.62 | 241.86 | 3.53 | 0.0799 | 4.32 | 0.0074 |
| Achn137671 | Achn137671 | 65.45 | 60.79 | 731.81 | -0.11 | 0.9831 | 3.48 | 0.0274 |
| Achn137801 | Achn137801 | 2.14 | 1.90 | 35.64 | -0.17 | 0.9793 | 4.06 | 0.0308 |
| Achn138321 | Achn138321 | 1.87 | 0.27 | 135.17 | -2.81 | 0.4242 | 6.18 | 0.0003 |
| Achn138791 | Achn138791 | 0.96 | 1.53 | 26.77 | 0.67 | 0.8953 | 4.80 | 0.0216 |
| Achn140071 | Achn140071 | 0.16 | 1.24 | 54.94 | 2.96 | 0.4242 | 8.44 | 0.0001 |
| Achn140081 | Achn140081 | 0.77 | 0.00 | 51.57 | -12.90 | 0.4242 | 6.07 | 0.0002 |
| Achn140971 | Achn140971 | 674.16 | 548.09 | 29.84 | -0.30 | 0.9382 | -4.50 | 0.0009 |
| Achn141481 | Achn141481 | 6.77 | 1.47 | 400.37 | -2.20 | 0.4242 | 5.89 | 0.0001 |
| Achn142561 | Achn142561 | 0.08 | 0.00 | 6.62 | -9.61 | 1.0000 | 6.41 | 0.0157 |
| Achn143491 | Achn143491 | 2.27 | 2.17 | 57.16 | -0.07 | 0.9940 | 4.65 | 0.0076 |
| Achn143641 | Achn143641 | 1.66 | 0.93 | 46.98 | -0.83 | 0.8816 | 4.83 | 0.0212 |
| Achn143651 | Achn143651 | 0.36 | 0.04 | 46.42 | -3.08 | 1.0000 | 7.02 | 0.0000 |
| Achn144571 | Achn144571 | 12.24 | 45.09 | 177.44 | 1.88 | 0.4242 | 3.86 | 0.0113 |
| Achn147251 | Achn147251 | 2.33 | 3.75 | 76.28 | 0.68 | 0.8546 | 5.03 | 0.0007 |
| Achn147791 | Achn147791 | 1.72 | 0.61 | 23.18 | -1.48 | 0.5471 | 3.75 | 0.0410 |
| Achn148231 | Achn148231 | 0.26 | 0.14 | 20.68 | -0.95 | 1.0000 | 6.29 | 0.0028 |
| Achn148621 | Achn148621 | 0.74 | 0.57 | 75.34 | -0.38 | 0.9678 | 6.67 | 0.0032 |
| Achn149051 | Achn149051 | 2.01 | 0.32 | 86.18 | -2.66 | 0.4242 | 5.42 | 0.0001 |
| Achn149381 | Achn149381 | 1.85 | 2.46 | 192.93 | 0.41 | 0.9393 | 6.70 | 0.0000 |
| Achn149591 | Achn149591 | 1.30 | 17.35 | 77.05 | 3.74 | 0.2684 | 5.89 | 0.0011 |
| Achn150011 | Achn150011 | 1.31 | 0.00 | 267.68 | -13.67 | 0.4242 | 7.68 | 0.0006 |
| Achn150021 | Achn150021 | 0.27 | 0.33 | 125.08 | 0.30 | 1.0000 | 8.85 | 0.0000 |
| Achn150821 | Achn150821 | 3.48 | 2.68 | 54.23 | -0.38 | 0.9359 | 3.96 | 0.0164 |
| Achn150951 | Achn150951 | 22.32 | 16.40 | 0.14 | -0.44 | 0.9107 | -7.36 | 0.0017 |
| Achn151681 | Achn151681 | 0.67 | 0.00 | 35.76 | -12.71 | 0.4242 | 5.74 | 0.0004 |
| Achn152571 | Achn152571 | 62.03 | 17.59 | 0.38 | -1.82 | 0.4242 | -7.37 | 0.0000 |
| Achn153791 | Achn153791 | 0.07 | 0.19 | 39.59 | 1.34 | 1.0000 | 9.06 | 0.0000 |
| Achn154301 | Achn154301 | 17.31 | 8.81 | 0.21 | -0.97 | 0.6612 | -6.35 | 0.0008 |
| Achn155371 | Achn155371 | 2.51 | 3.23 | 40.26 | 0.37 | 0.9391 | 4.01 | 0.0199 |
| Achn155541 | Achn155541 | 0.96 | 0.31 | 604.96 | -1.65 | 0.6288 | 9.29 | 0.0000 |
| Achn155671 | Achn155671 | 9.24 | 3.94 | 192.12 | -1.23 | 0.5595 | 4.38 | 0.0017 |
| Achn156701 | Achn156701 | 3.98 | 2.47 | 99.65 | -0.69 | 0.8313 | 4.64 | 0.0008 |
| Achn157081 | Achn157081 | 111.30 | 84.15 | 5.60 | -0.40 | 0.8996 | -4.31 | 0.0050 |
| Achn157211 | Achn157211 | 1.69 | 16.22 | 308.91 | 3.26 | 0.2370 | 7.51 | 0.0000 |
| Achn157331 | Achn157331 | 28.74 | 14.00 | 1.16 | -1.04 | 0.6247 | -4.63 | 0.0102 |
| Achn157431 | Achn157431 | 10.48 | 32.91 | 134.31 | 1.65 | 0.4242 | 3.68 | 0.0265 |
| Achn157511 | Achn157511 | 0.51 | 1.11 | 37.03 | 1.14 | 0.8301 | 6.19 | 0.0103 |
| Achn158811 | Achn158811 | 0.61 | 9.34 | 22.12 | 3.95 | 0.1926 | 5.19 | 0.0105 |
| Achn159051 | Achn159051 | 49.25 | 34.24 | 1103.56 | -0.52 | 0.8620 | 4.49 | 0.0012 |
| Achn159201 | Achn159201 | 0.39 | 1.38 | 132.91 | 1.84 | 0.6038 | 8.43 | 0.0000 |
| Achn159661 | Achn159661 | 1.42 | 0.20 | 145.27 | -2.84 | 0.4242 | 6.67 | 0.0000 |
| Achn159771 | Achn159771 | 0.13 | 0.09 | 118.83 | -0.56 | 1.0000 | 9.89 | 0.0000 |
| Achn159951 | Achn159951 | 0.14 | 0.07 | 6.35 | -0.98 | 1.0000 | 5.48 | 0.0217 |
| Achn160151 | Achn160151 | 1.24 | 11.28 | 60.15 | 3.18 | 0.2440 | 5.59 | 0.0001 |
| Achn160161 | Achn160161 | 0.10 | 0.52 | 10.68 | 2.40 | 1.0000 | 6.76 | 0.0072 |
| Achn160581 | Achn160581 | 0.06 | 0.06 | 9.55 | 0.04 | 1.0000 | 7.42 | 0.0015 |
| Achn160751 | Achn160751 | 0.62 | 0.04 | 14.96 | -3.80 | 0.4242 | 4.59 | 0.0056 |
| Achn162861 | Achn162861 | 244.32 | 60.81 | 8.06 | -2.01 | 0.4242 | -4.92 | 0.0061 |
| Achn163281 | Achn163281 | 9.10 | 9.77 | 0.06 | 0.10 | 0.9839 | -7.27 | 0.0000 |
| Achn163771 | Achn163771 | 46.64 | 192.88 | 787.66 | 2.05 | 0.4242 | 4.08 | 0.0041 |
| Achn164551 | Achn164551 | 16.39 | 19.93 | 190.46 | 0.28 | 0.9418 | 3.54 | 0.0213 |
| Achn164741 | Achn164741 | 0.09 | 0.00 | 436.08 | -9.76 | 1.0000 | 12.29 | 0.0000 |
| Achn165131 | Achn165131 | 1.31 | 2.53 | 25.46 | 0.95 | 0.7713 | 4.28 | 0.0167 |
| Achn165281 | Achn165281 | 19.85 | 4.29 | 0.77 | -2.21 | 0.4242 | -4.69 | 0.0210 |
| Achn165841 | Achn165841 | 21.10 | 43.48 | 0.24 | 1.04 | 0.5509 | -6.44 | 0.0000 |
| Achn166151 | Achn166151 | 0.76 | 1.08 | 33.15 | 0.51 | 0.9209 | 5.45 | 0.0010 |
| Achn166241 | Achn166241 | 0.78 | 0.00 | 56.54 | -12.93 | 0.4242 | 6.18 | 0.0003 |
| Achn166341 | Achn166341 | 1.01 | 0.88 | 20.46 | -0.21 | 0.9735 | 4.34 | 0.0133 |
| Achn166351 | Achn166351 | 10.89 | 7.37 | 125.04 | -0.56 | 0.8595 | 3.52 | 0.0236 |
| Achn166581 | Achn166581 | 48.25 | 34.66 | 636.00 | -0.48 | 0.8952 | 3.72 | 0.0143 |
| Achn167271 | Achn167271 | 20.08 | 3.48 | 467.47 | -2.53 | 0.4242 | 4.54 | 0.0009 |
| Achn167651 | Achn167651 | 8.20 | 1.99 | 282.48 | -2.05 | 0.4242 | 5.11 | 0.0002 |
| Achn167911 | Achn167911 | 213.43 | 53.27 | 2116.17 | -2.00 | 0.4242 | 3.31 | 0.0406 |
| Achn168001 | Achn168001 | 5.00 | 3.07 | 276.61 | -0.70 | 0.8552 | 5.79 | 0.0000 |
| Achn169151 | Achn169151 | 0.40 | 0.12 | 13.82 | -1.77 | 1.0000 | 5.10 | 0.0121 |
| Achn169641 | Achn169641 | 3.14 | 15.82 | 109.45 | 2.33 | 0.4242 | 5.12 | 0.0003 |
| Achn169701 | Achn169701 | 0.19 | 0.21 | 10.90 | 0.15 | 1.0000 | 5.84 | 0.0482 |
| Achn170441 | Achn170441 | 0.45 | 0.00 | 40.75 | -12.13 | 1.0000 | 6.51 | 0.0001 |
| Achn170471 | Achn170471 | 1.39 | 0.65 | 82.56 | -1.09 | 0.7574 | 5.89 | 0.0001 |
| Achn170571 | Achn170571 | 6.11 | 4.02 | 83.18 | -0.60 | 0.8668 | 3.77 | 0.0189 |
| Achn172201 | Achn172201 | 0.62 | 0.88 | 21.14 | 0.50 | 0.9165 | 5.09 | 0.0016 |
| Achn172271 | Achn172271 | 0.34 | 1.67 | 11.70 | 2.31 | 0.4242 | 5.11 | 0.0490 |
| Achn172281 | Achn172281 | 112.38 | 518.07 | 2616.04 | 2.20 | 0.4242 | 4.54 | 0.0017 |
| Achn172521 | Achn172521 | 0.23 | 0.00 | 30.74 | -11.17 | 1.0000 | 7.06 | 0.0036 |
| Achn172971 | Achn172971 | 23.43 | 0.00 | 794.60 | -17.84 | 0.4242 | 5.08 | 0.0003 |
| Achn172981 | Achn172981 | 17.01 | 1.92 | 2093.57 | -3.15 | 0.4242 | 6.94 | 0.0000 |
| Achn172991 | Achn172991 | 3.54 | 0.00 | 1360.24 | -15.11 | 0.4242 | 8.58 | 0.0000 |
| Achn173001 | Achn173001 | 18.17 | 1.92 | 5707.62 | -3.24 | 0.4242 | 8.30 | 0.0000 |
| Achn174841 | Achn174841 | 1.12 | 0.42 | 16.78 | -1.42 | 0.6070 | 3.90 | 0.0431 |
| Achn174931 | Achn174931 | 46.58 | 196.84 | 1324.80 | 2.08 | 0.4242 | 4.83 | 0.0022 |
| Achn175011 | Achn175011 | 1.89 | 3.68 | 69.25 | 0.96 | 0.7408 | 5.20 | 0.0003 |
| Achn175611 | Achn175611 | 261.37 | 24.12 | 3.07 | -3.44 | 0.0618 | -6.41 | 0.0000 |
| Achn176951 | Achn176951 | 15.24 | 1.08 | 170.26 | -3.82 | 0.1767 | 3.48 | 0.0437 |
| Achn177191 | Achn177191 | 18.83 | 1.38 | 478.17 | -3.77 | 0.4242 | 4.67 | 0.0022 |
| Achn179541 | Achn179541 | 126.60 | 182.03 | 1685.79 | 0.52 | 0.8536 | 3.74 | 0.0161 |
| Achn180001 | Achn180001 | 1.13 | 0.00 | 968.93 | -13.47 | 0.4242 | 9.74 | 0.0000 |
| Achn182271 | Achn182271 | 0.45 | 2.17 | 8.52 | 2.28 | 0.4242 | 4.25 | 0.0196 |
| Achn183171 | Achn183171 | 0.10 | 1.21 | 20.50 | 3.54 | 0.4242 | 7.62 | 0.0009 |
| Achn183801 | Achn183801 | 0.04 | 0.15 | 3.33 | 2.02 | 1.0000 | 6.47 | 0.0137 |
| Achn183981 | Achn183981 | 16.43 | 33.41 | 372.87 | 1.02 | 0.7454 | 4.50 | 0.0086 |
| Achn187281 | Achn187281 | 1.52 | 0.00 | 298.45 | -13.89 | 0.4242 | 7.62 | 0.0000 |
| Achn188121 | Achn188121 | 10.39 | 24.16 | 883.66 | 1.22 | 0.6270 | 6.41 | 0.0000 |
| Achn188441 | Achn188441 | 0.07 | 0.09 | 7.11 | 0.46 | 1.0000 | 6.70 | 0.0029 |
| Achn189151 | Achn189151 | 1.01 | 0.00 | 395.82 | -13.30 | 0.4242 | 8.62 | 0.0000 |
| Achn189911 | Achn189911 | 144.36 | 620.15 | 14.52 | 2.10 | 0.4242 | -3.31 | 0.0412 |
| Achn192931 | Achn192931 | 2.84 | 3.13 | 117.05 | 0.14 | 0.9886 | 5.37 | 0.0083 |
| Achn193451 | Achn193451 | 5.36 | 0.20 | 723.16 | -4.74 | 0.1724 | 7.08 | 0.0000 |
| Achn193641 | Achn193641 | 17.33 | 14.09 | 716.00 | -0.30 | 0.9581 | 5.37 | 0.0003 |
| Achn194401 | Achn194401 | 10.69 | 9.70 | 202.99 | -0.14 | 0.9779 | 4.25 | 0.0023 |
| Achn195841 | Achn195841 | 25.32 | 131.84 | 319.34 | 2.38 | 0.4242 | 3.66 | 0.0169 |
| Achn197061 | Achn197061 | 2.63 | 0.08 | 0.04 | -5.11 | 0.1442 | -6.22 | 0.0240 |
| Achn197921 | Achn197921 | 46.81 | 5.20 | 3.18 | -3.17 | 0.0898 | -3.88 | 0.0151 |
| Achn198621 | Achn198621 | 0.37 | 0.25 | 21.76 | -0.54 | 1.0000 | 5.88 | 0.0001 |
| Achn198891 | Achn198891 | 7.68 | 9.53 | 0.13 | 0.31 | 0.9502 | -5.90 | 0.0436 |
| Achn204401 | Achn204401 | 7.01 | 27.58 | 79.76 | 1.98 | 0.4242 | 3.51 | 0.0351 |
| Achn206641 | Achn206641 | 0.09 | 0.19 | 6.84 | 1.02 | 1.0000 | 6.18 | 0.0103 |
| Achn206791 | Achn206791 | 12.40 | 4.07 | 4712.46 | -1.61 | 0.4242 | 8.57 | 0.0000 |
| Achn207361 | Achn207361 | 1.72 | 0.13 | 72.69 | -3.78 | 0.4242 | 5.40 | 0.0002 |
| Achn207871 | Achn207871 | 11.41 | 3.51 | 0.31 | -1.70 | 0.4242 | -5.22 | 0.0404 |
| Achn210461 | Achn210461 | 0.29 | 0.15 | 63.38 | -0.95 | 1.0000 | 7.80 | 0.0000 |
| Achn211501 | Achn211501 | 43.16 | 83.60 | 1.54 | 0.95 | 0.6142 | -4.81 | 0.0024 |
| Achn212161 | Achn212161 | 6.56 | 19.52 | 84.89 | 1.57 | 0.4242 | 3.69 | 0.0289 |
| Achn213151 | Achn213151 | 3.25 | 2.96 | 36.80 | -0.13 | 0.9813 | 3.50 | 0.0371 |
| Achn213291 | Achn213291 | 10.81 | 69.39 | 0.29 | 2.68 | 0.2380 | -5.20 | 0.0075 |
| Achn213311 | Achn213311 | 9.07 | 103.77 | 818.37 | 3.52 | 0.0671 | 6.50 | 0.0000 |
| Achn213381 | Achn213381 | 6.03 | 6.13 | 108.00 | 0.02 | 0.9984 | 4.16 | 0.0396 |
| Achn213941 | Achn213941 | 2.79 | 1.32 | 54.94 | -1.08 | 0.7176 | 4.30 | 0.0077 |
| Achn215031 | Achn215031 | 0.45 | 0.23 | 10.92 | -0.97 | 1.0000 | 4.62 | 0.0244 |
| Achn215751 | Achn215751 | 0.09 | 0.00 | 10.67 | -9.73 | 1.0000 | 6.97 | 0.0045 |
| Achn216341 | Achn216341 | 8.56 | 0.73 | 141.82 | -3.56 | 0.3432 | 4.05 | 0.0108 |
| Achn216411 | Achn216411 | 0.27 | 0.00 | 16.02 | -11.37 | 1.0000 | 5.92 | 0.0015 |
| Achn216511 | Achn216511 | 0.03 | 0.03 | 4.35 | 0.00 | 1.0000 | 7.38 | 0.0013 |
| Achn219831 | Achn219831 | 2.59 | 0.51 | 74.13 | -2.35 | 0.4242 | 4.84 | 0.0017 |
| Achn220181 | Achn220181 | 0.34 | 0.64 | 13.47 | 0.91 | 0.8432 | 5.31 | 0.0092 |
| Achn220211 | Achn220211 | 6.16 | 1.70 | 0.08 | -1.86 | 0.4242 | -6.23 | 0.0231 |
| Achn220521 | Achn220521 | 9.54 | 23.51 | 307.63 | 1.30 | 0.5471 | 5.01 | 0.0004 |
| Achn223541 | Achn223541 | 0.18 | 1.12 | 17.70 | 2.65 | 0.4242 | 6.63 | 0.0078 |
| Achn224101 | Achn224101 | 1.15 | 0.20 | 20.48 | -2.54 | 0.4242 | 4.16 | 0.0362 |
| Achn224131 | Achn224131 | 2.88 | 4.92 | 225.38 | 0.77 | 0.8531 | 6.29 | 0.0000 |
| Achn224331 | Achn224331 | 22.08 | 78.69 | 242.20 | 1.83 | 0.4242 | 3.46 | 0.0307 |
| Achn228371 | Achn228371 | 0.72 | 0.28 | 26.86 | -1.35 | 0.7688 | 5.22 | 0.0069 |
| Achn228421 | Achn228421 | 1.37 | 1.99 | 22.45 | 0.53 | 0.9091 | 4.03 | 0.0454 |
| Achn232431 | Achn232431 | 11.03 | 67.52 | 24896.80 | 2.61 | 0.4969 | 11.14 | 0.0000 |
| Achn232441 | Achn232441 | 18.92 | 131.62 | 9647.70 | 2.80 | 0.4242 | 8.99 | 0.0000 |
| Achn232701 | Achn232701 | 0.32 | 0.27 | 17.60 | -0.23 | 1.0000 | 5.79 | 0.0022 |
| Achn233201 | Achn233201 | 91.08 | 109.43 | 1.73 | 0.26 | 0.9476 | -5.72 | 0.0015 |
| Achn233741 | Achn233741 | 0.12 | 0.00 | 87.06 | -10.17 | 1.0000 | 9.56 | 0.0000 |
| Achn234201 | Achn234201 | 29.05 | 18.42 | 1.45 | -0.66 | 0.7941 | -4.32 | 0.0061 |
| Achn234251 | Achn234251 | 970.58 | 203.38 | 70.92 | -2.25 | 0.4242 | -3.77 | 0.0126 |
| Achn235441 | Achn235441 | 0.27 | 2.50 | 61.34 | 3.22 | 0.4242 | 7.84 | 0.0005 |
| Achn236221 | Achn236221 | 28.51 | 35.35 | 323.88 | 0.31 | 0.9446 | 3.51 | 0.0412 |
| Achn236631 | Achn236631 | 2.62 | 0.76 | 78.77 | -1.79 | 0.4804 | 4.91 | 0.0017 |
| Achn237651 | Achn237651 | 2.32 | 0.28 | 44.81 | -3.07 | 0.4242 | 4.27 | 0.0264 |
| Achn238811 | Achn238811 | 386.33 | 2605.42 | 26.59 | 2.75 | 0.4242 | -3.86 | 0.0110 |
| Achn238851 | Achn238851 | 109.42 | 12.90 | 4.36 | -3.08 | 0.1252 | -4.65 | 0.0023 |
| Achn240361 | Achn240361 | 42.32 | 56.66 | 688.96 | 0.42 | 0.9013 | 4.03 | 0.0046 |
| Achn240451 | Achn240451 | 31.08 | 30.75 | 795.72 | -0.02 | 0.9987 | 4.68 | 0.0005 |
| Achn240631 | Achn240631 | 0.25 | 0.66 | 10.88 | 1.37 | 0.7354 | 5.42 | 0.0251 |
| Achn240741 | Achn240741 | 0.42 | 2.59 | 1601.60 | 2.63 | 0.4242 | 11.90 | 0.0000 |
| Achn242531 | Achn242531 | 44.15 | 7.49 | 2178.70 | -2.56 | 0.3335 | 5.62 | 0.0003 |
| Achn244701 | Achn244701 | 4.16 | 0.19 | 54.94 | -4.42 | 0.3617 | 3.72 | 0.0499 |
| Achn245451 | Achn245451 | 0.14 | 0.91 | 60.88 | 2.70 | 0.4242 | 8.76 | 0.0000 |
| Achn249511 | Achn249511 | 53.66 | 26.13 | 874.30 | -1.04 | 0.7302 | 4.03 | 0.0141 |
| Achn249601 | Achn249601 | 0.03 | 0.05 | 3.98 | 1.01 | 1.0000 | 7.23 | 0.0023 |
| Achn251121 | Achn251121 | 10.23 | 46.09 | 246.06 | 2.17 | 0.4242 | 4.59 | 0.0008 |
| Achn251451 | Achn251451 | 2.58 | 0.57 | 49.32 | -2.17 | 0.4242 | 4.26 | 0.0057 |
| Achn251601 | Achn251601 | 0.55 | 1.65 | 22.58 | 1.57 | 0.5401 | 5.35 | 0.0017 |
| Achn251771 | Achn251771 | 1.07 | 2.08 | 212.04 | 0.96 | 0.7813 | 7.63 | 0.0000 |
| Achn251851 | Achn251851 | 2.35 | 7.56 | 86.40 | 1.69 | 0.4522 | 5.20 | 0.0013 |
| Achn252561 | Achn252561 | 52.93 | 31.77 | 581.85 | -0.74 | 0.7956 | 3.46 | 0.0300 |
| Achn253071 | Achn253071 | 12.11 | 18.67 | 357.39 | 0.63 | 0.8383 | 4.88 | 0.0003 |
| Achn253371 | Achn253371 | 38.93 | 12.56 | 1.43 | -1.63 | 0.4242 | -4.76 | 0.0020 |
| Achn253791 | Achn253791 | 0.93 | 0.69 | 22.31 | -0.43 | 0.9299 | 4.59 | 0.0038 |
| Achn255821 | Achn255821 | 11.19 | 7.67 | 260.88 | -0.55 | 0.8863 | 4.54 | 0.0015 |
| Achn256241 | Achn256241 | 22.36 | 6.12 | 0.90 | -1.87 | 0.4242 | -4.63 | 0.0053 |
| Achn258471 | Achn258471 | 2.81 | 0.82 | 72.53 | -1.78 | 0.4242 | 4.69 | 0.0011 |
| Achn259181 | Achn259181 | 462.76 | 193.13 | 26.56 | -1.26 | 0.4348 | -4.12 | 0.0084 |
| Achn261721 | Achn261721 | 6.15 | 0.43 | 94.22 | -3.84 | 0.2193 | 3.94 | 0.0133 |
| Achn263161 | Achn263161 | 0.03 | 0.11 | 3.12 | 2.01 | 1.0000 | 6.86 | 0.0057 |
| Achn263521 | Achn263521 | 0.21 | 1.96 | 12.42 | 3.26 | 0.4242 | 5.92 | 0.0028 |
| Achn264131 | Achn264131 | 1.28 | 0.32 | 19.21 | -1.98 | 0.4242 | 3.91 | 0.0176 |
| Achn264541 | Achn264541 | 0.23 | 4.46 | 51.76 | 4.30 | 0.1101 | 7.83 | 0.0000 |
| Achn265021 | Achn265021 | 0.37 | 0.00 | 14.19 | -11.85 | 1.0000 | 5.27 | 0.0351 |
| Achn266761 | Achn266761 | 0.04 | 0.05 | 6.01 | 0.03 | 1.0000 | 7.09 | 0.0033 |
| Achn266791 | Achn266791 | 2.31 | 6.57 | 69.75 | 1.51 | 0.4960 | 4.92 | 0.0016 |
| Achn266831 | Achn266831 | 9.54 | 18.72 | 113.47 | 0.97 | 0.6248 | 3.57 | 0.0197 |
| Achn267381 | Achn267381 | 2.69 | 0.15 | 85.51 | -4.14 | 0.4242 | 4.99 | 0.0025 |
| Achn267711 | Achn267711 | 1.95 | 0.27 | 0.05 | -2.86 | 0.4242 | -5.21 | 0.0417 |
| Achn268221 | Achn268221 | 0.19 | 0.00 | 7.04 | -10.86 | 1.0000 | 5.25 | 0.0367 |
| Achn268611 | Achn268611 | 3.56 | 19.70 | 61.44 | 2.47 | 0.4242 | 4.11 | 0.0095 |
| Achn269181 | Achn269181 | 0.67 | 0.44 | 25.17 | -0.62 | 0.9170 | 5.23 | 0.0065 |
| Achn269561 | Achn269561 | 1.48 | 9.07 | 64.19 | 2.62 | 0.4242 | 5.44 | 0.0065 |
| Achn269821 | Achn269821 | 0.34 | 0.24 | 23.38 | -0.50 | 1.0000 | 6.12 | 0.0121 |
| Achn270471 | Achn270471 | 0.04 | 0.17 | 5.19 | 2.03 | 1.0000 | 6.95 | 0.0046 |
| Achn271701 | Achn271701 | 1.21 | 0.00 | 455.46 | -13.57 | 0.4242 | 8.55 | 0.0000 |
| Achn272671 | Achn272671 | 0.13 | 0.03 | 15.89 | -2.31 | 1.0000 | 6.98 | 0.0001 |
| Achn273481 | Achn273481 | 119.66 | 320.43 | 2280.26 | 1.42 | 0.4242 | 4.25 | 0.0027 |
| Achn273491 | Achn273491 | 6.49 | 19.39 | 243.44 | 1.58 | 0.4611 | 5.23 | 0.0005 |
| Achn273501 | Achn273501 | 0.30 | 0.15 | 77.12 | -0.98 | 1.0000 | 8.03 | 0.0000 |
| Achn274401 | Achn274401 | 0.51 | 0.36 | 24.70 | -0.49 | 1.0000 | 5.61 | 0.0039 |
| Achn275151 | Achn275151 | 0.74 | 2.86 | 13.93 | 1.95 | 0.4242 | 4.23 | 0.0276 |
| Achn275411 | Achn275411 | 0.27 | 1.38 | 8.44 | 2.38 | 0.4242 | 4.99 | 0.0132 |
| Achn275701 | Achn275701 | 4.72 | 4.86 | 516.66 | 0.04 | 0.9971 | 6.77 | 0.0000 |
| Achn276911 | Achn276911 | 22.03 | 4.85 | 782.57 | -2.18 | 0.4242 | 5.15 | 0.0002 |
| Achn277931 | Achn277931 | 18.27 | 20.54 | 254.15 | 0.17 | 0.9701 | 3.80 | 0.0096 |
| Achn278571 | Achn278571 | 1.34 | 0.18 | 30.19 | -2.94 | 0.4242 | 4.49 | 0.0164 |
| Achn281771 | Achn281771 | 0.29 | 4.89 | 19.87 | 4.09 | 0.2098 | 6.12 | 0.0016 |
| Achn281991 | Achn281991 | 2.30 | 2.12 | 39.05 | -0.12 | 0.9879 | 4.09 | 0.0308 |
| Achn282721 | Achn282721 | 0.64 | 0.67 | 70.54 | 0.07 | 0.9955 | 6.78 | 0.0001 |
| Achn282731 | Achn282731 | 0.21 | 0.00 | 42.52 | -11.07 | 1.0000 | 7.63 | 0.0006 |
| Achn282791 | Achn282791 | 4.36 | 4.77 | 0.05 | 0.13 | 0.9835 | -6.52 | 0.0126 |
| Achn283151 | Achn283151 | 0.58 | 1.10 | 14.56 | 0.92 | 0.8009 | 4.65 | 0.0111 |
| Achn283211 | Achn283211 | 1.31 | 1.10 | 121.41 | -0.25 | 0.9730 | 6.54 | 0.0000 |
| Achn283921 | Achn283921 | 1.51 | 1.08 | 45.98 | -0.49 | 0.9248 | 4.93 | 0.0031 |
| Achn283961 | Achn283961 | 3.74 | 16.43 | 43.27 | 2.13 | 0.4242 | 3.53 | 0.0400 |
| Achn284141 | Achn284141 | 6.88 | 2.55 | 82.02 | -1.43 | 0.4520 | 3.58 | 0.0227 |
| Achn286121 | Achn286121 | 5.88 | 3.07 | 125.36 | -0.94 | 0.7960 | 4.41 | 0.0093 |
| Achn287101 | Achn287101 | 0.88 | 0.89 | 35.39 | 0.02 | 0.9983 | 5.33 | 0.0005 |
| Achn287321 | Achn287321 | 0.55 | 0.13 | 11.28 | -2.09 | 1.0000 | 4.36 | 0.0322 |
| Achn290181 | Achn290181 | 0.09 | 0.00 | 7.45 | -9.79 | 1.0000 | 6.40 | 0.0161 |
| Achn290431 | Achn290431 | 0.07 | 0.00 | 12.51 | -9.43 | 1.0000 | 7.50 | 0.0012 |
| Achn290601 | Achn290601 | 0.23 | 1.39 | 10.05 | 2.61 | 0.4242 | 5.47 | 0.0100 |
| Achn293001 | Achn293001 | 5.02 | 1.60 | 0.06 | -1.65 | 0.4384 | -6.42 | 0.0159 |
| Achn294311 | Achn294311 | 9.25 | 30.94 | 144.82 | 1.74 | 0.4242 | 3.97 | 0.0059 |
| Achn294591 | Achn294591 | 6.49 | 0.65 | 0.30 | -3.32 | 0.2614 | -4.43 | 0.0486 |
| Achn294821 | Achn294821 | 664.49 | 694.40 | 62.39 | 0.06 | 0.9925 | -3.41 | 0.0387 |
| Achn294851 | Achn294851 | 2.67 | 1.77 | 46.97 | -0.59 | 0.8937 | 4.13 | 0.0201 |
| Achn295201 | Achn295201 | 1.35 | 21.68 | 237.55 | 4.00 | 0.4242 | 7.46 | 0.0011 |
| Achn296331 | Achn296331 | 0.65 | 0.00 | 65.62 | -12.67 | 0.4242 | 6.65 | 0.0098 |
| Achn296821 | Achn296821 | 12.16 | 8.56 | 144.09 | -0.51 | 0.8882 | 3.57 | 0.0258 |
| Achn297251 | Achn297251 | 0.17 | 0.52 | 113.40 | 1.65 | 1.0000 | 9.41 | 0.0000 |
| Achn299331 | Achn299331 | 0.89 | 0.76 | 37.54 | -0.23 | 0.9817 | 5.40 | 0.0264 |
| Achn301851 | Achn301851 | 0.41 | 0.00 | 203.94 | -12.00 | 1.0000 | 8.96 | 0.0000 |
| Achn302701 | Achn302701 | 2.01 | 0.61 | 39.79 | -1.72 | 0.4557 | 4.31 | 0.0079 |
| Achn303151 | Achn303151 | 0.21 | 2.15 | 28.02 | 3.33 | 0.4242 | 7.03 | 0.0030 |
| Achn303161 | Achn303161 | 0.16 | 4.25 | 42.30 | 4.76 | 0.2403 | 8.07 | 0.0002 |
| Achn303591 | Achn303591 | 1.79 | 2.44 | 90.66 | 0.44 | 0.9255 | 5.66 | 0.0001 |
| Achn303601 | Achn303601 | 1.41 | 0.68 | 23.05 | -1.05 | 0.7357 | 4.03 | 0.0201 |
| Achn304611 | Achn304611 | 0.08 | 0.00 | 52.11 | -9.59 | 1.0000 | 9.40 | 0.0000 |
| Achn305231 | Achn305231 | 22.79 | 18.85 | 233.60 | -0.27 | 0.9466 | 3.36 | 0.0373 |
| Achn306461 | Achn306461 | 0.14 | 0.05 | 20.30 | -1.56 | 1.0000 | 7.21 | 0.0008 |
| Achn306781 | Achn306781 | 10.23 | 3.32 | 0.14 | -1.62 | 0.4647 | -6.17 | 0.0262 |
| Achn306971 | Achn306971 | 8.52 | 3.99 | 147.44 | -1.10 | 0.7225 | 4.11 | 0.0165 |
| Achn307121 | Achn307121 | 0.10 | 0.31 | 13.97 | 1.66 | 1.0000 | 7.14 | 0.0029 |
| Achn307851 | Achn307851 | 54.33 | 10.57 | 2.63 | -2.36 | 0.4242 | -4.37 | 0.0288 |
| Achn308231 | Achn308231 | 0.53 | 0.14 | 244.41 | -1.90 | 1.0000 | 8.86 | 0.0000 |
| Achn310371 | Achn310371 | 0.46 | 0.59 | 14.83 | 0.37 | 0.9498 | 5.02 | 0.0061 |
| Achn310511 | Achn310511 | 0.92 | 0.00 | 24.86 | -13.17 | 0.4242 | 4.76 | 0.0041 |
| Achn310541 | Achn310541 | 323.15 | 103.29 | 24.69 | -1.65 | 0.4242 | -3.71 | 0.0137 |
| Achn311001 | Achn311001 | 6.74 | 30.22 | 114.22 | 2.16 | 0.4242 | 4.08 | 0.0103 |
| Achn313131 | Achn313131 | 0.63 | 0.89 | 32.99 | 0.49 | 0.9367 | 5.71 | 0.0029 |
| Achn315541 | Achn315541 | 19.94 | 20.87 | 199.28 | 0.07 | 0.9916 | 3.32 | 0.0425 |
| Achn316101 | Achn316101 | 14.27 | 1.40 | 0.21 | -3.35 | 0.2455 | -6.05 | 0.0143 |
| Achn316361 | Achn316361 | 7.73 | 2.59 | 371.19 | -1.58 | 0.5498 | 5.59 | 0.0001 |
| Achn317371 | Achn317371 | 3.54 | 3.92 | 98.20 | 0.15 | 0.9836 | 4.80 | 0.0049 |
| Achn317441 | Achn317441 | 116.50 | 50.25 | 9.44 | -1.21 | 0.4551 | -3.63 | 0.0168 |
| Achn317631 | Achn317631 | 0.26 | 0.96 | 64.17 | 1.90 | 0.6172 | 7.96 | 0.0002 |
| Achn318091 | Achn318091 | 4.18 | 12.44 | 105.75 | 1.57 | 0.4242 | 4.66 | 0.0007 |
| Achn318101 | Achn318101 | 0.36 | 0.00 | 122.47 | -11.80 | 1.0000 | 8.42 | 0.0000 |
| Achn319011 | Achn319011 | 0.02 | 0.02 | 1.35 | 0.01 | 1.0000 | 6.26 | 0.0212 |
| Achn319721 | Achn319721 | 7.03 | 7.15 | 114.72 | 0.02 | 0.9978 | 4.03 | 0.0064 |
| Achn319971 | Achn319971 | 13.10 | 1.95 | 0.19 | -2.75 | 0.4242 | -6.09 | 0.0018 |
| Achn320671 | Achn320671 | 0.80 | 0.89 | 16.72 | 0.16 | 0.9832 | 4.39 | 0.0326 |
| Achn320901 | Achn320901 | 2.61 | 11.46 | 240.14 | 2.13 | 0.4242 | 6.52 | 0.0000 |
| Achn322041 | Achn322041 | 0.24 | 0.08 | 23.31 | -1.53 | 1.0000 | 6.61 | 0.0036 |
| Achn322281 | Achn322281 | 8.81 | 75.12 | 298.11 | 3.09 | 0.0970 | 5.08 | 0.0001 |
| Achn322841 | Achn322841 | 89.15 | 44.22 | 4.33 | -1.01 | 0.5839 | -4.36 | 0.0052 |
| Achn323101 | Achn323101 | 74.20 | 134.15 | 6.39 | 0.85 | 0.6760 | -3.54 | 0.0334 |
| Achn323621 | Achn323621 | 1.06 | 0.24 | 52.14 | -2.13 | 0.4469 | 5.62 | 0.0003 |
| Achn323891 | Achn323891 | 2.89 | 5.73 | 55.22 | 0.99 | 0.6943 | 4.26 | 0.0049 |
| Achn324811 | Achn324811 | 1.00 | 5.44 | 54.53 | 2.44 | 0.4242 | 5.77 | 0.0005 |
| Achn325531 | Achn325531 | 17.05 | 21.74 | 375.11 | 0.35 | 0.9259 | 4.46 | 0.0011 |
| Achn327821 | Achn327821 | 0.61 | 0.26 | 45.64 | -1.23 | 0.8231 | 6.23 | 0.0011 |
| Achn328781 | Achn328781 | 6.02 | 7.34 | 67.62 | 0.28 | 0.9494 | 3.49 | 0.0395 |
| Achn330151 | Achn330151 | 14.48 | 1.67 | 922.52 | -3.12 | 0.3209 | 5.99 | 0.0000 |
| Achn330871 | Achn330871 | 0.55 | 0.00 | 239.60 | -12.43 | 0.4242 | 8.76 | 0.0000 |
| Achn331961 | Achn331961 | 7.93 | 25.29 | 101.31 | 1.67 | 0.4242 | 3.68 | 0.0461 |
| Achn332151 | Achn332151 | 19.63 | 155.23 | 229.40 | 2.98 | 0.1487 | 3.55 | 0.0320 |
| Achn332191 | Achn332191 | 12.39 | 73.52 | 122.13 | 2.57 | 0.2993 | 3.30 | 0.0484 |
| Achn333301 | Achn333301 | 139.62 | 69.80 | 8.13 | -1.00 | 0.5789 | -4.10 | 0.0058 |
| Achn334011 | Achn334011 | 16.76 | 12.06 | 0.57 | -0.47 | 0.8969 | -4.88 | 0.0185 |
| Achn335091 | Achn335091 | 0.08 | 3.98 | 9.82 | 5.58 | 0.0766 | 6.89 | 0.0054 |
| Achn336111 | Achn336111 | 0.55 | 0.70 | 31.29 | 0.36 | 0.9490 | 5.84 | 0.0002 |
| Achn336411 | Achn336411 | 1.35 | 0.20 | 37.24 | -2.74 | 0.4242 | 4.78 | 0.0022 |
| Achn337241 | Achn337241 | 2.68 | 0.00 | 185.37 | -14.71 | 0.4242 | 6.11 | 0.0259 |
| Achn337521 | Achn337521 | 2.02 | 9.72 | 110.71 | 2.26 | 0.4242 | 5.77 | 0.0009 |
| Achn338411 | Achn338411 | 8.69 | 4.56 | 216.48 | -0.93 | 0.7358 | 4.64 | 0.0009 |
| Achn341261 | Achn341261 | 0.47 | 0.00 | 31.16 | -12.19 | 1.0000 | 6.06 | 0.0267 |
| Achn341521 | Achn341521 | 271.26 | 1524.28 | 8551.23 | 2.49 | 0.4242 | 4.98 | 0.0108 |
| Achn342011 | Achn342011 | 0.10 | 0.69 | 13.18 | 2.76 | 0.4242 | 7.01 | 0.0013 |
| Achn342361 | Achn342361 | 4.60 | 11.78 | 58.70 | 1.36 | 0.4665 | 3.67 | 0.0251 |
| Achn343031 | Achn343031 | 0.03 | 0.00 | 6.47 | -8.12 | 1.0000 | 7.86 | 0.0004 |
| Achn343991 | Achn343991 | 1.28 | 3.26 | 96.39 | 1.35 | 0.5775 | 6.24 | 0.0000 |
| Achn344511 | Achn344511 | 3.77 | 0.47 | 130.91 | -3.00 | 0.4242 | 5.12 | 0.0059 |
| Achn344761 | Achn344761 | 1.10 | 0.97 | 82.41 | -0.18 | 0.9867 | 6.23 | 0.0034 |
| Achn345211 | Achn345211 | 0.12 | 0.32 | 11.45 | 1.44 | 1.0000 | 6.61 | 0.0037 |
| Achn346051 | Achn346051 | 5.47 | 0.96 | 63.09 | -2.52 | 0.4242 | 3.53 | 0.0245 |
| Achn346081 | Achn346081 | 0.33 | 0.00 | 20.35 | -11.71 | 1.0000 | 5.93 | 0.0010 |
| Achn346661 | Achn346661 | 0.56 | 0.33 | 30.77 | -0.76 | 0.9226 | 5.78 | 0.0462 |
| Achn347191 | Achn347191 | 0.96 | 2.81 | 27.65 | 1.55 | 0.5668 | 4.85 | 0.0152 |
| Achn348741 | Achn348741 | 7.37 | 82.61 | 126.98 | 3.49 | 0.1017 | 4.11 | 0.0172 |
| Achn349171 | Achn349171 | 6.24 | 0.48 | 0.14 | -3.70 | 0.1602 | -5.51 | 0.0214 |
| Achn349841 | Achn349841 | 1.84 | 0.00 | 113.92 | -14.16 | 0.4242 | 5.96 | 0.0182 |
| Achn349851 | Achn349851 | 3.64 | 0.56 | 194.95 | -2.71 | 0.4242 | 5.74 | 0.0003 |
| Achn350311 | Achn350311 | 0.13 | 2.28 | 14.24 | 4.17 | 0.3820 | 6.81 | 0.0022 |
| Achn352061 | Achn352061 | 0.05 | 1.23 | 4.47 | 4.68 | 0.2634 | 6.54 | 0.0094 |
| Achn353661 | Achn353661 | 187.08 | 86.20 | 5780.57 | -1.12 | 0.5127 | 4.95 | 0.0039 |
| Achn353971 | Achn353971 | 1.13 | 1.42 | 22.41 | 0.33 | 0.9488 | 4.31 | 0.0102 |
| Achn355121 | Achn355121 | 8.18 | 1.73 | 0.10 | -2.24 | 0.4242 | -6.38 | 0.0171 |
| Achn356831 | Achn356831 | 1.15 | 0.52 | 219.36 | -1.14 | 0.7602 | 7.57 | 0.0000 |
| Achn357721 | Achn357721 | 10.94 | 44.36 | 252.98 | 2.02 | 0.4242 | 4.53 | 0.0059 |
| Achn358171 | Achn358171 | 0.08 | 1.63 | 10.47 | 4.31 | 0.4143 | 6.99 | 0.0033 |
| Achn359821 | Achn359821 | 31.31 | 8.76 | 0.46 | -1.84 | 0.4242 | -6.08 | 0.0006 |
| Achn361961 | Achn361961 | 0.41 | 1.14 | 130.52 | 1.49 | 0.7587 | 8.33 | 0.0001 |
| Achn362981 | Achn362981 | 60.15 | 27.06 | 4.94 | -1.15 | 0.4946 | -3.61 | 0.0291 |
| Achn363371 | Achn363371 | 0.36 | 4.72 | 19.46 | 3.70 | 0.2255 | 5.74 | 0.0009 |
| Achn364251 | Achn364251 | 0.49 | 1.59 | 310.88 | 1.71 | 0.4947 | 9.32 | 0.0000 |
| Achn367751 | Achn367751 | 1.49 | 0.69 | 165.73 | -1.11 | 0.7648 | 6.80 | 0.0000 |
| Achn369461 | Achn369461 | 0.02 | 0.05 | 4.06 | 1.01 | 1.0000 | 7.48 | 0.0013 |
| Achn370591 | Achn370591 | 0.16 | 1.60 | 61.71 | 3.31 | 0.4242 | 8.57 | 0.0000 |
| Achn372611 | Achn372611 | 4.10 | 0.00 | 341.97 | -15.32 | 0.4242 | 6.38 | 0.0023 |
| Achn372801 | Achn372801 | 2.62 | 7.06 | 37.61 | 1.43 | 0.5236 | 3.84 | 0.0410 |
| Achn373161 | Achn373161 | 0.27 | 0.05 | 14.28 | -2.29 | 1.0000 | 5.75 | 0.0046 |
| Achn373641 | Achn373641 | 15.00 | 135.58 | 1106.32 | 3.18 | 0.1547 | 6.20 | 0.0000 |
| Achn373661 | Achn373661 | 1.99 | 3.89 | 42.66 | 0.96 | 0.7731 | 4.42 | 0.0138 |
| Achn375781 | Achn375781 | 4.11 | 1.88 | 262.21 | -1.13 | 0.6880 | 6.00 | 0.0000 |
| Achn376911 | Achn376911 | 0.61 | 0.46 | 110.45 | -0.42 | 0.9509 | 7.50 | 0.0000 |
| Achn377291 | Achn377291 | 4.78 | 10.00 | 85.80 | 1.07 | 0.6379 | 4.17 | 0.0055 |
| Achn377591 | Achn377591 | 6.74 | 18.84 | 87.37 | 1.48 | 0.4244 | 3.70 | 0.0304 |
| Achn378531 | Achn378531 | 2.77 | 1.65 | 66.14 | -0.75 | 0.8325 | 4.58 | 0.0020 |
| Achn378991 | Achn378991 | 103.65 | 316.01 | 1477.97 | 1.61 | 0.4444 | 3.83 | 0.0417 |
| Achn379311 | Achn379311 | 0.35 | 1.72 | 53.43 | 2.31 | 0.4242 | 7.27 | 0.0000 |
| Achn379901 | Achn379901 | 11.75 | 1.11 | 0.09 | -3.40 | 0.3163 | -6.99 | 0.0044 |
| Achn381811 | Achn381811 | 73.37 | 37.05 | 3.22 | -0.99 | 0.5890 | -4.51 | 0.0015 |
| Achn384171 | Achn384171 | 0.14 | 0.00 | 6.48 | -10.42 | 1.0000 | 5.56 | 0.0399 |
| Achn385591 | Achn385591 | 84.16 | 38.24 | 5.77 | -1.14 | 0.5066 | -3.87 | 0.0161 |
| Achn387691 | Achn387691 | 0.04 | 0.36 | 43.10 | 3.03 | 1.0000 | 9.93 | 0.0000 |
| Achn387711 | Achn387711 | 6.38 | 2.48 | 92.72 | -1.37 | 0.5215 | 3.86 | 0.0121 |
| Achn389791 | Achn389791 | 0.21 | 0.21 | 4.88 | 0.01 | 1.0000 | 4.56 | 0.0411 |
| Achn313551 | Achn313551 | 0.10 | 54.37 | 364.95 | 9.11 | 0.0000 | 11.86 | 0.0000 |
| Achn069511 | Achn069511 | 0.12 | 6.55 | 116.06 | 5.72 | 0.0294 | 9.86 | 0.0000 |
| Achn342471 | Achn342471 | 0.12 | 21.15 | 57.08 | 7.47 | 0.0014 | 8.90 | 0.0000 |
| Achn260601 | Achn260601 | 0.38 | 154.40 | 100.62 | 8.66 | 0.0000 | 8.04 | 0.0000 |
| Achn086221 | Achn086221 | 71.63 | 11541.30 | 18719.90 | 7.33 | 0.0000 | 8.03 | 0.0000 |
| Achn227791 | Achn227791 | 0.20 | 18.85 | 41.61 | 6.57 | 0.0091 | 7.72 | 0.0005 |
| Achn154461 | Achn154461 | 0.04 | 7.99 | 8.43 | 7.61 | 0.0009 | 7.69 | 0.0007 |
| Achn085041 | Achn085041 | 0.13 | 90.87 | 21.15 | 9.49 | 0.0000 | 7.39 | 0.0005 |
| Achn119071 | Achn119071 | 0.09 | 12.87 | 13.00 | 7.11 | 0.0032 | 7.13 | 0.0030 |
| Achn142621 | Achn142621 | 0.60 | 24.45 | 79.53 | 5.35 | 0.0004 | 7.05 | 0.0000 |
| Achn159091 | Achn159091 | 0.02 | 2.07 | 2.41 | 6.65 | 0.0094 | 6.87 | 0.0056 |
| Achn092641 | Achn092641 | 0.23 | 8.81 | 26.00 | 5.24 | 0.0025 | 6.80 | 0.0000 |
| Achn260671 | Achn260671 | 0.13 | 10.23 | 13.97 | 6.33 | 0.0025 | 6.78 | 0.0007 |
| Achn111311 | Achn111311 | 0.03 | 2.45 | 3.15 | 6.36 | 0.0177 | 6.72 | 0.0079 |
| Achn100671 | Achn100671 | 0.30 | 153.64 | 24.73 | 9.01 | 0.0000 | 6.37 | 0.0000 |
| Achn269061 | Achn269061 | 14.50 | 170.37 | 1051.22 | 3.55 | 0.0219 | 6.18 | 0.0000 |
| Achn126261 | Achn126261 | 0.23 | 38.15 | 16.87 | 7.35 | 0.0019 | 6.18 | 0.0256 |
| Achn345841 | Achn345841 | 0.03 | 6.73 | 2.16 | 7.80 | 0.0005 | 6.16 | 0.0260 |
| Achn362941 | Achn362941 | 8.12 | 194.24 | 534.46 | 4.58 | 0.0021 | 6.04 | 0.0000 |
| Achn361021 | Achn361021 | 0.44 | 124.60 | 28.28 | 8.15 | 0.0002 | 6.01 | 0.0296 |
| Achn171281 | Achn171281 | 0.70 | 75.32 | 42.08 | 6.75 | 0.0085 | 5.91 | 0.0442 |
| Achn130551 | Achn130551 | 11.65 | 231.40 | 637.88 | 4.31 | 0.0052 | 5.77 | 0.0000 |
| Achn328811 | Achn328811 | 4.82 | 155.12 | 261.67 | 5.01 | 0.0011 | 5.76 | 0.0001 |
| Achn359661 | Achn359661 | 0.77 | 87.64 | 41.02 | 6.84 | 0.0001 | 5.74 | 0.0026 |
| Achn060311 | Achn060311 | 0.64 | 15.72 | 34.11 | 4.61 | 0.0144 | 5.73 | 0.0003 |
| Achn292671 | Achn292671 | 9.81 | 0.22 | 492.81 | -5.47 | 0.0228 | 5.65 | 0.0000 |
| Achn304301 | Achn304301 | 6.65 | 454.18 | 334.08 | 6.09 | 0.0000 | 5.65 | 0.0002 |
| Achn131421 | Achn131421 | 2.23 | 36.83 | 109.41 | 4.04 | 0.0259 | 5.61 | 0.0001 |
| Achn315151 | Achn315151 | 0.61 | 72.45 | 27.48 | 6.89 | 0.0000 | 5.49 | 0.0004 |
| Achn191341 | Achn191341 | 0.19 | 17.11 | 6.84 | 6.49 | 0.0002 | 5.17 | 0.0135 |
| Achn281461 | Achn281461 | 1.29 | 311.83 | 42.53 | 7.92 | 0.0000 | 5.05 | 0.0012 |
| Achn169421 | Achn169421 | 1.05 | 518.30 | 33.36 | 8.95 | 0.0000 | 4.99 | 0.0057 |
| Achn209941 | Achn209941 | 17.89 | 225.03 | 560.87 | 3.65 | 0.0178 | 4.97 | 0.0002 |
| Achn325961 | Achn325961 | 3.00 | 306.48 | 89.09 | 6.68 | 0.0000 | 4.89 | 0.0019 |
| Achn353821 | Achn353821 | 8.73 | 118.26 | 241.69 | 3.76 | 0.0256 | 4.79 | 0.0008 |
| Achn141771 | Achn141771 | 10.16 | 155.66 | 270.29 | 3.94 | 0.0080 | 4.73 | 0.0005 |
| Achn276401 | Achn276401 | 0.20 | 10.21 | 5.31 | 5.65 | 0.0035 | 4.71 | 0.0435 |
| Achn294421 | Achn294421 | 2.84 | 40.28 | 63.16 | 3.83 | 0.0228 | 4.47 | 0.0026 |
| Achn378601 | Achn378601 | 66.71 | 3421.91 | 1388.42 | 5.68 | 0.0001 | 4.38 | 0.0142 |
| Achn054961 | Achn054961 | 3.13 | 158.65 | 64.32 | 5.66 | 0.0001 | 4.36 | 0.0069 |
| Achn020601 | Achn020601 | 1.79 | 31.04 | 33.36 | 4.12 | 0.0177 | 4.22 | 0.0125 |
| Achn341891 | Achn341891 | 1.53 | 73.88 | 26.97 | 5.60 | 0.0005 | 4.14 | 0.0475 |
| Achn374391 | Achn374391 | 1.94 | 34.33 | 29.99 | 4.14 | 0.0138 | 3.95 | 0.0248 |
| Achn366681 | Achn366681 | 67.32 | 817.61 | 994.83 | 3.60 | 0.0170 | 3.89 | 0.0072 |
| Achn182861 | Achn182861 | 21.35 | 236.65 | 311.44 | 3.47 | 0.0321 | 3.87 | 0.0094 |
| Achn015131 | Achn015131 | 1.94 | 24.01 | 27.14 | 3.63 | 0.0289 | 3.80 | 0.0168 |
| Achn026311 | Achn026311 | 5.44 | 104.17 | 72.32 | 4.26 | 0.0033 | 3.73 | 0.0192 |
| Achn143751 | Achn143751 | 40.74 | 678.36 | 525.83 | 4.06 | 0.0048 | 3.69 | 0.0150 |
| Achn223051 | Achn223051 | 33.48 | 606.08 | 380.45 | 4.18 | 0.0031 | 3.51 | 0.0244 |
| Achn314301 | Achn314301 | 7.28 | 113.82 | 74.68 | 3.97 | 0.0065 | 3.36 | 0.0442 |
| Achn123601 | Achn123601 | 12.12 | 0.37 | 0.86 | -5.01 | 0.0036 | -3.82 | 0.0467 |
| Achn084101 | Achn084101 | 295.56 | 23.68 | 20.27 | -3.64 | 0.0191 | -3.87 | 0.0094 |
| Achn141711 | Achn141711 | 6611.88 | 404.45 | 410.23 | -4.03 | 0.0331 | -4.01 | 0.0356 |
| Achn203261 | Achn203261 | 91.74 | 1.37 | 5.52 | -6.07 | 0.0000 | -4.05 | 0.0090 |
| Achn095551 | Achn095551 | 48.39 | 1709.33 | 2.78 | 5.14 | 0.0001 | -4.12 | 0.0227 |
| Achn012091 | Achn012091 | 95.69 | 7.75 | 5.16 | -3.63 | 0.0245 | -4.21 | 0.0046 |
| Achn282341 | Achn282341 | 11.69 | 379.14 | 0.57 | 5.02 | 0.0002 | -4.35 | 0.0377 |
| Achn124951 | Achn124951 | 45.98 | 2.35 | 1.99 | -4.29 | 0.0113 | -4.53 | 0.0057 |
| Achn347891 | Achn347891 | 279.56 | 28.75 | 10.89 | -3.28 | 0.0498 | -4.68 | 0.0008 |
| Achn105301 | Achn105301 | 54.25 | 1.54 | 1.57 | -5.14 | 0.0015 | -5.11 | 0.0014 |
| Achn289831 | Achn289831 | 11.42 | 0.32 | 0.29 | -5.17 | 0.0070 | -5.30 | 0.0049 |
| Achn140301 | Achn140301 | 4.63 | 146.73 | 0.06 | 4.99 | 0.0005 | -6.20 | 0.0245 |
| Achn372361 | Achn372361 | 255.99 | 19.25 | 3.47 | -3.73 | 0.0120 | -6.21 | 0.0000 |
| Achn017741 | Achn017741 | 12.20 | 0.13 | 0.08 | -6.51 | 0.0048 | -7.20 | 0.0021 |
| Novel_000168 | XLOC_004323 | 0.77 | 1.12 | 47.40 | 0.54 | 0.8954 | 5.94 | 0.0000 |
| Novel_000169 | XLOC_004323 | 2.01 | 4.48 | 52.93 | 1.16 | 0.5741 | 4.72 | 0.0015 |
| Novel_000235 | XLOC_005912 | 47.06 | 366.88 | 1.01 | 2.96 | 0.1759 | -5.54 | 0.0258 |
| Novel_000343 | XLOC_009112 | 0.76 | 0.64 | 20.38 | -0.24 | 0.9706 | 4.75 | 0.0062 |
| Novel_000368 | XLOC_009894 | 0.69 | 0.80 | 52.85 | 0.23 | 0.9773 | 6.27 | 0.0004 |
| Novel_000390 | XLOC_010411 | 8.04 | 1.15 | 0.28 | -2.80 | 0.4242 | -4.83 | 0.0418 |
| Novel_000448 | XLOC_012937 | 0.81 | 0.00 | 15.81 | -12.98 | 0.4242 | 4.29 | 0.0251 |
| Novel_000738 | XLOC_021025 | 0.58 | 0.85 | 33.15 | 0.56 | 0.9334 | 5.84 | 0.0065 |
| Novel_000742 | XLOC_021157 | 0.64 | 1.23 | 103.73 | 0.93 | 0.7952 | 7.33 | 0.0000 |
| Novel_000786 | XLOC_022590 | 0.77 | 5.81 | 58.49 | 2.91 | 0.4242 | 6.25 | 0.0000 |
| Novel_000971 | XLOC_028595 | 1.49 | 0.16 | 53.08 | -3.25 | 0.4242 | 5.15 | 0.0005 |
| Novel_001113 | XLOC_031114 | 3.84 | 0.12 | 0.11 | -4.96 | 0.0652 | -5.13 | 0.0426 |
| Novel_001135 | XLOC_031656 | 0.12 | 0.00 | 88.35 | -10.24 | 1.0000 | 9.52 | 0.0000 |
| Novel_001339 | XLOC_038300 | 1.37 | 3.20 | 43.53 | 1.22 | 0.7240 | 4.99 | 0.0177 |
| Novel_002086 | XLOC_057792 | 0.14 | 0.73 | 9.87 | 2.35 | 0.4242 | 6.10 | 0.0125 |
| Novel_002192 | XLOC_061483 | 3.12 | 5.94 | 729.42 | 0.93 | 0.7837 | 7.87 | 0.0000 |
| Novel_002546 | XLOC_068344 | 1.28 | 0.10 | 629.41 | -3.74 | 0.4242 | 8.94 | 0.0000 |
| Novel_002547 | XLOC_068347 | 1.67 | 0.41 | 2475.88 | -2.02 | 0.5688 | 10.53 | 0.0000 |
| Novel_002661 | XLOC_072215 | 19.68 | 4.85 | 1.28 | -2.02 | 0.4242 | -3.94 | 0.0376 |
| Novel_002684 | XLOC_072337 | 0.05 | 0.97 | 22.58 | 4.35 | 0.4242 | 8.89 | 0.0000 |
| Novel_002781 | XLOC_074327 | 10.81 | 51.27 | 117.02 | 2.25 | 0.4242 | 3.44 | 0.0460 |
| Novel_002934 | XLOC_077658 | 0.36 | 0.25 | 17.87 | -0.49 | 1.0000 | 5.65 | 0.0333 |
| Novel_002953 | XLOC_078261 | 74.71 | 154.36 | 3.43 | 1.05 | 0.5471 | -4.44 | 0.0051 |
| Novel_002973 | XLOC_078857 | 136.20 | 15.00 | 5.58 | -3.18 | 0.0984 | -4.61 | 0.0019 |
| Novel_002977 | XLOC_078857 | 174.87 | 19.12 | 7.94 | -3.19 | 0.0914 | -4.46 | 0.0051 |
| Novel_003158 | XLOC_080629 | 29.67 | 8.76 | 1.61 | -1.76 | 0.4242 | -4.21 | 0.0258 |
| Novel_003186 | XLOC_082065 | 0.08 | 0.27 | 33.07 | 1.65 | 1.0000 | 8.61 | 0.0001 |
| Novel_003251 | XLOC_083838 | 0.36 | 0.16 | 10.31 | -1.15 | 1.0000 | 4.85 | 0.0196 |
| Novel_003414 | XLOC_088679 | 120.65 | 14.10 | 3.35 | -3.10 | 0.1576 | -5.17 | 0.0010 |
| Novel_003450 | XLOC_089887 | 0.17 | 0.00 | 14.92 | -10.74 | 1.0000 | 6.44 | 0.0119 |
| Novel_003491 | XLOC_090806 | 0.24 | 0.63 | 9.96 | 1.37 | 0.7165 | 5.37 | 0.0106 |
| Novel_003522 | XLOC_092134 | 17.94 | 15.60 | 0.83 | -0.20 | 0.9653 | -4.44 | 0.0242 |
| Novel_003688 | XLOC_095076 | 0.16 | 0.74 | 16.98 | 2.23 | 0.5111 | 6.75 | 0.0075 |
| Novel_003894 | XLOC_099594 | 0.22 | 5.94 | 1092.95 | 4.76 | 0.2668 | 12.29 | 0.0000 |
| Novel_003913 | XLOC_100029 | 0.20 | 0.32 | 25.59 | 0.70 | 1.0000 | 7.02 | 0.0001 |
| Novel_003921 | XLOC_100293 | 0.09 | 0.00 | 11.94 | -9.86 | 1.0000 | 7.00 | 0.0041 |
| Novel_003991 | XLOC_101797 | 0.29 | 0.95 | 12.63 | 1.72 | 0.5773 | 5.46 | 0.0087 |
| Novel_004100 | XLOC_104152 | 24.51 | 142.51 | 905.52 | 2.54 | 0.4242 | 5.21 | 0.0003 |
| Novel_004134 | XLOC_104786 | 3.87 | 1.57 | 0.04 | -1.30 | 0.5846 | -6.54 | 0.0121 |
| Novel_004466 | XLOC_111659 | 0.06 | 0.00 | 3.59 | -9.16 | 1.0000 | 5.97 | 0.0379 |
| Novel_004535 | XLOC_114181 | 1.14 | 0.10 | 36.90 | -3.55 | 0.4242 | 5.01 | 0.0049 |
| Novel_004580 | XLOC_115705 | 15.21 | 8.44 | 433.28 | -0.85 | 0.7727 | 4.83 | 0.0004 |
| Novel_004641 | XLOC_117889 | 1.20 | 1.36 | 85.12 | 0.18 | 0.9789 | 6.15 | 0.0000 |
| Novel_005006 | XLOC_129156 | 476.45 | 304.35 | 37.25 | -0.65 | 0.8033 | -3.68 | 0.0134 |
| Novel_005023 | XLOC_129838 | 0.35 | 0.79 | 19.86 | 1.18 | 0.7534 | 5.83 | 0.0011 |
| Novel_005024 | XLOC_129838 | 0.49 | 0.18 | 11.23 | -1.47 | 1.0000 | 4.51 | 0.0342 |
| Novel_005076 | XLOC_130654 | 0.52 | 5.16 | 15.68 | 3.30 | 0.4242 | 4.90 | 0.0208 |
| Novel_005534 | XLOC_140001 | 0.96 | 2.69 | 39.48 | 1.49 | 0.6436 | 5.36 | 0.0045 |
| Novel_005549 | XLOC_140098 | 0.16 | 1.17 | 4.57 | 2.87 | 0.4242 | 4.84 | 0.0477 |
| Novel_005637 | XLOC_141783 | 0.22 | 0.55 | 10.75 | 1.31 | 1.0000 | 5.60 | 0.0042 |
| Novel_005789 | XLOC_144702 | 1.29 | 0.00 | 70.61 | -13.65 | 0.4242 | 5.78 | 0.0071 |
| Novel_005797 | XLOC_144744 | 15.29 | 37.81 | 253.98 | 1.31 | 0.4832 | 4.05 | 0.0064 |
| Novel_005964 | XLOC_150006 | 0.09 | 1.86 | 7.99 | 4.45 | 0.4242 | 6.55 | 0.0134 |
| Novel_006101 | XLOC_154311 | 0.36 | 8.05 | 81.09 | 4.47 | 0.3489 | 7.80 | 0.0004 |
| Novel_006137 | XLOC_155546 | 1.54 | 18.13 | 279.59 | 3.56 | 0.0831 | 7.51 | 0.0000 |
| Novel_006148 | XLOC_155879 | 0.70 | 0.00 | 43.23 | -12.78 | 0.4242 | 5.94 | 0.0011 |
| Novel_006149 | XLOC_155879 | 2.44 | 0.00 | 155.11 | -14.57 | 0.4242 | 5.99 | 0.0001 |
| Novel_006150 | XLOC_155879 | 0.94 | 0.00 | 50.57 | -9.59 | 0.9945 | 5.75 | 0.0285 |
| Novel_006328 | XLOC_160660 | 15.50 | 2.40 | 0.62 | -2.69 | 0.4242 | -4.64 | 0.0277 |
| Novel_006467 | XLOC_167256 | 0.34 | 1.60 | 14.28 | 2.23 | 0.4547 | 5.38 | 0.0371 |
| Novel_006648 | XLOC_171346 | 12.13 | 2.83 | 0.79 | -2.10 | 0.4242 | -3.94 | 0.0430 |
| Novel_006772 | XLOC_174276 | 18.08 | 19.10 | 219.86 | 0.08 | 0.9902 | 3.60 | 0.0422 |
| Novel_006799 | XLOC_175377 | 2.33 | 2.43 | 135.32 | 0.06 | 0.9942 | 5.86 | 0.0000 |
| Novel_006837 | XLOC_175789 | 0.04 | 0.32 | 1.93 | 3.05 | 1.0000 | 5.66 | 0.0279 |
| Novel_006866 | XLOC_176349 | 0.06 | 0.26 | 17.80 | 2.04 | 1.0000 | 8.14 | 0.0002 |
| Novel_006906 | XLOC_177495 | 2.01 | 0.54 | 37.40 | -1.89 | 0.4500 | 4.22 | 0.0193 |
| Novel_006954 | XLOC_178671 | 30.03 | 48.55 | 307.50 | 0.69 | 0.8015 | 3.36 | 0.0430 |
| Novel_007005 | XLOC_179121 | 149.11 | 29.40 | 7.74 | -2.34 | 0.4242 | -4.27 | 0.0078 |
| Novel_007329 | XLOC_187092 | 2.45 | 1.73 | 37.86 | -0.50 | 0.8996 | 3.95 | 0.0118 |
| Novel_008003 | XLOC_208916 | 6.29 | 1.50 | 208.63 | -2.07 | 0.4242 | 5.05 | 0.0004 |
| Novel_008090 | XLOC_211149 | 0.52 | 0.11 | 68.97 | -2.24 | 1.0000 | 7.05 | 0.0001 |
| Novel_008105 | XLOC_211824 | 0.15 | 0.38 | 39.78 | 1.31 | 1.0000 | 8.01 | 0.0035 |
| Novel_008315 | XLOC_217617 | 107.93 | 9.16 | 0.61 | -3.56 | 0.0832 | -7.47 | 0.0004 |
| Novel_008476 | XLOC_220914 | 0.66 | 1.65 | 15.22 | 1.32 | 0.6809 | 4.53 | 0.0265 |
| Novel_008675 | XLOC_226592 | 15.30 | 51.77 | 0.84 | 1.76 | 0.4242 | -4.19 | 0.0320 |
| Novel_008941 | XLOC_234255 | 0.58 | 0.00 | 32.85 | -12.50 | 0.4242 | 5.82 | 0.0023 |
| Novel_009254 | XLOC_241462 | 1.17 | 2.73 | 25.05 | 1.22 | 0.6557 | 4.42 | 0.0131 |
| Novel_009498 | XLOC_247319 | 0.30 | 0.60 | 21.62 | 1.00 | 0.8538 | 6.17 | 0.0084 |
| Novel_009604 | XLOC_250081 | 0.99 | 0.27 | 24.89 | -1.89 | 0.4408 | 4.65 | 0.0042 |
| Novel_010281 | XLOC_268937 | 0.69 | 0.65 | 18.58 | -0.09 | 0.9927 | 4.74 | 0.0121 |
| Novel_010286 | XLOC_269190 | 11.68 | 1.94 | 0.08 | -2.59 | 0.4242 | -7.12 | 0.0002 |
| Novel_010293 | XLOC_269643 | 28.65 | 9.85 | 287.63 | -1.54 | 0.4242 | 3.33 | 0.0434 |
| Novel_010574 | XLOC_275537 | 1.42 | 0.12 | 38.85 | -3.62 | 0.4242 | 4.77 | 0.0100 |
| Novel_010671 | XLOC_277847 | 1.18 | 0.00 | 120.12 | -13.52 | 0.4242 | 6.68 | 0.0096 |
| Novel_010804 | XLOC_281273 | 0.10 | 0.11 | 7.22 | 0.08 | 1.0000 | 6.13 | 0.0274 |
| Novel_010876 | XLOC_282859 | 7.79 | 0.60 | 0.21 | -3.69 | 0.3978 | -5.19 | 0.0418 |
| Novel_010981 | XLOC_286071 | 10.03 | 7.29 | 149.11 | -0.46 | 0.9183 | 3.89 | 0.0244 |
| Novel_010999 | XLOC_286490 | 7.06 | 2.49 | 0.34 | -1.50 | 0.4297 | -4.36 | 0.0424 |
| Novel_011033 | XLOC_287244 | 0.27 | 0.06 | 34.00 | -2.15 | 1.0000 | 6.99 | 0.0000 |
| Novel_011156 | XLOC_289280 | 0.76 | 0.00 | 27.28 | -12.90 | 0.4242 | 5.16 | 0.0103 |
| Novel_011377 | XLOC_292460 | 15.63 | 2.11 | 294.11 | -2.89 | 0.4242 | 4.23 | 0.0053 |
| Novel_011482 | XLOC_294642 | 0.92 | 0.78 | 49.79 | -0.23 | 0.9745 | 5.76 | 0.0005 |
| Novel_011515 | XLOC_295615 | 16.21 | 18.40 | 0.38 | 0.18 | 0.9711 | -5.43 | 0.0254 |
| Novel_011544 | XLOC_296238 | 0.71 | 0.63 | 12.14 | -0.17 | 0.9801 | 4.10 | 0.0292 |
| Novel_011545 | XLOC_296238 | 0.92 | 0.84 | 19.17 | -0.15 | 0.9844 | 4.37 | 0.0176 |
| Novel_011547 | XLOC_296238 | 0.27 | 0.00 | 20.88 | -11.40 | 1.0000 | 6.27 | 0.0223 |
| Novel_011563 | XLOC_296340 | 0.11 | 0.00 | 21.49 | -10.11 | 1.0000 | 7.60 | 0.0028 |
| Novel_011755 | XLOC_303998 | 0.08 | 0.26 | 9.93 | 1.64 | 1.0000 | 6.92 | 0.0050 |
| Novel_011879 | XLOC_307155 | 8.80 | 8.23 | 196.50 | -0.10 | 0.9879 | 4.48 | 0.0014 |
| Novel_011896 | XLOC_307803 | 0.06 | 0.00 | 11.29 | -9.19 | 1.0000 | 7.60 | 0.0009 |
| Novel_012165 | XLOC_314388 | 1.87 | 0.00 | 31.58 | -14.19 | 0.4242 | 4.08 | 0.0398 |
| Novel_012167 | XLOC_314388 | 0.55 | 0.07 | 32.23 | -2.96 | 1.0000 | 5.88 | 0.0009 |
| Novel_012610 | XLOC_325324 | 3.50 | 2.19 | 96.67 | -0.68 | 0.8710 | 4.79 | 0.0029 |
| Novel_012613 | XLOC_325324 | 0.86 | 0.00 | 17.32 | -13.08 | 0.4242 | 4.33 | 0.0377 |
| Novel_012710 | XLOC_326990 | 0.41 | 3.46 | 77.38 | 3.09 | 0.4242 | 7.58 | 0.0000 |
| Novel_012726 | XLOC_327402 | 0.54 | 0.69 | 24.25 | 0.35 | 0.9530 | 5.49 | 0.0011 |
| Novel_012861 | XLOC_329817 | 1.34 | 0.12 | 36.73 | -3.46 | 0.4242 | 4.78 | 0.0116 |
| Novel_012884 | XLOC_330161 | 2.34 | 5.36 | 51.20 | 1.19 | 0.6514 | 4.45 | 0.0081 |
| Novel_012905 | XLOC_330448 | 0.51 | 0.00 | 17.61 | -12.33 | 1.0000 | 5.10 | 0.0165 |
| Novel_012932 | XLOC_330816 | 0.06 | 0.84 | 3.97 | 3.85 | 0.4242 | 6.09 | 0.0296 |
| Novel_012978 | XLOC_331485 | 0.39 | 0.22 | 93.97 | -0.84 | 1.0000 | 7.93 | 0.0003 |
| Novel_003895 | XLOC_099594 | 0.34 | 54.35 | 3460.44 | 7.31 | 0.0000 | 13.31 | 0.0000 |
| Novel_005077 | XLOC_130654 | 0.10 | 7.41 | 15.67 | 6.24 | 0.0387 | 7.32 | 0.0051 |
| Novel_008987 | XLOC_234513 | 0.57 | 89.46 | 69.10 | 7.30 | 0.0000 | 6.92 | 0.0000 |
| Novel_005442 | XLOC_139500 | 0.12 | 20.86 | 11.84 | 7.48 | 0.0010 | 6.66 | 0.0073 |
| Novel_003397 | XLOC_088497 | 0.74 | 22.48 | 61.75 | 4.92 | 0.0064 | 6.38 | 0.0000 |
| Novel_006350 | XLOC_161714 | 10.36 | 0.22 | 826.27 | -5.55 | 0.0283 | 6.32 | 0.0000 |
| Novel_004948 | XLOC_127388 | 0.76 | 105.66 | 56.40 | 7.11 | 0.0002 | 6.21 | 0.0035 |
| Novel_005796 | XLOC_144731 | 0.40 | 530.48 | 26.12 | 10.37 | 0.0000 | 6.02 | 0.0044 |
| Novel_006336 | XLOC_160937 | 0.08 | 19.50 | 5.16 | 7.92 | 0.0003 | 6.00 | 0.0296 |
| Novel_005795 | XLOC_144731 | 0.12 | 296.54 | 7.84 | 11.22 | 0.0000 | 5.98 | 0.0448 |
| Novel_000162 | XLOC_004140 | 0.69 | 22.47 | 37.18 | 5.03 | 0.0307 | 5.75 | 0.0032 |
| Novel_002253 | XLOC_062924 | 3.30 | 0.03 | 149.72 | -6.75 | 0.0074 | 5.50 | 0.0000 |
| Novel_005793 | XLOC_144731 | 0.87 | 673.58 | 34.47 | 9.60 | 0.0000 | 5.32 | 0.0307 |
| Novel_008682 | XLOC_226638 | 0.33 | 7.88 | 11.92 | 4.59 | 0.0194 | 5.19 | 0.0030 |
| Novel_007499 | XLOC_191562 | 0.41 | 40.12 | 10.98 | 6.62 | 0.0001 | 4.75 | 0.0396 |
| Novel_007541 | XLOC_194502 | 1.01 | 162.41 | 19.56 | 7.33 | 0.0000 | 4.27 | 0.0335 |
| Novel_002224 | XLOC_061996 | 12.60 | 187.96 | 0.61 | 3.90 | 0.0068 | -4.37 | 0.0167 |
| Novel_001474 | XLOC_040882 | 61.83 | 928.14 | 1.14 | 3.91 | 0.0075 | -5.76 | 0.0002 |
| Novel_011325 | XLOC_291596 | 60.09 | 783.74 | 0.91 | 3.71 | 0.0282 | -6.04 | 0.0002 |
