# Supplementary material for: Integrated analysis of lncRNA and mRNA transcriptomes reveals the potential regulatory role of lncRNA in kiwifruit ripening and softening
Source: Sci Rep. 2021 Jan 18;11:1671. doi: 10.1038/s41598-021-81155-1 (PMC7814023; doi:10.1038/s41598-021-81155-1)
Supplement: Supplementary file 6 — Supplementary Table S4. [file 41598_2021_81155_MOESM6_ESM.doc]

**Table S4. Differentially expressed lncRNAs in RT vs CK**

| **Transcript ID** | **Gene ID** | **FPKM (CK)** | **FPKM (RT)** | **FPKM (ABA)** | **log_2_ FPKM (RTvsCK)** | **corrected *P* value (RTvsCK)** | **log_2_ FPKM (ABAvsCK)** | **corrected *P* value (ABAvsCK)** |
| --- | --- | --- | --- | --- | --- | --- | --- | --- |
| TCONS_00002590 | XLOC_004352 | 33.69 | 0.74 | 64.76 | -5.51 | 0.0003 | 0.94 | 0.6189 |
| TCONS_00025726 | XLOC_012434 | 3993.49 | 139.44 | 5457.80 | -4.84 | 0.0097 | 0.45 | 0.8866 |
| TCONS_00038783 | XLOC_017631 | 11.89 | 0.46 | 15.90 | -4.69 | 0.0457 | 0.42 | 0.9131 |
| TCONS_00041396 | XLOC_021946 | 7.82 | 191.86 | 21.07 | 4.62 | 0.0020 | 1.43 | 0.4780 |
| TCONS_00058615 | XLOC_019298 | 0.15 | 10.76 | 0.14 | 6.18 | 0.0257 | -0.04 | 1.0000 |
| TCONS_00060874 | XLOC_026439 | 7.29 | 0.34 | 13.20 | -4.41 | 0.0420 | 0.86 | 0.7227 |
| TCONS_00068459 | XLOC_030297 | 616.87 | 58.49 | 70.54 | -3.40 | 0.0389 | -3.13 | 0.0832 |
| TCONS_00105947 | XLOC_048497 | 19.01 | 0.08 | 32.07 | -7.88 | 0.0003 | 0.75 | 0.7501 |
| TCONS_00120135 | XLOC_052437 | 105.80 | 1.44 | 97.09 | -6.20 | 0.0094 | -0.12 | 0.9819 |
| TCONS_00123703 | XLOC_058588 | 15.67 | 0.15 | 10.82 | -6.72 | 0.0063 | -0.53 | 0.8780 |
| TCONS_00124705 | XLOC_060185 | 26.13 | 0.29 | 13.68 | -6.49 | 0.0107 | -0.93 | 0.7196 |
| TCONS_00144284 | XLOC_059182 | 5.15 | 140.32 | 56.47 | 4.77 | 0.0016 | 3.46 | 0.0964 |
| TCONS_00151780 | XLOC_063098 | 32.95 | 1.32 | 30.43 | -4.65 | 0.0126 | -0.11 | 0.9830 |
| TCONS_00154589 | XLOC_064445 | 0.14 | 10.38 | 0.00 | 6.23 | 0.0074 | -15.74 | 1.0000 |
| TCONS_00161004 | XLOC_069431 | 82.27 | 0.80 | 59.70 | -6.69 | 0.0029 | -0.46 | 0.8936 |
| TCONS_00162166 | XLOC_071394 | 3.87 | 0.03 | 4.40 | -7.19 | 0.0021 | 0.18 | 0.9684 |
| TCONS_00163836 | XLOC_074404 | 24.50 | 0.09 | 40.98 | -8.15 | 0.0002 | 0.74 | 0.7719 |
| TCONS_00185173 | XLOC_076293 | 17.09 | 0.28 | 85.61 | -5.95 | 0.0026 | 2.32 | 0.4242 |
| TCONS_00186529 | XLOC_079716 | 26.05 | 0.11 | 23.67 | -7.94 | 0.0004 | -0.14 | 0.9791 |
| TCONS_00190701 | XLOC_075616 | 0.18 | 8.18 | 0.05 | 5.51 | 0.0037 | -1.90 | 1.0000 |
| TCONS_00200405 | XLOC_083442 | 9.56 | 2444.40 | 7.22 | 8.00 | 0.0009 | -0.41 | 0.9676 |
| TCONS_00216592 | XLOC_094105 | 13.10 | 0.14 | 25.78 | -6.56 | 0.0113 | 0.98 | 0.6967 |
| TCONS_00218562 | XLOC_097492 | 35.16 | 0.13 | 54.44 | -8.04 | 0.0003 | 0.63 | 0.8269 |
| TCONS_00224819 | XLOC_094532 | 12.91 | 0.09 | 50.72 | -7.17 | 0.0022 | 1.97 | 0.4242 |
| TCONS_00257815 | XLOC_111091 | 31.90 | 0.60 | 16.10 | -5.73 | 0.0277 | -0.99 | 0.6992 |
| TCONS_00266486 | XLOC_114258 | 16.47 | 0.53 | 1.44 | -4.97 | 0.0352 | -3.52 | 0.2055 |
| TCONS_00274948 | XLOC_113735 | 152.17 | 2.51 | 163.45 | -5.92 | 0.0363 | 0.10 | 0.9876 |
| TCONS_00275331 | XLOC_115022 | 18.48 | 0.18 | 44.09 | -6.69 | 0.0083 | 1.25 | 0.5395 |
| TCONS_00278138 | XLOC_119631 | 1294.06 | 35.34 | 2853.22 | -5.19 | 0.0012 | 1.14 | 0.4953 |
| TCONS_00288617 | XLOC_116277 | 1.46 | 120.76 | 5.29 | 6.37 | 0.0073 | 1.86 | 0.5951 |
| TCONS_00293626 | XLOC_122827 | 30.19 | 0.33 | 50.44 | -6.52 | 0.0119 | 0.74 | 0.8089 |
| TCONS_00295100 | XLOC_125321 | 19.24 | 0.15 | 21.31 | -6.99 | 0.0034 | 0.15 | 0.9767 |
| TCONS_00295439 | XLOC_125856 | 16.49 | 0.21 | 10.86 | -6.33 | 0.0152 | -0.60 | 0.8618 |
| TCONS_00299670 | XLOC_132572 | 11.99 | 0.05 | 2.82 | -7.93 | 0.0004 | -2.09 | 0.4242 |
| TCONS_00308273 | XLOC_125247 | 14.68 | 0.44 | 14.53 | -5.06 | 0.0047 | -0.01 | 0.9987 |
| TCONS_00309834 | XLOC_128950 | 0.10 | 45.33 | 0.00 | 8.78 | 0.0000 | -10.01 | 1.0000 |
| TCONS_00311718 | XLOC_133585 | 219.77 | 6.46 | 179.08 | -5.09 | 0.0001 | -0.30 | 0.9393 |
| TCONS_00313535 | XLOC_124936 | 42.32 | 0.60 | 27.22 | -6.14 | 0.0002 | -0.64 | 0.8137 |
| TCONS_00320209 | XLOC_128953 | 0.11 | 9.48 | 0.00 | 6.47 | 0.0114 | -10.06 | 1.0000 |
| TCONS_00332269 | XLOC_144185 | 37.94 | 0.97 | 44.89 | -5.29 | 0.0326 | 0.24 | 0.9579 |
| TCONS_00353279 | XLOC_147544 | 9.61 | 0.12 | 5.59 | -6.37 | 0.0022 | -0.78 | 0.7661 |
| TCONS_00354149 | XLOC_148918 | 13.96 | 0.21 | 9.56 | -6.08 | 0.0301 | -0.55 | 0.8936 |
| TCONS_00354568 | XLOC_149702 | 3.61 | 74.13 | 22.14 | 4.36 | 0.0191 | 2.62 | 0.4242 |
| TCONS_00355840 | XLOC_151779 | 5.51 | 0.09 | 3.00 | -5.96 | 0.0385 | -0.88 | 0.7880 |
| TCONS_00355940 | XLOC_151955 | 29.68 | 0.15 | 6.36 | -7.60 | 0.0009 | -2.22 | 0.4242 |
| TCONS_00360277 | XLOC_148069 | 96.49 | 0.12 | 92.83 | -9.62 | 0.0020 | -0.06 | 0.9926 |
| TCONS_00371425 | XLOC_156078 | 31.97 | 0.25 | 17.53 | -6.97 | 0.0035 | -0.87 | 0.7359 |
| TCONS_00396703 | XLOC_168392 | 55.33 | 0.22 | 8.55 | -7.95 | 0.0003 | -2.69 | 0.3827 |
| TCONS_00407664 | XLOC_169687 | 758.32 | 41.75 | 683.93 | -4.18 | 0.0028 | -0.15 | 0.9746 |
| TCONS_00430152 | XLOC_179433 | 3.45 | 0.04 | 5.16 | -6.38 | 0.0167 | 0.58 | 0.8738 |
| TCONS_00432556 | XLOC_183409 | 0.19 | 6.36 | 1.48 | 5.05 | 0.0031 | 2.95 | 0.4242 |
| TCONS_00432944 | XLOC_183970 | 278.07 | 0.15 | 225.47 | -10.83 | 0.0002 | -0.30 | 0.9322 |
| TCONS_00447297 | XLOC_187376 | 16.38 | 0.10 | 52.61 | -7.43 | 0.0011 | 1.68 | 0.4242 |
| TCONS_00453258 | XLOC_197160 | 2.14 | 0.01 | 6.74 | -7.49 | 0.0012 | 1.66 | 0.4242 |
| TCONS_00453288 | XLOC_197202 | 108.63 | 4.99 | 165.81 | -4.44 | 0.0219 | 0.61 | 0.8307 |
| TCONS_00474976 | XLOC_191675 | 0.24 | 11.26 | 0.00 | 5.56 | 0.0410 | -11.22 | 1.0000 |
| TCONS_00476465 | XLOC_196791 | 589.41 | 10.62 | 1929.58 | -5.80 | 0.0211 | 1.71 | 0.4242 |
| TCONS_00480058 | XLOC_203335 | 283.76 | 3497.33 | 689.17 | 3.62 | 0.0231 | 1.28 | 0.4941 |
| TCONS_00498274 | XLOC_212849 | 1414.13 | 56.58 | 3368.78 | -4.64 | 0.0077 | 1.25 | 0.4297 |
| TCONS_00498405 | XLOC_213078 | 0.65 | 52.80 | 6.71 | 6.34 | 0.0205 | 3.36 | 0.4242 |
| TCONS_00498698 | XLOC_213541 | 17.65 | 0.13 | 26.47 | -7.11 | 0.0032 | 0.58 | 0.8618 |
| TCONS_00499061 | XLOC_214104 | 11.18 | 0.25 | 12.77 | -5.46 | 0.0457 | 0.19 | 0.9699 |
| TCONS_00499395 | XLOC_214616 | 9.48 | 0.10 | 12.92 | -6.57 | 0.0004 | 0.45 | 0.8901 |
| TCONS_00506013 | XLOC_210736 | 12.99 | 0.16 | 51.16 | -6.33 | 0.0185 | 1.98 | 0.4242 |
| TCONS_00518923 | XLOC_216800 | 24.23 | 0.40 | 48.66 | -5.93 | 0.0336 | 1.01 | 0.6640 |
| TCONS_00524470 | XLOC_216039 | 2.73 | 70.27 | 0.23 | 4.69 | 0.0155 | -3.58 | 0.4242 |
| TCONS_00526446 | XLOC_220891 | 14.44 | 0.14 | 6.21 | -6.65 | 0.0074 | -1.22 | 0.5653 |
| TCONS_00543546 | XLOC_227086 | 3.52 | 0.13 | 18.00 | -4.73 | 0.0425 | 2.35 | 0.4242 |
| TCONS_00547683 | XLOC_233901 | 135.68 | 1.45 | 126.36 | -6.55 | 0.0004 | -0.10 | 0.9845 |
| TCONS_00553504 | XLOC_228085 | 0.02 | 4.15 | 0.34 | 7.67 | 0.0008 | 4.08 | 1.0000 |
| TCONS_00564965 | XLOC_230716 | 4659.88 | 3.80 | 12666.80 | -10.26 | 0.0000 | 1.44 | 0.4540 |
| TCONS_00568454 | XLOC_238966 | 2794.62 | 272.84 | 363.56 | -3.36 | 0.0394 | -2.94 | 0.1177 |
| TCONS_00573030 | XLOC_246551 | 4.17 | 0.07 | 4.58 | -5.95 | 0.0389 | 0.14 | 0.9831 |
| TCONS_00584104 | XLOC_239381 | 0.43 | 91.59 | 3.65 | 7.75 | 0.0007 | 3.10 | 0.4242 |
| TCONS_00594345 | XLOC_247411 | 121.44 | 1.69 | 108.19 | -6.17 | 0.0000 | -0.17 | 0.9688 |
| TCONS_00595956 | XLOC_250182 | 80.48 | 0.22 | 114.71 | -8.52 | 0.0126 | 0.51 | 0.8688 |
| TCONS_00596015 | XLOC_250318 | 1.90 | 139.98 | 38.72 | 6.21 | 0.0112 | 4.35 | 0.3082 |
| TCONS_00608362 | XLOC_253129 | 0.53 | 27.13 | 0.28 | 5.67 | 0.0012 | -0.93 | 1.0000 |
| TCONS_00611786 | XLOC_255681 | 1116.94 | 62.72 | 2866.90 | -4.15 | 0.0271 | 1.36 | 0.4242 |
| TCONS_00613757 | XLOC_258999 | 4.32 | 89.63 | 5.75 | 4.38 | 0.0060 | 0.41 | 0.9281 |
| TCONS_00619335 | XLOC_267968 | 20.62 | 798.99 | 351.22 | 5.28 | 0.0063 | 4.09 | 0.1148 |
| TCONS_00623440 | XLOC_274964 | 8.83 | 0.13 | 23.03 | -6.12 | 0.0237 | 1.38 | 0.4439 |
| TCONS_00633657 | XLOC_291788 | 7.27 | 0.30 | 5.19 | -4.62 | 0.0174 | -0.49 | 0.8882 |
| TCONS_00634104 | XLOC_292510 | 8.37 | 0.08 | 3.72 | -6.65 | 0.0034 | -1.17 | 0.5633 |
| TCONS_00634555 | XLOC_293280 | 43.78 | 0.31 | 32.47 | -7.14 | 0.0002 | -0.43 | 0.8966 |
| TCONS_00634790 | XLOC_293634 | 99.63 | 0.43 | 80.51 | -7.86 | 0.0000 | -0.31 | 0.9306 |
| TCONS_00634865 | XLOC_293761 | 76.61 | 0.69 | 35.86 | -6.80 | 0.0000 | -1.10 | 0.5410 |
| TCONS_00634880 | XLOC_293783 | 35.83 | 0.21 | 15.40 | -7.39 | 0.0004 | -1.22 | 0.5092 |
| TCONS_00635667 | XLOC_294962 | 1328.81 | 10.95 | 3115.60 | -6.92 | 0.0014 | 1.23 | 0.4484 |
| TCONS_00639753 | XLOC_301527 | 5.36 | 0.08 | 30.34 | -6.13 | 0.0231 | 2.50 | 0.4242 |
| TCONS_00646852 | XLOC_313285 | 4.44 | 0.08 | 5.62 | -5.77 | 0.0469 | 0.34 | 0.9386 |
| TCONS_00647122 | XLOC_313749 | 33.51 | 0.67 | 21.50 | -5.64 | 0.0002 | -0.64 | 0.8024 |
| TCONS_00648051 | XLOC_315271 | 34.41 | 0.50 | 61.19 | -6.10 | 0.0276 | 0.83 | 0.7784 |
| TCONS_00650605 | XLOC_319527 | 7.58 | 0.14 | 28.87 | -5.79 | 0.0029 | 1.93 | 0.4242 |
| TCONS_00650610 | XLOC_319535 | 3.38 | 0.02 | 8.13 | -7.67 | 0.0437 | 1.27 | 0.4846 |
| TCONS_00652140 | XLOC_322160 | 5.37 | 0.12 | 6.06 | -5.46 | 0.0488 | 0.18 | 0.9733 |
| TCONS_00652711 | XLOC_323132 | 17.72 | 0.19 | 29.11 | -6.51 | 0.0047 | 0.72 | 0.7867 |
| TCONS_00652978 | XLOC_323577 | 0.88 | 66.18 | 3.16 | 6.24 | 0.0255 | 1.85 | 0.6921 |
| TCONS_00654740 | XLOC_326524 | 1429.12 | 2.99 | 1292.66 | -8.90 | 0.0058 | -0.14 | 0.9747 |
| TCONS_00656549 | XLOC_329657 | 4.07 | 0.07 | 6.76 | -5.83 | 0.0487 | 0.73 | 0.8302 |
| TCONS_00657618 | XLOC_331496 | 15.11 | 0.31 | 7.56 | -5.60 | 0.0007 | -1.00 | 0.6211 |
| TCONS_00657667 | XLOC_331582 | 40.34 | 0.70 | 25.82 | -5.86 | 0.0000 | -0.64 | 0.7968 |
| TCONS_00657792 | XLOC_331766 | 55.07 | 0.37 | 50.27 | -7.22 | 0.0000 | -0.13 | 0.9770 |
| TCONS_00657798 | XLOC_331785 | 19.83 | 1.52 | 15.12 | -3.70 | 0.0466 | -0.39 | 0.9076 |
| TCONS_00657953 | XLOC_332042 | 10.71 | 0.42 | 6.24 | -4.67 | 0.0152 | -0.78 | 0.7708 |
| TCONS_00658000 | XLOC_332115 | 17.11 | 0.48 | 11.55 | -5.15 | 0.0030 | -0.57 | 0.8512 |
| TCONS_00658059 | XLOC_332202 | 6.65 | 1606.90 | 22.30 | 7.92 | 0.0006 | 1.75 | 0.7327 |
| TCONS_00658163 | XLOC_332360 | 26.95 | 0.52 | 25.25 | -5.70 | 0.0005 | -0.09 | 0.9862 |
| TCONS_00658165 | XLOC_332366 | 18.15 | 0.17 | 12.60 | -6.70 | 0.0009 | -0.53 | 0.8684 |
| TCONS_00658266 | XLOC_332508 | 206.11 | 0.86 | 280.60 | -7.91 | 0.0000 | 0.45 | 0.8898 |
| TCONS_00658299 | XLOC_332552 | 9.54 | 0.20 | 4.19 | -5.61 | 0.0161 | -1.19 | 0.5851 |
| TCONS_00658305 | XLOC_332561 | 20.72 | 0.59 | 16.14 | -5.14 | 0.0035 | -0.36 | 0.9215 |
| TCONS_00658335 | XLOC_332613 | 90.13 | 0.60 | 39.21 | -7.23 | 0.0000 | -1.20 | 0.4609 |
| TCONS_00658364 | XLOC_332669 | 179.44 | 1.30 | 107.06 | -7.11 | 0.0000 | -0.75 | 0.7424 |
| TCONS_00658371 | XLOC_332682 | 146.30 | 0.05 | 86.28 | -11.44 | 0.0000 | -0.76 | 0.7322 |
| TCONS_00658390 | XLOC_332708 | 104.60 | 0.61 | 112.75 | -7.41 | 0.0002 | 0.11 | 0.9831 |
| TCONS_00658397 | XLOC_332717 | 12.89 | 0.22 | 7.28 | -5.90 | 0.0078 | -0.82 | 0.7598 |
| TCONS_00658433 | XLOC_332762 | 49.66 | 3.06 | 93.43 | -4.02 | 0.0417 | 0.91 | 0.6644 |
| TCONS_00658536 | XLOC_332910 | 148.08 | 1.64 | 95.99 | -6.50 | 0.0000 | -0.63 | 0.8053 |
| TCONS_00667973 | XLOC_276979 | 63.39 | 1428.43 | 0.00 | 4.49 | 0.0266 | -19.27 | 0.4242 |
| TCONS_00689960 | XLOC_332805 | 139.86 | 0.77 | 91.57 | -7.50 | 0.0000 | -0.61 | 0.8144 |
| TCONS_00701307 | XLOC_285465 | 0.74 | 72.42 | 0.21 | 6.60 | 0.0182 | -1.84 | 0.8462 |
| TCONS_00701746 | XLOC_286624 | 186.09 | 9.10 | 26.22 | -4.35 | 0.0031 | -2.83 | 0.1939 |
| TCONS_00704083 | XLOC_293054 | 8.17 | 0.16 | 5.09 | -5.67 | 0.0140 | -0.68 | 0.8271 |
| TCONS_00708263 | XLOC_304980 | 8.04 | 0.13 | 21.14 | -5.94 | 0.0180 | 1.39 | 0.4242 |
| TCONS_00710783 | XLOC_312187 | 0.33 | 469.17 | 0.52 | 10.46 | 0.0000 | 0.64 | 0.9406 |
| TCONS_00714332 | XLOC_322602 | 12519.70 | 40.83 | 6233.33 | -8.26 | 0.0000 | -1.01 | 0.7348 |
| TCONS_00716263 | XLOC_328127 | 1.45 | 140.38 | 1.91 | 6.59 | 0.0127 | 0.39 | 0.9680 |
| TCONS_00717626 | XLOC_332025 | 5.86 | 1101.45 | 0.00 | 7.56 | 0.0002 | -15.84 | 0.4242 |
| TCONS_00730755 | XLOC_292115 | 34.18 | 1.63 | 23.10 | -4.39 | 0.0039 | -0.57 | 0.8351 |
| TCONS_00742220 | XLOC_326902 | 6.20 | 0.16 | 6.08 | -5.26 | 0.0364 | -0.03 | 0.9977 |
| TCONS_00748200 | XLOC_265437 | 0.16 | 45.89 | 0.08 | 8.18 | 0.0001 | -1.07 | 1.0000 |
| TCONS_00770147 | XLOC_331036 | 0.62 | 132.92 | 1.32 | 7.74 | 0.0000 | 1.08 | 0.7678 |
| TCONS_00774514 | XLOC_266724 | 0.40 | 8.47 | 0.41 | 4.41 | 0.0359 | 0.04 | 1.0000 |
| TCONS_00776595 | XLOC_274582 | 0.43 | 20.45 | 0.00 | 5.56 | 0.0452 | -12.08 | 1.0000 |
| TCONS_00788952 | XLOC_319050 | 6731.31 | 589.06 | 1568.99 | -3.51 | 0.0287 | -2.10 | 0.4242 |
| TCONS_00077491 | XLOC_035972 | 23.19 | 0.24 | 231.17 | -6.59 | 0.0000 | 3.32 | 0.0479 |
| TCONS_00094824 | XLOC_042139 | 3.33 | 120.91 | 52.68 | 5.18 | 0.0005 | 3.98 | 0.0280 |
| TCONS_00330037 | XLOC_140553 | 0.50 | 9.62 | 11.79 | 4.25 | 0.0438 | 4.55 | 0.0191 |
| TCONS_00376254 | XLOC_155131 | 38.15 | 0.44 | 0.13 | -6.45 | 0.0003 | -8.16 | 0.0002 |
| TCONS_00611883 | XLOC_255841 | 22.59 | 0.16 | 0.14 | -7.16 | 0.0028 | -7.31 | 0.0020 |
| TCONS_00696001 | XLOC_270586 | 119.66 | 2.33 | 1783.52 | -5.68 | 0.0000 | 3.90 | 0.0246 |
| TCONS_00781977 | XLOC_294112 | 16.92 | 1.15 | 1.10 | -3.87 | 0.0398 | -3.94 | 0.0328 |
