# Supplementary material for: Integrated analysis of lncRNA and mRNA transcriptomes reveals the potential regulatory role of lncRNA in kiwifruit ripening and softening
Source: Sci Rep. 2021 Jan 18;11:1671. doi: 10.1038/s41598-021-81155-1 (PMC7814023; doi:10.1038/s41598-021-81155-1)
Supplement: Supplementary file 7 — Supplementary Table S5. [file 41598_2021_81155_MOESM7_ESM.doc]

**Table S5. Differentially expressed lncRNAs in ABA vs CK**

| **Transcript ID** | **Gene ID** | **FPKM (CK)** | **FPKM (RT)** | **FPKM (ABA)** | **log_2_ FPKM (RTvsCK)** | **corrected *P* value**  **(RTvsCK)** | **log_2_ FPKM (ABAvsCK)** | **corrected *P* value**  **(ABAvsCK)** |
| --- | --- | --- | --- | --- | --- | --- | --- | --- |
| TCONS_00005212 | XLOC_008581 | 0.30 | 0.12 | 12.77 | -1.28 | 1.0000 | 5.40 | 0.0120 |
| TCONS_00038168 | XLOC_016730 | 0.10 | 0.00 | 13.06 | -9.97 | 1.0000 | 7.02 | 0.0011 |
| TCONS_00045079 | XLOC_020028 | 2.97 | 0.00 | 214.92 | -14.86 | 0.4242 | 6.18 | 0.0229 |
| TCONS_00059115 | XLOC_021082 | 1.26 | 3.04 | 75.29 | 1.26 | 0.8279 | 5.90 | 0.0383 |
| TCONS_00060808 | XLOC_026342 | 10785.00 | 931.59 | 554.00 | -3.53 | 0.1342 | -4.28 | 0.0208 |
| TCONS_00061538 | XLOC_027676 | 0.04 | 0.08 | 5.06 | 1.03 | 1.0000 | 6.96 | 0.0046 |
| TCONS_00078378 | XLOC_037386 | 0.33 | 0.00 | 19.44 | -11.69 | 1.0000 | 5.88 | 0.0032 |
| TCONS_00078945 | XLOC_038300 | 0.50 | 3.38 | 46.80 | 2.75 | 0.4242 | 6.54 | 0.0123 |
| TCONS_00086931 | XLOC_033014 | 0.17 | 0.00 | 11.32 | -10.74 | 1.0000 | 6.05 | 0.0326 |
| TCONS_00120565 | XLOC_053230 | 1.11 | 0.00 | 45.18 | -13.44 | 0.4242 | 5.35 | 0.0035 |
| TCONS_00130749 | XLOC_053230 | 0.11 | 0.06 | 21.75 | -0.78 | 1.0000 | 7.67 | 0.0038 |
| TCONS_00152237 | XLOC_064363 | 0.81 | 0.00 | 321.43 | -12.98 | 0.4242 | 8.64 | 0.0000 |
| TCONS_00165913 | XLOC_072094 | 3.32 | 0.00 | 572.48 | -15.02 | 0.4242 | 7.43 | 0.0001 |
| TCONS_00177447 | XLOC_075900 | 5.12 | 5.05 | 333.98 | -0.02 | 0.9992 | 6.03 | 0.0023 |
| TCONS_00179037 | XLOC_078396 | 0.07 | 0.14 | 5.63 | 1.05 | 1.0000 | 6.39 | 0.0164 |
| TCONS_00179691 | XLOC_079590 | 0.33 | 0.00 | 51.93 | -11.68 | 1.0000 | 7.31 | 0.0020 |
| TCONS_00181941 | XLOC_075904 | 0.72 | 0.00 | 76.26 | -12.82 | 0.4242 | 6.72 | 0.0002 |
| TCONS_00185025 | XLOC_075901 | 0.66 | 0.00 | 34.72 | -12.70 | 0.4242 | 5.71 | 0.0298 |
| TCONS_00185026 | XLOC_075905 | 0.27 | 1.59 | 16.70 | 2.57 | 0.4242 | 5.97 | 0.0066 |
| TCONS_00221596 | XLOC_095328 | 5.48 | 0.00 | 116.81 | -15.74 | 0.4242 | 4.41 | 0.0370 |
| TCONS_00231847 | XLOC_096653 | 10.82 | 30.61 | 169.63 | 1.50 | 0.4242 | 3.97 | 0.0070 |
| TCONS_00236559 | XLOC_100790 | 2.64 | 2.36 | 74.45 | -0.16 | 0.9859 | 4.82 | 0.0265 |
| TCONS_00237221 | XLOC_101759 | 0.21 | 0.00 | 18.01 | -11.04 | 1.0000 | 6.42 | 0.0155 |
| TCONS_00238293 | XLOC_103605 | 1.37 | 2.45 | 367.11 | 0.84 | 0.9124 | 8.07 | 0.0003 |
| TCONS_00238294 | XLOC_103608 | 0.04 | 0.00 | 797.26 | -8.68 | 1.0000 | 14.24 | 0.0000 |
| TCONS_00243799 | XLOC_099117 | 6.98 | 4.67 | 146.59 | -0.58 | 0.9147 | 4.39 | 0.0276 |
| TCONS_00248638 | XLOC_103607 | 2.11 | 21.48 | 381.78 | 3.34 | 0.4242 | 7.50 | 0.0014 |
| TCONS_00256891 | XLOC_109636 | 0.46 | 0.00 | 24.36 | -12.18 | 1.0000 | 5.72 | 0.0019 |
| TCONS_00258020 | XLOC_111413 | 0.08 | 0.00 | 10.93 | -9.69 | 1.0000 | 7.04 | 0.0037 |
| TCONS_00261562 | XLOC_110274 | 51.43 | 214.66 | 2.78 | 2.06 | 0.4242 | -4.21 | 0.0158 |
| TCONS_00278029 | XLOC_119419 | 2.59 | 0.00 | 88.65 | -14.66 | 0.4242 | 5.10 | 0.0016 |
| TCONS_00284285 | XLOC_118784 | 0.05 | 0.21 | 5.14 | 2.03 | 1.0000 | 6.63 | 0.0096 |
| TCONS_00301130 | XLOC_134961 | 1.00 | 0.00 | 115.49 | -13.29 | 0.4242 | 6.85 | 0.0006 |
| TCONS_00303201 | XLOC_126548 | 3.42 | 1.72 | 117.60 | -0.99 | 0.7922 | 5.10 | 0.0013 |
| TCONS_00311525 | XLOC_133099 | 0.18 | 0.20 | 30.91 | 0.16 | 1.0000 | 7.45 | 0.0014 |
| TCONS_00328126 | XLOC_137362 | 0.37 | 0.00 | 12.65 | -11.84 | 1.0000 | 5.11 | 0.0012 |
| TCONS_00329658 | XLOC_139925 | 1.17 | 0.00 | 23.88 | -13.52 | 0.4242 | 4.35 | 0.0360 |
| TCONS_00359159 | XLOC_152402 | 1.33 | 0.64 | 39.05 | -1.07 | 0.8430 | 4.87 | 0.0228 |
| TCONS_00362168 | XLOC_152926 | 1.29 | 0.00 | 154.44 | -13.65 | 0.4242 | 6.91 | 0.0018 |
| TCONS_00372064 | XLOC_157088 | 1.02 | 0.39 | 132.17 | -1.39 | 0.7434 | 7.02 | 0.0000 |
| TCONS_00381934 | XLOC_159292 | 0.85 | 0.00 | 47.01 | -13.05 | 0.4242 | 5.80 | 0.0006 |
| TCONS_00393714 | XLOC_163346 | 0.11 | 0.03 | 10.82 | -1.98 | 1.0000 | 6.60 | 0.0012 |
| TCONS_00407724 | XLOC_169783 | 0.34 | 0.01 | 5.58 | -4.58 | 1.0000 | 4.06 | 0.0346 |
| TCONS_00430958 | XLOC_180800 | 1.35 | 1.05 | 56.06 | -0.36 | 0.9543 | 5.38 | 0.0019 |
| TCONS_00438294 | XLOC_182813 | 73.84 | 14.12 | 2.43 | -2.39 | 0.4242 | -4.93 | 0.0385 |
| TCONS_00448734 | XLOC_189697 | 0.04 | 0.00 | 7.03 | -8.69 | 1.0000 | 7.41 | 0.0015 |
| TCONS_00464223 | XLOC_186377 | 0.37 | 0.58 | 31.83 | 0.66 | 0.8938 | 6.44 | 0.0001 |
| TCONS_00494128 | XLOC_206101 | 0.10 | 0.00 | 13.53 | -10.02 | 1.0000 | 7.02 | 0.0039 |
| TCONS_00495028 | XLOC_207617 | 0.34 | 0.09 | 21.13 | -1.90 | 1.0000 | 5.95 | 0.0001 |
| TCONS_00497192 | XLOC_211122 | 0.08 | 0.04 | 9.46 | -0.97 | 1.0000 | 6.87 | 0.0045 |
| TCONS_00512059 | XLOC_207037 | 0.26 | 0.07 | 14.77 | -1.95 | 1.0000 | 5.81 | 0.0097 |
| TCONS_00515604 | XLOC_207035 | 0.61 | 0.32 | 87.64 | -0.92 | 0.8756 | 7.16 | 0.0000 |
| TCONS_00518293 | XLOC_215776 | 0.39 | 0.00 | 107.29 | -11.94 | 1.0000 | 8.09 | 0.0003 |
| TCONS_00521729 | XLOC_221452 | 0.04 | 0.00 | 4.61 | -8.59 | 1.0000 | 6.90 | 0.0052 |
| TCONS_00522049 | XLOC_221994 | 0.23 | 0.40 | 11.48 | 0.80 | 1.0000 | 5.63 | 0.0346 |
| TCONS_00569085 | XLOC_239996 | 1.36 | 5.83 | 68.56 | 2.10 | 0.4242 | 5.65 | 0.0004 |
| TCONS_00570565 | XLOC_242597 | 1.00 | 0.00 | 430.03 | -13.28 | 0.4242 | 8.75 | 0.0000 |
| TCONS_00594487 | XLOC_247685 | 1.38 | 0.00 | 63.47 | -13.75 | 0.4242 | 5.52 | 0.0001 |
| TCONS_00611457 | XLOC_255137 | 0.28 | 0.00 | 32.80 | -11.45 | 1.0000 | 6.87 | 0.0005 |
| TCONS_00611577 | XLOC_255331 | 0.36 | 0.00 | 11.45 | -11.80 | 1.0000 | 5.01 | 0.0322 |
| TCONS_00612166 | XLOC_256356 | 0.25 | 0.00 | 28.92 | -11.28 | 1.0000 | 6.86 | 0.0059 |
| TCONS_00614566 | XLOC_260209 | 0.21 | 0.00 | 18.40 | -11.05 | 1.0000 | 6.44 | 0.0056 |
| TCONS_00620906 | XLOC_270599 | 0.27 | 0.45 | 7.82 | 0.71 | 1.0000 | 4.85 | 0.0466 |
| TCONS_00622192 | XLOC_272736 | 0.05 | 0.00 | 5.49 | -8.92 | 1.0000 | 6.83 | 0.0049 |
| TCONS_00625296 | XLOC_278105 | 1.55 | 12.20 | 97.23 | 2.97 | 0.4242 | 5.97 | 0.0334 |
| TCONS_00630015 | XLOC_285824 | 3.92 | 5.13 | 70.24 | 0.39 | 0.9410 | 4.16 | 0.0280 |
| TCONS_00635380 | XLOC_294572 | 0.07 | 0.00 | 12.18 | -9.42 | 1.0000 | 7.47 | 0.0004 |
| TCONS_00642040 | XLOC_305272 | 2.25 | 0.00 | 72.77 | -14.46 | 0.4242 | 5.02 | 0.0330 |
| TCONS_00642218 | XLOC_305592 | 0.15 | 0.00 | 9.96 | -10.54 | 1.0000 | 6.06 | 0.0264 |
| TCONS_00657725 | XLOC_331663 | 0.04 | 0.45 | 4.19 | 3.48 | 1.0000 | 6.71 | 0.0081 |
| TCONS_00664855 | XLOC_269186 | 19.04 | 2.54 | 0.25 | -2.91 | 0.4242 | -6.28 | 0.0182 |
| TCONS_00666002 | XLOC_271872 | 1.04 | 0.00 | 130.79 | -13.35 | 0.4242 | 6.97 | 0.0015 |
| TCONS_00698393 | XLOC_277289 | 0.29 | 0.00 | 28.50 | -11.48 | 1.0000 | 6.64 | 0.0076 |
| TCONS_00751512 | XLOC_275591 | 0.30 | 0.36 | 17.30 | 0.25 | 1.0000 | 5.85 | 0.0483 |
| TCONS_00077491 | XLOC_035972 | 23.19 | 0.24 | 231.17 | -6.59 | 0.0000 | 3.32 | 0.0479 |
| TCONS_00094824 | XLOC_042139 | 3.33 | 120.91 | 52.68 | 5.18 | 0.0005 | 3.98 | 0.0280 |
| TCONS_00330037 | XLOC_140553 | 0.50 | 9.62 | 11.79 | 4.25 | 0.0438 | 4.55 | 0.0191 |
| TCONS_00376254 | XLOC_155131 | 38.15 | 0.44 | 0.13 | -6.45 | 0.0003 | -8.16 | 0.0002 |
| TCONS_00611883 | XLOC_255841 | 22.59 | 0.16 | 0.14 | -7.16 | 0.0028 | -7.31 | 0.0020 |
| TCONS_00696001 | XLOC_270586 | 119.66 | 2.33 | 1783.52 | -5.68 | 0.0000 | 3.90 | 0.0246 |
| TCONS_00781977 | XLOC_294112 | 16.92 | 1.15 | 1.10 | -3.87 | 0.0398 | -3.94 | 0.0328 |
