# Supplementary material for: Integrated analysis of lncRNA and mRNA transcriptomes reveals the potential regulatory role of lncRNA in kiwifruit ripening and softening
Source: Sci Rep. 2021 Jan 18;11:1671. doi: 10.1038/s41598-021-81155-1 (PMC7814023; doi:10.1038/s41598-021-81155-1)
Supplement: Supplementary file 8 — Supplementary Table S6. [file 41598_2021_81155_MOESM8_ESM.doc]

**Table S6. KEGG analysis of DEGs in ABA vs CK**

| **KEGG pathway** | **Map ID** | **Corrected *P* value** | **Gene number** | **Gene ID** |
| --- | --- | --- | --- | --- |
| Phenylalanine metabolism | ath00360 | 0.0852 | 12 | Achn353661, Achn166351, Achn153791, Achn213291, Achn022871, Achn166341, Achn132211, Achn356831, Achn287101, Achn317441, Achn157331, Achn385591 |
| Phenylpropanoid biosynthesis | ath00940 | 0.0938 | 13 | Achn353661, Achn166351, Achn256241, Achn153791, Achn213291, Achn207361, Achn022871, Achn270471, Achn166341, Achn132211, Achn058881, Achn356831, Achn157331 |
| Brassinosteroid biosynthesis | ath00905 | 0.0938 | 3 | Achn315541, Achn338411, Achn143751 |
| Biosynthesis of secondary metabolites | ath01110 | 0.0938 | 51 | Achn166351, Achn315541, Achn011201, Achn270471, Achn132211, Achn364251, Achn341521, Achn155541, Achn338411, Achn157331, Achn264541, Achn385591, Achn011691, Achn242531, Achn085041, Achn334011, Achn372361, Achn213291, Achn141711, Achn036391, Achn255821, Achn045991, Achn387711, Achn341891, Achn153791, Achn310371, Achn207361, Achn159771, Achn355121, Achn323101, Achn227791, Achn256241, Achn018501, Achn166341, Achn137801, Achn356831, Achn030701, Achn353661, Achn058881, Achn041311, Achn022871, Achn206791, Achn238811, Achn322041, Achn188121, Achn093931, Achn287101, Achn317441, Achn036401, Achn373661, Achn147791 |
| Linoleic acid metabolism | ath00591 | 0.0972 | 3 | Achn123601, Achn198621, Achn115201 |
| alpha-Linolenic acid metabolism | ath00592 | 0.1049 | 5 | Achn323891, Achn123601, Achn198621, Achn061351, Achn115201 |
| Carbon fixation in photosynthetic organisms | ath00710 | 0.1325 | 7 | Achn133361, Achn137801, Achn100271, Achn039921, Achn287101, Achn373661, Achn147791 |
| Plant-pathogen interaction | ath04626 | 0.1325 | 12 | Achn327821, Achn089421, Achn314301, Achn166581, Achn237651, Achn132821, Achn235441, Achn240741, Achn310371, Achn278571, Achn089411, Achn213151 |
| Ether lipid metabolism | ath00565 | 0.1325 | 4 | Achn150011, Achn343031, Achn002501, Achn150021 |
| Taurine and hypotaurine metabolism | ath00430 | 0.1325 | 3 | Achn323101, Achn244701, Achn058421 |
| Carotenoid biosynthesis | ath00906 | 0.1602 | 4 | Achn260601, Achn098281, Achn011691, Achn188121 |
| Cysteine and methionine metabolism | ath00270 | 0.1722 | 8 | Achn142561, Achn227791, Achn155541, Achn364251, Achn341521, Achn370591, Achn287101, Achn385591 |
| Alanine, aspartate and glutamate metabolism | ath00250 | 0.1722 | 5 | Achn045991, Achn323101, Achn255821, Achn011201, Achn287101 |
| Metabolic pathways | ath01100 | 0.1722 | 80 | Achn092641, Achn166351, Achn142561, Achn150011, Achn011201, Achn338411, Achn002501, Achn315541, Achn132211, Achn364251, Achn341521, Achn174841, Achn141771, Achn126731, Achn370591, Achn264541, Achn061351, Achn036741, Achn302701, Achn011691, Achn156701, Achn242531, Achn085041, Achn115201, Achn343031, Achn372361, Achn213291, Achn277931, Achn036391, Achn123601, Achn255821, Achn330871, Achn385591, Achn045991, Achn387711, Achn155541, Achn028861, Achn153791, Achn270471, Achn207361, Achn141711, Achn067151, Achn323101, Achn198621, Achn227791, Achn133361, Achn041311, Achn256241, Achn244701, Achn018501, Achn166341, Achn125151, Achn137801, Achn118371, Achn039921, Achn356831, Achn150021, Achn334011, Achn294311, Achn353661, Achn058881, Achn336411, Achn204401, Achn377591, Achn333301, Achn022871, Achn157331, Achn238811, Achn322041, Achn188121, Achn058421, Achn093931, Achn100271, Achn287101, Achn317441, Achn269061, Achn323621, Achn036401, Achn373661, Achn147791 |
| Flavonoid biosynthesis | ath00941 | 0.2209 | 3 | Achn022871, Achn270471, Achn085041 |
| Isoquinoline alkaloid biosynthesis | ath00950 | 0.2532 | 3 | Achn317441, Achn385591, Achn287101 |
| Thiamine metabolism | ath00730 | 0.2778 | 2 | Achn242531, Achn333301 |
| Nitrogen metabolism | ath00910 | 0.2778 | 4 | Achn045991, Achn049401, Achn255821, Achn011201 |
| Starch and sucrose metabolism | ath00500 | 0.2907 | 11 | Achn156701, Achn141771, Achn377591, Achn256241, Achn238811, Achn125151, Achn058881, Achn118371, Achn269061, Achn092641, Achn372361 |
| Ubiquinone and other terpenoid-quinone biosynthesis | ath00130 | 0.4094 | 3 | Achn022871, Achn385591, Achn213291 |
| Tropane, piperidine and pyridine alkaloid biosynthesis | ath00960 | 0.4573 | 3 | Achn317441, Achn385591, Achn287101 |
| Diterpenoid biosynthesis | ath00904 | 0.4573 | 2 | Achn209941, Achn140081 |
| Carbon metabolism | ath01200 | 0.4573 | 12 | Achn142561, Achn133361, Achn100271, Achn174841, Achn137801, Achn141711, Achn039921, Achn370591, Achn287101, Achn147791, Achn373661, Achn028861 |
| Pentose and glucuronate interconversions | ath00040 | 0.4573 | 5 | Achn137801, Achn118371, Achn070291, Achn315151, Achn039701 |
| Plant hormone signal transduction | ath04075 | 0.4573 | 13 | Achn160581, Achn359661, Achn283211, Achn240451, Achn328781, Achn049931, Achn100611, Achn240741, Achn167651, Achn194401, Achn357721, Achn164551, Achn067861 |
| Tyrosine metabolism | ath00350 | 0.4573 | 3 | Achn317441, Achn385591, Achn287101 |
| Glycerophospholipid metabolism | ath00564 | 0.4573 | 5 | Achn150011, Achn343031, Achn302701, Achn002501, Achn150021 |
| Pyruvate metabolism | ath00620 | 0.4573 | 5 | Achn100271, Achn039921, Achn174841, Achn133361, Achn147791 |
| Arginine and proline metabolism | ath00330 | 0.6296 | 4 | Achn067151, Achn294311, Achn323621, Achn287101 |
| Glycerolipid metabolism | ath00561 | 0.6296 | 3 | Achn036741, Achn302701, Achn002501 |
| Riboflavin metabolism | ath00740 | 0.6296 | 1 | Achn204401 |
| Biosynthesis of amino acids | ath01230 | 0.6296 | 11 | Achn045991, Achn142561, Achn011201, Achn141711, Achn255821, Achn137801, Achn387711, Achn370591, Achn287101, Achn385591, Achn373661 |
| Pentose phosphate pathway | ath00030 | 0.6296 | 3 | Achn137801, Achn141711, Achn373661 |
| Fatty acid elongation | ath00062 | 0.6296 | 2 | Achn159771, Achn310371 |
| Phenylalanine, tyrosine and tryptophan biosynthesis | ath00400 | 0.6608 | 3 | Achn387711, Achn385591, Achn287101 |
| Vitamin B6 metabolism | ath00750 | 0.7028 | 1 | Achn330871 |
| Stilbenoid, diarylheptanoid and gingerol biosynthesis | ath00945 | 0.7028 | 3 | Achn022871, Achn018501, Achn270471 |
| Nicotinate and nicotinamide metabolism | ath00760 | 0.7028 | 1 | Achn092431 |
| Inositol phosphate metabolism | ath00562 | 0.7028 | 3 | Achn150021, Achn150011, Achn093931 |
| beta-Alanine metabolism | ath00410 | 0.7028 | 2 | Achn317441, Achn323101 |
| Sulfur metabolism | ath00920 | 0.7028 | 2 | Achn142561, Achn370591 |
| Ascorbate and aldarate metabolism | ath00053 | 0.7028 | 2 | Achn277931, Achn334011 |
| Amino sugar and nucleotide sugar metabolism | ath00520 | 0.7028 | 5 | Achn206791, Achn377591, Achn372361, Achn341891, Achn030701 |
| Butanoate metabolism | ath00650 | 0.7288 | 1 | Achn323101 |
| Porphyrin and chlorophyll metabolism | ath00860 | 0.7288 | 2 | Achn041311, Achn322041 |
| Other glycan degradation | ath00511 | 0.7288 | 1 | Achn018971 |
| Glucosinolate biosynthesis | ath00966 | 0.7397 | 1 | Achn355121 |
| Zeatin biosynthesis | ath00908 | 0.8022 | 1 | Achn215031 |
| Cutin, suberine and wax biosynthesis | ath00073 | 0.8022 | 1 | Achn336411 |
| Galactose metabolism | ath00052 | 0.8114 | 2 | Achn092641, Achn389791 |
| Terpenoid backbone biosynthesis | ath00900 | 0.8342 | 2 | Achn264541, Achn242531 |
| Cyanoamino acid metabolism | ath00460 | 0.8424 | 2 | Achn058881, Achn256241 |
| Glyoxylate and dicarboxylate metabolism | ath00630 | 0.8605 | 2 | Achn028861, Achn174841 |
| Circadian rhythm - plant | ath04712 | 0.9333 | 1 | Achn345841 |
| Glycolysis / Gluconeogenesis | ath00010 | 0.9454 | 3 | Achn036391, Achn036401, Achn147791 |
| Fatty acid biosynthesis | ath00061 | 0.9454 | 1 | Achn126731 |
| SNARE interactions in vesicular transport | ath04130 | 0.9962 | 1 | Achn059081 |
| Citrate cycle (TCA cycle) | ath00020 | 0.9962 | 1 | Achn147791 |
| Limonene and pinene degradation | ath00903 | 0.9962 | 1 | Achn018501 |
| Glycine, serine and threonine metabolism | ath00260 | 0.9962 | 1 | Achn317441 |
| Fatty acid metabolism | ath01212 | 0.9962 | 1 | Achn126731 |
| 2-Oxocarboxylic acid metabolism | ath01210 | 0.9962 | 1 | Achn287101 |
| Photosynthesis | ath00195 | 0.9962 | 1 | Achn276911 |
| Glutathione metabolism | ath00480 | 0.9962 | 1 | Achn259181 |
| RNA degradation | ath03018 | 0.9962 | 1 | Achn347191 |
| Pyrimidine metabolism | ath00240 | 0.9962 | 1 | Achn092431 |
| Endocytosis | ath04144 | 0.9962 | 1 | Achn343031 |
| Ubiquitin mediated proteolysis | ath04120 | 0.9962 | 1 | Achn345841 |
| Purine metabolism | ath00230 | 0.9962 | 1 | Achn141711 |
| Protein processing in endoplasmic reticulum | ath04141 | 0.9962 | 2 | Achn045661, Achn061931 |
