# Supplementary material for: Integrated analysis of lncRNA and mRNA transcriptomes reveals the potential regulatory role of lncRNA in kiwifruit ripening and softening
Source: Sci Rep. 2021 Jan 18;11:1671. doi: 10.1038/s41598-021-81155-1 (PMC7814023; doi:10.1038/s41598-021-81155-1)
Supplement: Supplementary file 9 — Supplementary Table S7. [file 41598_2021_81155_MOESM9_ESM.doc]

**Table S7. KEGG analysis of DEGs in RT vs CK**

| **KEGG pathway** | **Map ID** | **Corrected *P* value** | **Gene number** | **Gene ID** |
| --- | --- | --- | --- | --- |
| Starch and sucrose metabolism | ath00500 | 0.0158 | 10 | Achn141771, Achn092641, Achn380931, Achn322221, Achn213731, Achn161011, Achn319051, Achn269061, Achn256701, Achn372361 |
| Brassinosteroid biosynthesis | ath00905 | 0.1479 | 2 | Achn379971, Achn143751 |
| Linoleic acid metabolism | ath00591 | 0.1479 | 2 | Achn123601, Achn123621 |
| Circadian rhythm - plant | ath04712 | 0.1853 | 3 | Achn046761, Achn150741, Achn345841 |
| Flavonoid biosynthesis | ath00941 | 0.3676 | 2 | Achn085041, Achn006311 |
| Carotenoid biosynthesis | ath00906 | 0.4839 | 2 | Achn209971, Achn260601 |
| alpha-Linolenic acid metabolism | ath00592 | 0.4839 | 2 | Achn123601, Achn123621 |
| Biosynthesis of secondary metabolites | ath01110 | 0.4839 | 19 | Achn085041, Achn227791, Achn364321, Achn095091, Achn213731, Achn256701, Achn161011, Achn141711, Achn199771, Achn379971, Achn380931, Achn150611, Achn346941, Achn209971, Achn341891, Achn210351, Achn006311, Achn372361, Achn302941 |
| Amino sugar and nucleotide sugar metabolism | ath00520 | 0.4839 | 4 | Achn341891, Achn161011, Achn372361, Achn256701 |
| Pentose and glucuronate interconversions | ath00040 | 0.5035 | 3 | Achn199771, Achn315151, Achn319051 |
| Glutathione metabolism | ath00480 | 0.5278 | 3 | Achn144051, Achn214751, Achn223211 |
| Cysteine and methionine metabolism | ath00270 | 0.5278 | 3 | Achn227791, Achn150611, Achn193321 |
| Glycerolipid metabolism | ath00561 | 0.5278 | 2 | Achn199771, Achn323261 |
| Plant hormone signal transduction | ath04075 | 0.5278 | 6 | Achn359661, Achn131571, Achn269761, Achn227711, Achn117881, Achn067551 |
| Galactose metabolism | ath00052 | 0.5278 | 2 | Achn383841, Achn092641 |
| Glycolysis / Gluconeogenesis | ath00010 | 0.5278 | 3 | Achn210351, Achn199771, Achn346941 |
| Limonene and pinene degradation | ath00903 | 0.5278 | 2 | Achn199771, Achn364321 |
| Stilbenoid, diarylheptanoid and gingerol biosynthesis | ath00945 | 0.5278 | 2 | Achn006311, Achn364321 |
| Other glycan degradation | ath00511 | 0.5278 | 1 | Achn207521 |
| Histidine metabolism | ath00340 | 0.5278 | 1 | Achn199771 |
| Diterpenoid biosynthesis | ath00904 | 0.5278 | 1 | Achn209941 |
| Glycine, serine and threonine metabolism | ath00260 | 0.5480 | 2 | Achn210351, Achn346941 |
| Arginine and proline metabolism | ath00330 | 0.5878 | 2 | Achn199771, Achn193321 |
| Lysine degradation | ath00310 | 0.5948 | 1 | Achn199771 |
| Metabolic pathways | ath01100 | 0.5948 | 28 | Achn322221, Achn269061, Achn210351, Achn085041, Achn123601, Achn161011, Achn123621, Achn141711, Achn379971, Achn150611, Achn346941, Achn209971, Achn380931, Achn141771, Achn227791, Achn095091, Achn374421, Achn323261, Achn319051, Achn199771, Achn193321, Achn092641, Achn364321, Achn213731, Achn038551, Achn006311, Achn372361, Achn302941 |
| Glycerophospholipid metabolism | ath00564 | 0.5948 | 2 | Achn374421, Achn038551 |
| Phagosome | ath04145 | 0.5996 | 2 | Achn285831, Achn185171 |
| Steroid biosynthesis | ath00100 | 0.6593 | 1 | Achn302941 |
| Fatty acid degradation | ath00071 | 0.6672 | 1 | Achn199771 |
| beta-Alanine metabolism | ath00410 | 0.6672 | 1 | Achn199771 |
| Ascorbate and aldarate metabolism | ath00053 | 0.6672 | 1 | Achn199771 |
| Base excision repair | ath03410 | 0.6672 | 1 | Achn152391 |
| Valine, leucine and isoleucine degradation | ath00280 | 0.6672 | 1 | Achn199771 |
| Tryptophan metabolism | ath00380 | 0.6672 | 1 | Achn199771 |
| Pentose phosphate pathway | ath00030 | 0.7345 | 1 | Achn141711 |
| Terpenoid backbone biosynthesis | ath00900 | 0.7440 | 1 | Achn095091 |
| Cyanoamino acid metabolism | ath00460 | 0.7440 | 1 | Achn213731 |
| Ubiquitin mediated proteolysis | ath04120 | 0.7741 | 2 | Achn150741, Achn345841 |
| Phenylpropanoid biosynthesis | ath00940 | 0.7741 | 2 | Achn213731, Achn006311 |
| Carbon metabolism | ath01200 | 0.7741 | 3 | Achn141711, Achn210351, Achn346941 |
| Plant-pathogen interaction | ath04626 | 0.7741 | 2 | Achn162021, Achn314301 |
| Biosynthesis of amino acids | ath01230 | 0.7741 | 3 | Achn141711, Achn210351, Achn346941 |
| Pyruvate metabolism | ath00620 | 0.7741 | 1 | Achn199771 |
| Ribosome biogenesis in eukaryotes | ath03008 | 0.8233 | 1 | Achn116421 |
| RNA degradation | ath03018 | 0.8233 | 1 | Achn301111 |
| Phenylalanine metabolism | ath00360 | 0.8233 | 1 | Achn006311 |
| mRNA surveillance pathway | ath03015 | 0.8233 | 1 | Achn161141 |
| Purine metabolism | ath00230 | 0.8909 | 1 | Achn141711 |
| Oxidative phosphorylation | ath00190 | 0.8909 | 1 | Achn130531 |
