# Supplementary material for: Integrated analysis of lncRNA and mRNA transcriptomes reveals the potential regulatory role of lncRNA in kiwifruit ripening and softening
Source: Sci Rep. 2021 Jan 18;11:1671. doi: 10.1038/s41598-021-81155-1 (PMC7814023; doi:10.1038/s41598-021-81155-1)
Supplement: Supplementary file 10 — Supplementary Table S8. [file 41598_2021_81155_MOESM10_ESM.doc]

**Table S8. DEGs encoded key enzymes involved in fruit ripening and softening**

| **Group** | **Term** | **Transcript ID** | **Length/bp** | **FPKM (ABA)** | **FPKM (CK)** | **Log_2_FPKM (ABA vs CK)** | **Description** |
| --- | --- | --- | --- | --- | --- | --- | --- |
| **ABA vs CK** | ethylene biosynthesis | Achn364251 | 1356 | 310.88 | 0.485217 | 9.32351 | 1-aminocyclopropane-1-carboxylate synthase |
|  |  | Achn155541 | 963 | 604.964 | 0.964332 | 9.2931 | 1-aminocyclopropane-1-carboxylate oxidase 1 |
|  |  | Achn227791 | 777 | 41.6135 | 0.197882 | 7.71627 | 1-aminocyclopropane-1-carboxylate oxidase |
|  |  | Achn341521 | 936 | 8551.23 | 271.263 | 4.97837 | 1-aminocyclopropane-1-carboxylate oxidase |
|  | ethylene signaling pathway | Achn189151 | 876 | 395.815 | 1.00817 | 8.61695 | ethylene-responsive transcription factor |
|  |  | Achn318101 | 837 | 122.474 | 0.357291 | 8.42116 | ethylene response factor 4 |
|  |  | Achn187281 | 510 | 298.454 | 1.51974 | 7.61754 | ethylene response factor 16 |
|  |  | Achn064481 | 888 | 236.185 | 2.55766 | 6.52895 | ethylene response factor 4 |
|  |  | Achn344761 | 420 | 82.4052 | 1.1013 | 6.22546 | ethylene response factor |
|  |  | Achn362941 | 702 | 534.458 | 8.11739 | 6.04092 | ethylene response factor 7 |
|  |  | Achn359661 | 651 | 41.0201 | 0.767108 | 5.74076 | ethylene response factor 10 |
|  |  | Achn290601 | 1437 | 10.0465 | 0.226464 | 5.47126 | ethylene-responsive transcription factor |
|  |  | Achn062471 | 789 | 100.285 | 3.38992 | 4.88671 | ethylene response factor 12 |
|  |  | Achn054961 | 1029 | 64.3225 | 3.13394 | 4.35927 | ethylene response factor 3 |
|  |  | Achn024671 | 876 | 334.637 | 27.3886 | 3.61095 | ethylene response factor 4 |
|  |  | Achn311001 | 882 | 114.22 | 6.74348 | 4.08218 | ethylene-responsive transcription factor |
|  |  | Achn336111 | 1737 | 31.2934 | 0.545055 | 5.84331 | REVERSION-TO-ETHYLENE SENSITIVITY1 |
|  |  | Achn170441 | 1083 | 40.7507 | 0.447071 | 6.51018 | AP2/ERF and B3 domain-containing transcription factor RAV1 |
|  |  | Achn164551 | 1935 | 190.458 | 16.3926 | 3.53836 | EIN3-binding F-box protein 1 |
|  |  | Achn194401 | 2025 | 202.992 | 10.689 | 4.24723 | ethylene receptor 2 |
|  |  | Achn067861 | 2298 | 427.973 | 24.9894 | 4.09813 | ethylene receptor 2 |
|  | starch and sucrose degradation | Achn156701 | 2517 | 99.6523 | 3.98385 | 4.64467 | alpha,alpha-trehalose-phosphate synthase 7 |
|  |  | Achn372361 | 1302 | 3.46827 | 255.988 | -6.20572 | ADP-glucose pyrophosphorylase large subunit 4 |
|  |  | Achn238811 | 2691 | 26.5906 | 386.33 | -3.86084 | alpha-glucan phosphorylase, H isozyme |
|  |  | Achn141771 | 1305 | 270.293 | 10.1604 | 4.7335 | beta-amylase |
|  |  | Achn269061 | 1410 | 1051.22 | 14.4983 | 6.18004 | beta-amylase |
|  |  | Achn092641 | 3063 | 25.9977 | 0.232685 | 6.80387 | invertase 5 |
|  |  | Achn256241 | 1773 | 0.901768 | 22.3617 | -4.63213 | lysosomal beta glucosidase-like |
|  |  | Achn058881 | 1914 | 64.6685 | 2.6358 | 4.61675 | lysosomal beta glucosidase-like |
|  |  | Achn118371 | 1545 | 12.1977 | 0.0832032 | 7.19575 | putative style polygalacturonase |
|  |  | Achn125151 | 1836 | 26.5967 | 2.01256 | 3.72414 | trehalose-6-phosphate synthase |
|  |  | Achn377591 | 981 | 87.3664 | 6.73555 | 3.69721 | UDP-glucuronate 4-epimerase 1 |
|  | cell wall degradation | Achn005701 | 1524 | 302.464 | 0.169086 | 10.8048 | endoglucanase-like |
|  |  | Achn281771 | 1182 | 19.8693 | 0.28648 | 6.11596 | endoglucanase 1-like isoform X2 |
|  |  | Achn132361 | 1452 | 14.3981 | 0.31319 | 5.52269 | endoglucanase-like |
|  |  | Achn294591 | 1767 | 0.301768 | 6.48511 | -4.42562 | endoglucanase 25 |
|  |  | Achn319971 | 1575 | 0.191707 | 13.0992 | -6.09443 | endoglucanase 11-like |
|  |  | Achn102911 | 960 | 321.843 | 6.92664 | 5.53806 | class I beta-1,3-glucanase precursor |
|  |  | Achn103301 | 942 | 13.0019 | 0.152673 | 6.41214 | beta-1,3-glucanase |
|  |  | Achn123061 | 720 | 64.3703 | 0.661292 | 6.60496 | beta-galactosidase 16 |
|  |  | Achn123051 | 1161 | 17.7114 | 0.585745 | 4.91826 | Beta-galactosidase 16 |
|  |  | Achn294421 | 1830 | 63.1644 | 2.84189 | 4.47419 | beta-galactosidase 3 |
|  |  | Achn039701 | 1173 | 405.866 | 2.14037 | 7.567 | probable pectate lyase 18 |
|  |  | Achn315151 | 2025 | 27.4768 | 0.611199 | 5.49043 | probable pectate lyase 18 |
|  |  | Achn367751 | 885 | 165.733 | 1.49179 | 6.79567 | xyloglucan endotransglucosylase/hydrolase 7 |
|  |  | Achn349841 | 309 | 113.915 | 1.83512 | 5.95594 | xyloglucan endotransglucosylase/hydrolase 5 |
|  |  | Achn349851 | 447 | 194.953 | 3.6394 | 5.74328 | xyloglucan endotransglucosylase/hydrolase 5 |
|  |  | Achn009121 | 837 | 43.4372 | 1.16119 | 5.22525 | xyloglucan endotransglucosylase/hydrolase 7 |
|  |  | Achn376911 | 852 | 110.449 | 0.610423 | 7.49935 | xyloglucan endotransglucosylase/hydrolase 7 |
|  |  | Achn080501 | 645 | 26.5334 | 0.129655 | 7.67698 | polygalacturonase A |
|  |  | Achn269821 | 711 | 23.3796 | 0.336704 | 6.11762 | Expansin-related protein 1 precursor |
|  |  | Achn059761 | 2316 | 30.326 | 0.263003 | 6.84933 | putative beta-D-xylosidase |
|  |  | Achn287321 | 1524 | 11.2806 | 0.549529 | 4.35951 | LOW QUALITY PROTEIN: probable glucan endo-1,3-beta-glucosidase A6 |
|  |  | Achn165841 | 2556 | 0.243337 | 21.097 | -6.43794 | cellulose synthase A |
|  |  | Achn107271 | 987 | 4.17262 | 0.0718589 | 5.85964 | Secondary cell wall-related glycosyltransferase family |
|  |  | Achn207361 | 1566 | 72.6859 | 1.72001 | 5.40119 | ferulate 5-hydroxylase |
|  | membrane lipid peroxidation | Achn198621 | 3054 | 21.7618 | 0.369587 | 5.87974 | lipoxygenase |
|  |  | Achn115201 | 2721 | 47.9335 | 1.76334 | 4.76466 | linoleate 13S-lipoxygenase 3-1, chloroplastic |
|  |  | Achn123601 | 2445 | 0.859646 | 12.1153 | -3.81694 | linoleate 13S-lipoxygenase 2-1, chloroplastic-like |
|  | brassinosteroid biosynthesis | Achn315541 | 1215 | 199.279 | 19.9426 | 3.32086 | brassinosteroid-6-oxidase |
|  |  | Achn338411 | 1152 | 216.477 | 8.69357 | 4.63812 | unnamed protein product |
|  |  | Achn143751 | 1362 | 525.827 | 40.7372 | 3.69017 | cytochrome P450 CYP749A22-like |
|  | phenylalanine metabolism | Achn353661 | 930 | 5780.57 | 187.079 | 4.94949 | peroxidase 55-like |
|  |  | Achn166351 | 2043 | 125.041 | 10.8858 | 3.52188 | phenylalanine ammonia-lyase |
|  |  | Achn153791 | 1704 | 39.5858 | 0.0742899 | 9.0576 | phenylalanine ammonia-lyase |
|  |  | Achn213291 | 1632 | 0.294198 | 10.8137 | -5.19993 | 4-coumarate:CoA ligase |
|  |  | Achn022871 | 1350 | 6.91393 | 0.0975586 | 6.14709 | trans-cinnamate 4-hydroxylase |
|  |  | Achn166341 | 1689 | 20.4567 | 1.01315 | 4.33565 | unnamed protein product |
|  |  | Achn132211 | 957 | 27.9316 | 0.224349 | 6.96001 | peroxidase 64 |
|  |  | Achn356831 | 984 | 219.359 | 1.15426 | 7.57019 | peroxidase 27-like |
|  |  | Achn287101 | 1665 | 35.386 | 0.877388 | 5.33382 | hypothetical protein |
|  |  | Achn317441 | 2106 | 9.44209 | 116.504 | -3.62513 | Amine oxidase |
|  |  | Achn157331 | 987 | 1.16286 | 28.7436 | -4.62749 | peroxidase 17 |
|  |  | Achn385591 | 1206 | 5.77079 | 84.1639 | -3.86636 | hypothetical protein |
|  | transcription factor | Achn137671 | 993 | 731.81 | 65.4546 | 3.4829 | MYB transcription factor 1 |
|  |  | Achn228371 | 831 | 26.8611 | 0.721596 | 5.21818 | MYB12 family protein |
|  |  | Achn324811 | 825 | 54.5322 | 1.00203 | 5.76611 | R2R3 transcription factor MYB108-like protein 1 |
|  |  | Achn172271 | 873 | 11.6965 | 0.337599 | 5.11463 | transcription factor MYB59 |
|  |  | Achn163771 | 948 | 787.656 | 46.6357 | 4.07806 | NAC protein 2 |
|  |  | Achn134171 | 960 | 1151.87 | 56.3069 | 4.35452 | NAC protein 3 |
|  |  | Achn179541 | 738 | 1685.79 | 126.601 | 3.73507 | NAC protein 4 |
|  |  | Achn037901 | 1302 | 22.7364 | 0.101886 | 7.80191 | probable WRKY transcription factor 14 |
|  |  | Achn165131 | 1311 | 25.4597 | 1.31359 | 4.27663 | probable WRKY transcription factor 14 |
|  |  | Achn150821 | 1377 | 54.2264 | 3.47781 | 3.96274 | probable WRKY transcription factor 31 |
|  |  | Achn026311 | 1731 | 72.3153 | 5.43571 | 3.73376 | probable WRKY transcription factor 31 |
|  |  | Achn314301 | 2067 | 74.6797 | 7.2823 | 3.35825 | probable WRKY transcription factor 33 |
|  |  | Achn160161 | 780 | 10.6766 | 0.098411 | 6.76142 | probable WRKY transcription factor 40 |
|  |  | Achn278571 | 876 | 30.188 | 1.34422 | 4.48913 | WRKY transcription factor 22 |
|  |  | Achn132821 | 834 | 9.96858 | 0.0897589 | 6.79519 | WRKY transcription factor 22-like isoform X2 |
|  |  | Achn294851 | 900 | 46.97 | 2.67464 | 4.13432 | GATA transcription factor 5-like |
|  |  | Achn166151 | 1167 | 33.1532 | 0.756645 | 5.45339 | GATA transcription factor 8-like |
|  |  | Achn169421 | 849 | 33.3642 | 1.05143 | 4.98787 | NAP-like transcription factor |
|  |  | Achn182861 | 819 | 311.438 | 21.3454 | 3.86695 | NAP-like transcription factor |
|  |  | Achn251771 | 1146 | 212.037 | 1.07142 | 7.62865 | probable transcription factor GLK2 |
|  |  | Achn084161 | 822 | 4.12185 | 101.343 | -4.61981 | probable transcription factor KAN2 isoform X2 |
|  |  | Achn232701 | 1263 | 17.6005 | 0.317084 | 5.79461 | transcription factor bHLH63 |
|  |  | Achn135561 | 447 | 241.859 | 12.1313 | 4.31736 | bZIP transcription factor family protein |
| **RT vs CK** | ethylene biosynthesis | Achn227791 | 777 | 18.8546 | 0.197882 | 6.57413 | 1-aminocyclopropane-1-carboxylate oxidase |
|  |  | Achn246401 | 1191 | 598.765 | 55.4028 | 3.43396 | 1-aminocyclopropane-1-carboxylate oxidase homolog 3-like |
|  |  | Achn150611 | 870 | 426.304 | 4.40904 | 6.59527 | ACC oxidase 4 |
|  |  | Achn093111 | 1629 | 32.4646 | 1.44751 | 4.48722 | ACC synthase 10 isoform 1 |
|  | ethylene signaling pathway | Achn359661 | 651 | 87.6434 | 0.767108 | 6.83607 | ethylene response factor 10 |
|  |  | Achn054961 | 1029 | 158.65 | 3.13394 | 5.66173 | ethylene response factor 3 |
|  |  | Achn362941 | 702 | 194.236 | 8.11739 | 4.58065 | ethylene response factor 7 |
|  | starch and sucrose degradation | Achn141771 | 1305 | 155.664 | 10.1604 | 3.93741 | beta-amylase |
|  |  | Achn092641 | 3063 | 8.81392 | 0.232685 | 5.24334 | invertase 5 |
|  |  | Achn380931 | 3702 | 27.2031 | 1.50406 | 4.17684 | alpha-glucan phosphorylase family protein |
|  |  | Achn322221 | 1275 | 551.375 | 0.940434 | 9.19549 | beta-amylase |
|  |  | Achn213731 | 1563 | 17.2575 | 0.615662 | 4.80894 | beta-glucosidase 44-like |
|  |  | Achn161011 | 1518 | 199.687 | 16.8169 | 3.56976 | AGP-glucose pyrophosphorylase |
|  |  | Achn319051 | 1587 | 93.1912 | 2.5075 | 5.21587 | probable pectinesterase/pectinesterase inhibitor 59 |
|  |  | Achn269061 | 1410 | 170.373 | 14.4983 | 3.55475 | beta-amylase |
|  |  | Achn256701 | 1008 | 82.538 | 4.9663 | 4.05482 | Galacturonosyltransferase 13 isoform 1 |
|  |  | Achn372361 | 1302 | 19.2462 | 255.988 | -3.73343 | ADP-glucose pyrophosphorylase large subunit 4 |
|  | cell wall degradation | Achn294421 | 1830 | 40.2777 | 2.84189 | 3.82506 | beta-galactosidase 3 |
|  |  | Achn315151 | 2025 | 72.4499 | 0.611199 | 6.8892 | probable pectate lyase 18 |
|  |  | Achn023381 | 837 | 41.9869 | 0.267968 | 7.29173 | xyloglucan endotransglucosylase/hydrolase 10 |
|  |  | Achn352601 | 1773 | 381.294 | 14.9788 | 4.66991 | xyloglucan galactosyltransferase KATAMARI1 |
|  |  | Achn177571 | 1086 | 120.191 | 2.67313 | 5.49065 | Glucan endo-1,3-beta-glucosidase precursor, putative |
|  |  | Achn387971 | 2472 | 12.7141 | 0.0244786 | 9.02069 | probable beta-D-xylosidase 7 |
|  |  | Achn042871 | 1302 | 175.615 | 10.3415 | 4.08591 | mannan endo-1,4-beta-mannosidase 2-like |
|  | membrane lipid peroxidation | Achn123601 | 2445 | 0.374869 | 12.1153 | -5.01429 | linoleate 13S-lipoxygenase 2-1, chloroplastic-like |
|  |  | Achn123621 | 2013 | 6.34788 | 0.0922328 | 6.10485 | lipoxygenase 6 |
|  | brassinosteroid biosynthesis | Achn379971 | 1353 | 15.6697 | 0.827053 | 4.24385 | cytochrome P450 90B1 |
|  |  | Achn143751 | 1362 | 678.357 | 40.7372 | 4.05762 | cytochrome P450 CYP749A22-like |
|  | phenylalanine metabolism | Achn006311 | 1509 | 137.839 | 11.5461 | 3.57751 | caffeoyl-CoA-O-methyltransferase |
|  | transcription factor | Achn289291 | 993 | 1464.21 | 58.0392 | 4.65696 | NAC domain class transcription factor isoform 1 |
|  |  | Achn111051 | 969 | 1389.85 | 110.378 | 3.6544 | NAC domain containing protein 25 |
|  |  | Achn169421 | 849 | 518.298 | 1.05143 | 8.94529 | NAP-like transcription factor |
|  |  | Achn311801 | 705 | 172.198 | 1.13623 | 7.24368 | NAP-like transcription factor |
|  |  | Achn182861 | 819 | 236.648 | 21.3454 | 3.47075 | NAP-like transcription factor |
|  |  | Achn026311 | 1731 | 104.168 | 5.43571 | 4.26029 | probable WRKY transcription factor 31 |
|  |  | Achn314301 | 2067 | 113.823 | 7.2823 | 3.96626 | probable WRKY transcription factor 33 |
|  |  | Achn227711 | 1236 | 225.164 | 5.4794 | 5.36081 | bZIP transcription factor bZIP8 |
|  |  | Achn131571 | 1296 | 7.344 | 0.153681 | 5.57856 | bZIP transcription factor bZIP8 |
|  |  | Achn298101 | 693 | 18.9635 | 0.699023 | 4.76174 | transcription factor MYB39 isoform X2 |
|  |  | Achn116381 | 690 | 0.748742 | 20.401 | -4.76803 | nuclear transcription factor Y subunit C-1 |
