# Supplementary material for: Integrated analysis of lncRNA and mRNA transcriptomes reveals the potential regulatory role of lncRNA in kiwifruit ripening and softening
Source: Sci Rep. 2021 Jan 18;11:1671. doi: 10.1038/s41598-021-81155-1 (PMC7814023; doi:10.1038/s41598-021-81155-1)
Supplement: Supplementary file 11 — Supplementary Table S9. [file 41598_2021_81155_MOESM11_ESM.doc]

**Table S9. KEGG analysis of the target genes of DELs in ABA vs CK**

| **KEGG pathway** | **Map ID** | **Corrected *P* value** | **Gene number** | **Gene ID** |
| --- | --- | --- | --- | --- |
| Starch and sucrose metabolism | ath00500 | 0.0281 | 18 | Achn256221, Achn173741, Achn092741, Achn024141, Achn341491, Achn240251, Achn096461, Achn322221, Achn143581, Achn270701, Achn140461, Achn345721, Achn140471, Achn240191, Achn140481, Achn145021, Achn240181, Achn256211 |
| RNA degradation | ath03018 | 0.9723 | 8 | Achn056951, Achn056931, Achn347141, Achn347191, Achn049951, Achn056941, Achn049961, Achn231841 |
| Isoquinoline alkaloid biosynthesis | ath00950 | 0.9723 | 3 | Achn186891, Achn093251, Achn093241 |
| Pentose and glucuronate interconversions | ath00040 | 0.9723 | 6 | Achn099161, Achn183701, Achn140471, Achn240191, Achn140481, Achn240181 |
| Ribosome biogenesis in eukaryotes | ath03008 | 0.9723 | 7 | Achn056931, Achn295361, Achn291491, Achn056951, Achn056941, Achn270721, Achn198301 |
| Stilbenoid, diarylheptanoid and gingerol biosynthesis | ath00945 | 0.9723 | 5 | Achn018501, Achn328801, Achn328861, Achn328851, Achn270471 |
| Metabolic pathways | ath01100 | 0.9723 | 78 | Achn018791, Achn132211, Achn360761, Achn183231, Achn231771, Achn341541, Achn322221, Achn050721, Achn192811, Achn107221, Achn328861, Achn132181, Achn252301, Achn077231, Achn018761, Achn270701, Achn347081, Achn018821, Achn140471, Achn140461, Achn099481, Achn195531, Achn240181, Achn071311, Achn092741, Achn103581, Achn036391, Achn119711, Achn264191, Achn170781, Achn328851, Achn231831, Achn096471, Achn286141, Achn345721, Achn347121, Achn270471, Achn240251, Achn140481, Achn186891, Achn252241, Achn291641, Achn270481, Achn213261, Achn024141, Achn096371, Achn018501, Achn094631, Achn256221, Achn143581, Achn231781, Achn256141, Achn240591, Achn240191, Achn107281, Achn145021, Achn093241, Achn231751, Achn049951, Achn173741, Achn341521, Achn341491, Achn130621, Achn256211, Achn240511, Achn186901, Achn096461, Achn236251, Achn093251, Achn236301, Achn390101, Achn099111, Achn328801, Achn131071, Achn036401, Achn173761, Achn195471, Achn041381 |
| Phagosome | ath04145 | 0.9723 | 6 | Achn094611, Achn096441, Achn344491, Achn096661, Achn095821, Achn366781 |
| SNARE interactions in vesicular transport | ath04130 | 0.9723 | 4 | Achn265121, Achn096661, Achn096441, Achn347111 |
| Plant-pathogen interaction | ath04626 | 0.9723 | 9 | Achn193431, Achn130621, Achn056901, Achn071331, Achn240271, Achn095791, Achn213151, Achn231801, Achn208701 |
| Diterpenoid biosynthesis | ath00904 | 0.9723 | 2 | Achn228231, Achn103531 |
| Tropane, piperidine and pyridine alkaloid biosynthesis | ath00960 | 0.9723 | 3 | Achn186891, Achn093251, Achn093241 |
| One carbon pool by folate | ath00670 | 0.9723 | 2 | Achn270481, Achn252301 |
| Tyrosine metabolism | ath00350 | 0.9723 | 3 | Achn186891, Achn093251, Achn093241 |
| Flavonoid biosynthesis | ath00941 | 0.9723 | 2 | Achn270471, Achn173761 |
| Limonene and pinene degradation | ath00903 | 0.9723 | 4 | Achn018501, Achn328801, Achn328861, Achn328851 |
| Inositol phosphate metabolism | ath00562 | 0.9723 | 4 | Achn264191, Achn096471, Achn119711, Achn213261 |
| N-Glycan biosynthesis | ath00510 | 0.9723 | 3 | Achn390101, Achn096371, Achn195471 |
| Monoterpenoid biosynthesis | ath00902 | 0.9723 | 1 | Achn264261 |
| Pyrimidine metabolism | ath00240 | 0.9723 | 6 | Achn071311, Achn107221, Achn107281, Achn018821, Achn131071, Achn186901 |
| Endocytosis | ath04144 | 0.9723 | 6 | Achn176801, Achn102361, Achn341531, Achn231761, Achn231821, Achn198291 |
| Carotenoid biosynthesis | ath00906 | 0.9723 | 2 | Achn208641, Achn347121 |
| Arginine and proline metabolism | ath00330 | 0.9723 | 4 | Achn018761, Achn041381, Achn132181, Achn186891 |
| Cysteine and methionine metabolism | ath00270 | 0.9723 | 5 | Achn341521, Achn231831, Achn099481, Achn256141, Achn186891 |
| Phenylpropanoid biosynthesis | ath00940 | 0.9723 | 7 | Achn018791, Achn132211, Achn270471, Achn256221, Achn256211, Achn099111, Achn195531 |
| Cyanoamino acid metabolism | ath00460 | 0.9723 | 3 | Achn256221, Achn347081, Achn256211 |
| Glycolysis / Gluconeogenesis | ath00010 | 0.9723 | 5 | Achn049951, Achn036391, Achn264191, Achn036401, Achn103581 |
| mRNA surveillance pathway | ath03015 | 0.9723 | 5 | Achn322181, Achn198321, Achn341971, Achn049961, Achn099441 |
| Phenylalanine metabolism | ath00360 | 0.9723 | 5 | Achn093251, Achn132211, Achn186891, Achn195531, Achn093241 |
| Biotin metabolism | ath00780 | 0.9723 | 1 | Achn240511 |
| beta-Alanine metabolism | ath00410 | 0.9723 | 2 | Achn093251, Achn093241 |
| Lysine biosynthesis | ath00300 | 0.9723 | 1 | Achn252241 |
| Ubiquitin mediated proteolysis | ath04120 | 0.9723 | 6 | Achn295351, Achn103541, Achn295341, Achn295321, Achn009921, Achn192831 |
| Glycine, serine and threonine metabolism | ath00260 | 0.9723 | 3 | Achn093251, Achn103581, Achn093241 |
| Base excision repair | ath03410 | 0.9723 | 2 | Achn295181, Achn265231 |
| Arachidonic acid metabolism | ath00590 | 0.9723 | 1 | Achn094631 |
| Selenocompound metabolism | ath00450 | 0.9723 | 1 | Achn256141 |
| Other glycan degradation | ath00511 | 0.9723 | 1 | Achn231781 |
| Histidine metabolism | ath00340 | 0.9723 | 1 | Achn077231 |
| RNA polymerase | ath03020 | 0.9723 | 2 | Achn131071, Achn018821 |
| Purine metabolism | ath00230 | 0.9723 | 6 | Achn183231, Achn252301, Achn107281, Achn018821, Achn131071, Achn186901 |
| Alanine, aspartate and glutamate metabolism | ath00250 | 0.9723 | 2 | Achn041381, Achn186891 |
| DNA replication | ath03030 | 0.9723 | 2 | Achn345461, Achn186901 |
| Folate biosynthesis | ath00790 | 0.9723 | 1 | Achn286141 |
| Propanoate metabolism | ath00640 | 0.9723 | 1 | Achn360761 |
| Fructose and mannose metabolism | ath00051 | 0.9723 | 2 | Achn049951, Achn264191 |
| RNA transport | ath03013 | 0.9723 | 6 | Achn322181, Achn056971, Achn195821, Achn291491, Achn252511, Achn049961 |
| Protein export | ath03060 | 0.9723 | 2 | Achn195541, Achn344491 |
| ABC transporters | ath02010 | 0.9723 | 1 | Achn096641 |
| Galactose metabolism | ath00052 | 0.9723 | 2 | Achn049951, Achn173741 |
| Lysine degradation | ath00310 | 0.9723 | 1 | Achn240591 |
| Homologous recombination | ath03440 | 0.9723 | 2 | Achn240231, Achn345461 |
| Phenylalanine, tyrosine and tryptophan biosynthesis | ath00400 | 0.9723 | 2 | Achn236251, Achn186891 |
| Sphingolipid metabolism | ath00600 | 0.9723 | 1 | Achn231781 |
| Ether lipid metabolism | ath00565 | 0.9723 | 1 | Achn119711 |
| Plant hormone signal transduction | ath04075 | 0.9723 | 9 | Achn231151, Achn328781, Achn049931, Achn195831, Achn345691, Achn240531, Achn049901, Achn328881, Achn099501 |
| Citrate cycle (TCA cycle) | ath00020 | 0.9723 | 2 | Achn360761, Achn103581 |
| Phosphatidylinositol signaling system | ath04070 | 0.9723 | 2 | Achn096471, Achn213261 |
| alpha-Linolenic acid metabolism | ath00592 | 0.9723 | 1 | Achn231751 |
| Biosynthesis of amino acids | ath01230 | 0.9723 | 8 | Achn049951, Achn256141, Achn252241, Achn264191, Achn077231, Achn236251, Achn041381, Achn186891 |
| Carbon fixation in photosynthetic organisms | ath00710 | 0.9723 | 2 | Achn170781, Achn264191 |
| Biosynthesis of secondary metabolites | ath01110 | 0.9723 | 33 | Achn018791, Achn132211, Achn360761, Achn183231, Achn270471, Achn252241, Achn252301, Achn049951, Achn270701, Achn041381, Achn195531, Achn347121, Achn103581, Achn036391, Achn093251, Achn264191, Achn328851, Achn236251, Achn093241, Achn018501, Achn256221, Achn256141, Achn240591, Achn264261, Achn186891, Achn077231, Achn341521, Achn328861, Achn099111, Achn328801, Achn036401, Achn173761, Achn256211 |
| Circadian rhythm - plant | ath04712 | 0.9723 | 1 | Achn162721 |
| Mismatch repair | ath03430 | 0.9723 | 1 | Achn345461 |
| Fatty acid biosynthesis | ath00061 | 0.9723 | 1 | Achn240511 |
| Photosynthesis | ath00195 | 0.9723 | 2 | Achn050721, Achn347161 |
| Sulfur metabolism | ath00920 | 0.9723 | 1 | Achn231771 |
| Pyruvate metabolism | ath00620 | 0.9723 | 2 | Achn170781, Achn103581 |
| Valine, leucine and isoleucine degradation | ath00280 | 0.9723 | 1 | Achn103581 |
| Porphyrin and chlorophyll metabolism | ath00860 | 0.9723 | 1 | Achn301861 |
| Spliceosome | ath03040 | 0.9723 | 5 | Achn357041, Achn076861, Achn024231, Achn024191, Achn024211 |
| Oxidative phosphorylation | ath00190 | 0.9723 | 4 | Achn130531, Achn192811, Achn236301, Achn291641 |
| Glycerolipid metabolism | ath00561 | 0.9723 | 1 | Achn130621 |
| Pentose phosphate pathway | ath00030 | 0.9723 | 1 | Achn049951 |
| Basal transcription factors | ath03022 | 0.9723 | 1 | Achn341171 |
| Carbon metabolism | ath01200 | 0.9723 | 6 | Achn270481, Achn170781, Achn360761, Achn103581, Achn264191, Achn049951 |
| Proteasome | ath03050 | 0.9723 | 1 | Achn144961 |
| Protein processing in endoplasmic reticulum | ath04141 | 0.9723 | 5 | Achn096371, Achn383281, Achn344491, Achn195811, Achn095791 |
| Nucleotide excision repair | ath03420 | 0.9723 | 1 | Achn345461 |
| Aminoacyl-tRNA biosynthesis | ath00970 | 0.9723 | 2 | Achn271321, Achn120491 |
| Fatty acid metabolism | ath01212 | 0.9723 | 1 | Achn240511 |
| 2-Oxocarboxylic acid metabolism | ath01210 | 0.9723 | 1 | Achn186891 |
| Amino sugar and nucleotide sugar metabolism | ath00520 | 0.9723 | 2 | Achn270701, Achn092741 |
| Peroxisome | ath04146 | 0.9723 | 1 | Achn341541 |
| Glycerophospholipid metabolism | ath00564 | 0.9723 | 1 | Achn119711 |
| Glutathione metabolism | ath00480 | 0.9763 | 1 | Achn107281 |
| Ribosome | ath03010 | 0.9964 | 5 | Achn102721, Achn301831, Achn345701, Achn295311, Achn018531 |
