# Supplementary material for: Integrated analysis of lncRNA and mRNA transcriptomes reveals the potential regulatory role of lncRNA in kiwifruit ripening and softening
Source: Sci Rep. 2021 Jan 18;11:1671. doi: 10.1038/s41598-021-81155-1 (PMC7814023; doi:10.1038/s41598-021-81155-1)
Supplement: Supplementary file 12 — Supplementary Table S10. [file 41598_2021_81155_MOESM12_ESM.doc]

**Table S10. KEGG analysis of the target genes of DELs in RT vs CK**

| **KEGG pathway** | **Map ID** | **Corrected *P* value** | **Gene number** | **Gene ID** |
| --- | --- | --- | --- | --- |
| Thiamine metabolism | ath00730 | 0.9068 | 3 | Achn259231, Achn231571, Achn259241 |
| Phagosome | ath04145 | 0.9068 | 9 | Achn207591, Achn240051, Achn094611, Achn053701, Achn370891, Achn331421, Achn234841, Achn063881, Achn095821 |
| Plant hormone signal transduction | ath04075 | 0.9068 | 21 | Achn250421, Achn215981, Achn239261, Achn355551, Achn328781, Achn025111, Achn294451, Achn181711, Achn041811, Achn265481, Achn045211, Achn078421, Achn295851, Achn363951, Achn328881, Achn156681, Achn258231, Achn278311, Achn231331, Achn089531, Achn274701 |
| Diterpenoid biosynthesis | ath00904 | 0.9068 | 3 | Achn115791, Achn111061, Achn103531 |
| Brassinosteroid biosynthesis | ath00905 | 0.9068 | 2 | Achn390991, Achn379971 |
| Glycosphingolipid biosynthesis - globo series | ath00603 | 0.9068 | 2 | Achn249581, Achn111011 |
| Endocytosis | ath04144 | 0.9068 | 10 | Achn351021, Achn109241, Achn109261, Achn181851, Achn181841, Achn378871, Achn101141, Achn047961, Achn053711, Achn128411 |
| Plant-pathogen interaction | ath04626 | 0.9068 | 13 | Achn331451, Achn234881, Achn217421, Achn132821, Achn095791, Achn132381, Achn250381, Achn371121, Achn132801, Achn055971, Achn213151, Achn132841, Achn095991 |
| Citrate cycle (TCA cycle) | ath00020 | 0.9068 | 6 | Achn150121, Achn379981, Achn103581, Achn041831, Achn168921, Achn030661 |
| Folate biosynthesis | ath00790 | 0.9068 | 3 | Achn136091, Achn150111, Achn250331 |
| Glutathione metabolism | ath00480 | 0.9068 | 8 | Achn207061, Achn265461, Achn355881, Achn355861, Achn355851, Achn255471, Achn321141, Achn259181 |
| Inositol phosphate metabolism | ath00562 | 0.9068 | 6 | Achn363941, Achn213261, Achn053701, Achn264191, Achn181821, Achn150021 |
| RNA degradation | ath03018 | 0.9068 | 9 | Achn051201, Achn004861, Achn120351, Achn321131, Achn149131, Achn255441, Achn255491, Achn378591, Achn043201 |
| ABC transporters | ath02010 | 0.9068 | 3 | Achn158571, Achn158561, Achn234451 |
| Peroxisome | ath04146 | 0.9068 | 7 | Achn005191, Achn005181, Achn156611, Achn371241, Achn251091, Achn105141, Achn310611 |
| Sphingolipid metabolism | ath00600 | 0.9068 | 3 | Achn249581, Achn186271, Achn063871 |
| Galactose metabolism | ath00052 | 0.9068 | 5 | Achn319701, Achn274381, Achn319711, Achn285641, Achn249581 |
| Ascorbate and aldarate metabolism | ath00053 | 0.9068 | 4 | Achn207061, Achn265461, Achn181821, Achn132811 |
| Sulfur relay system | ath04122 | 0.9068 | 2 | Achn136091, Achn150111 |
| Phenylalanine, tyrosine and tryptophan biosynthesis | ath00400 | 0.9070 | 5 | Achn005121, Achn265761, Achn119621, Achn207581, Achn079911 |
| Porphyrin and chlorophyll metabolism | ath00860 | 0.9410 | 4 | Achn301861, Achn041311, Achn344641, Achn051211 |
| Spliceosome | ath03040 | 0.9410 | 13 | Achn329751, Achn111021, Achn109261, Achn265541, Achn020581, Achn020941, Achn379951, Achn378841, Achn020591, Achn075491, Achn318281, Achn378591, Achn255491 |
| Ubiquinone and other terpenoid-quinone biosynthesis | ath00130 | 0.9410 | 3 | Achn053691, Achn258411, Achn072941 |
| Ubiquitin mediated proteolysis | ath04120 | 0.9410 | 10 | Achn136121, Achn103541, Achn042881, Achn186251, Achn215961, Achn382991, Achn250301, Achn331521, Achn383001, Achn363831 |
| Circadian rhythm - plant | ath04712 | 0.9410 | 3 | Achn215981, Achn215231, Achn181861 |
| Carbon fixation in photosynthetic organisms | ath00710 | 0.9410 | 5 | Achn310551, Achn264191, Achn035601, Achn185421, Achn041831 |
| Glycine, serine and threonine metabolism | ath00260 | 0.9410 | 5 | Achn384181, Achn020931, Achn103581, Achn231361, Achn079911 |
| Pentose phosphate pathway | ath00030 | 0.9410 | 4 | Achn321141, Achn035601, Achn087691, Achn285641 |
| Homologous recombination | ath03440 | 0.9410 | 4 | Achn047971, Achn004871, Achn079891, Achn231341 |
| Tyrosine metabolism | ath00350 | 0.9410 | 3 | Achn386341, Achn314981, Achn020931 |
| Non-homologous end-joining | ath03450 | 0.9410 | 1 | Achn216001 |
| Arginine and proline metabolism | ath00330 | 0.9410 | 5 | Achn336001, Achn185381, Achn105111, Achn321081, Achn305021 |
| Glycosylphosphatidylinositol(GPI)-anchor biosynthesis | ath00563 | 0.9410 | 2 | Achn224321, Achn309491 |
| Biosynthesis of amino acids | ath01230 | 0.9410 | 15 | Achn005121, Achn265761, Achn150121, Achn079911, Achn341461, Achn264191, Achn320231, Achn231361, Achn384181, Achn035601, Achn305021, Achn311951, Achn207581, Achn119621, Achn030661 |
| Glycolysis / Gluconeogenesis | ath00010 | 0.9410 | 7 | Achn240111, Achn274381, Achn103581, Achn264191, Achn285641, Achn087691, Achn041831 |
| Riboflavin metabolism | ath00740 | 0.9410 | 1 | Achn048231 |
| RNA transport | ath03013 | 0.9410 | 10 | Achn115841, Achn326291, Achn020941, Achn216021, Achn386291, Achn341291, Achn095961, Achn158551, Achn114641, Achn363831 |
| Starch and sucrose metabolism | ath00500 | 0.9410 | 11 | Achn274381, Achn319081, Achn020571, Achn319071, Achn319051, Achn285641, Achn319701, Achn319691, Achn087691, Achn319711, Achn318231 |
| Carbon metabolism | ath01200 | 0.9410 | 14 | Achn240111, Achn264191, Achn150121, Achn379981, Achn103581, Achn274381, Achn185421, Achn231361, Achn035601, Achn087691, Achn041831, Achn321141, Achn310551, Achn030661 |
| N-Glycan biosynthesis | ath00510 | 0.9410 | 3 | Achn390101, Achn099321, Achn258221 |
| C5-Branched dibasic acid metabolism | ath00660 | 0.9410 | 1 | Achn320231 |
| Amino sugar and nucleotide sugar metabolism | ath00520 | 0.9410 | 7 | Achn274381, Achn340321, Achn285641, Achn030701, Achn047511, Achn087691, Achn030671 |
| Protein processing in endoplasmic reticulum | ath04141 | 0.9410 | 12 | Achn355591, Achn109261, Achn255741, Achn383281, Achn095791, Achn309411, Achn319681, Achn250301, Achn172581, Achn132801, Achn158551, Achn331521 |
| Fructose and mannose metabolism | ath00051 | 0.9410 | 3 | Achn158041, Achn264191, Achn274381 |
| alpha-Linolenic acid metabolism | ath00592 | 0.9410 | 2 | Achn386861, Achn225261 |
| Nicotinate and nicotinamide metabolism | ath00760 | 0.9410 | 1 | Achn114951 |
| Protein export | ath03060 | 0.9410 | 3 | Achn172251, Achn169301, Achn309481 |
| Metabolic pathways | ath01100 | 0.9410 | 98 | Achn305021, Achn319021, Achn114601, Achn157981, Achn136091, Achn185381, Achn119621, Achn264191, Achn320231, Achn250331, Achn158041, Achn379971, Achn318231, Achn314981, Achn020571, Achn053691, Achn336001, Achn185421, Achn048231, Achn105111, Achn258411, Achn341461, Achn111011, Achn239241, Achn231571, Achn109251, Achn316821, Achn103581, Achn157921, Achn319701, Achn132811, Achn077761, Achn150021, Achn239251, Achn150121, Achn051211, Achn020931, Achn319071, Achn231361, Achn150111, Achn390991, Achn274381, Achn321081, Achn319081, Achn079911, Achn309471, Achn332811, Achn319711, Achn390101, Achn005121, Achn186271, Achn309491, Achn371241, Achn285641, Achn041831, Achn115861, Achn168921, Achn099321, Achn265421, Achn345601, Achn235541, Achn386861, Achn259191, Achn114951, Achn181741, Achn240111, Achn213261, Achn063871, Achn319051, Achn332801, Achn311951, Achn320151, Achn258221, Achn379981, Achn207581, Achn310551, Achn321141, Achn386341, Achn243651, Achn053701, Achn035601, Achn344641, Achn061851, Achn265761, Achn259231, Achn319691, Achn094631, Achn318621, Achn243641, Achn079891, Achn384181, Achn225261, Achn041311, Achn072941, Achn331421, Achn087691, Achn259241, Achn030661 |
| 2-Oxocarboxylic acid metabolism | ath01210 | 0.9410 | 4 | Achn305021, Achn150121, Achn320231, Achn030661 |
| Biosynthesis of secondary metabolites | ath01110 | 0.9410 | 52 | Achn115791, Achn005121, Achn265761, Achn341461, Achn340321, Achn332811, Achn239241, Achn285641, Achn035601, Achn041831, Achn344641, Achn168921, Achn310611, Achn109251, Achn316821, Achn103581, Achn061851, Achn265421, Achn264191, Achn320231, Achn030701, Achn379971, Achn030671, Achn114951, Achn240111, Achn239251, Achn150121, Achn051211, Achn020931, Achn384181, Achn231361, Achn207581, Achn332801, Achn311951, Achn390991, Achn119621, Achn235541, Achn072941, Achn041311, Achn274381, Achn379981, Achn020571, Achn053691, Achn079911, Achn047511, Achn157981, Achn087691, Achn321141, Achn305021, Achn386871, Achn258411, Achn030661 |
| Basal transcription factors | ath03022 | 0.9410 | 3 | Achn115031, Achn355581, Achn104881 |
| Lysine biosynthesis | ath00300 | 0.9410 | 1 | Achn341461 |
| Purine metabolism | ath00230 | 0.9410 | 8 | Achn265421, Achn114601, Achn079891, Achn157921, Achn285641, Achn309471, Achn320151, Achn114951 |
| Arachidonic acid metabolism | ath00590 | 0.9410 | 1 | Achn094631 |
| Cysteine and methionine metabolism | ath00270 | 0.9410 | 5 | Achn311951, Achn336001, Achn185381, Achn345601, Achn321081 |
| Ribosome biogenesis in eukaryotes | ath03008 | 0.9410 | 5 | Achn115841, Achn363811, Achn172231, Achn294411, Achn096321 |
| Pentose and glucuronate interconversions | ath00040 | 0.9410 | 4 | Achn158041, Achn319071, Achn319051, Achn319081 |
| Fatty acid degradation | ath00071 | 0.9410 | 2 | Achn386861, Achn371241 |
| Pyruvate metabolism | ath00620 | 0.9410 | 4 | Achn240111, Achn185421, Achn103581, Achn041831 |
| Glyoxylate and dicarboxylate metabolism | ath00630 | 0.9410 | 3 | Achn150121, Achn310611, Achn231361 |
| Limonene and pinene degradation | ath00903 | 0.9410 | 3 | Achn332811, Achn332801, Achn316821 |
| One carbon pool by folate | ath00670 | 0.9410 | 1 | Achn231361 |
| Stilbenoid, diarylheptanoid and gingerol biosynthesis | ath00945 | 0.9410 | 3 | Achn332811, Achn332801, Achn316821 |
| Base excision repair | ath03410 | 0.9410 | 2 | Achn079891, Achn216001 |
| Phosphatidylinositol signaling system | ath04070 | 0.9410 | 3 | Achn053701, Achn213261, Achn363941 |
| RNA polymerase | ath03020 | 0.9410 | 2 | Achn114601, Achn157921 |
| Propanoate metabolism | ath00640 | 0.9410 | 1 | Achn240111 |
| Isoquinoline alkaloid biosynthesis | ath00950 | 0.9410 | 1 | Achn020931 |
| Valine, leucine and isoleucine biosynthesis | ath00290 | 0.9410 | 1 | Achn320231 |
| Phenylalanine metabolism | ath00360 | 0.9410 | 5 | Achn109251, Achn157981, Achn258411, Achn235541, Achn020931 |
| Pyrimidine metabolism | ath00240 | 0.9410 | 5 | Achn114601, Achn079891, Achn319021, Achn114951, Achn157921 |
| Fatty acid metabolism | ath01212 | 0.9410 | 3 | Achn386861, Achn371241, Achn310151 |
| DNA replication | ath03030 | 0.9410 | 2 | Achn079891, Achn216001 |
| Ether lipid metabolism | ath00565 | 0.9410 | 1 | Achn150021 |
| Oxidative phosphorylation | ath00190 | 0.9410 | 7 | Achn243641, Achn379981, Achn243651, Achn331421, Achn318621, Achn115861, Achn181741 |
| Pantothenate and CoA biosynthesis | ath00770 | 0.9581 | 1 | Achn259191 |
| Carotenoid biosynthesis | ath00906 | 0.9595 | 1 | Achn061851 |
| Fatty acid elongation | ath00062 | 0.9725 | 1 | Achn386871 |
| Phenylpropanoid biosynthesis | ath00940 | 0.9755 | 6 | Achn109251, Achn239251, Achn239241, Achn157981, Achn258411, Achn235541 |
| mRNA surveillance pathway | ath03015 | 0.9755 | 4 | Achn020941, Achn194691, Achn384151, Achn332851 |
| Biosynthesis of unsaturated fatty acids | ath01040 | 0.9755 | 1 | Achn310151 |
| Tropane, piperidine and pyridine alkaloid biosynthesis | ath00960 | 0.9755 | 1 | Achn020931 |
| Fatty acid biosynthesis | ath00061 | 0.9767 | 1 | Achn371241 |
| beta-Alanine metabolism | ath00410 | 0.9767 | 1 | Achn020931 |
| Ribosome | ath03010 | 0.9767 | 14 | Achn169331, Achn163581, Achn381821, Achn063751, Achn234421, Achn063761, Achn234401, Achn301831, Achn102721, Achn383031, Achn119981, Achn311941, Achn119991, Achn320071 |
| Valine, leucine and isoleucine degradation | ath00280 | 0.9767 | 1 | Achn103581 |
| Tryptophan metabolism | ath00380 | 0.9767 | 1 | Achn310611 |
| SNARE interactions in vesicular transport | ath04130 | 0.9767 | 1 | Achn240051 |
| Glycerophospholipid metabolism | ath00564 | 0.9767 | 2 | Achn150021, Achn225261 |
| Glycerolipid metabolism | ath00561 | 0.9767 | 1 | Achn249581 |
| Cyanoamino acid metabolism | ath00460 | 0.9887 | 1 | Achn231361 |
| Nucleotide excision repair | ath03420 | 0.9929 | 1 | Achn079891 |
| Photosynthesis | ath00195 | 0.9929 | 1 | Achn077761 |
| Aminoacyl-tRNA biosynthesis | ath00970 | 0.9976 | 1 | Achn271321 |
