# Supplementary material for: Integrated analysis of lncRNA and mRNA transcriptomes reveals the potential regulatory role of lncRNA in kiwifruit ripening and softening
Source: Sci Rep. 2021 Jan 18;11:1671. doi: 10.1038/s41598-021-81155-1 (PMC7814023; doi:10.1038/s41598-021-81155-1)
Supplement: Supplementary file 13 — Supplementary Table S11. [file 41598_2021_81155_MOESM13_ESM.doc]

**Table S11. Common DELs between ABA vs CK and RT vs CK and their predicted target genes**

| **Transcript ID** | **Gene ID** | **log_2_FPKM (ABAvsCK)** | **log_2_FPKM (RTvsCK)** | **Target Gene ID** | **log_2_FPKM (ABAvsCK)** | **log_2_FPKM (RTvsCK)** | **Gene Description** |
| --- | --- | --- | --- | --- | --- | --- | --- |
| TCONS_00077491 | XLOC_035972 | 3.32 | -6.59 | Achn301791 | 0.8 | 1.37 | Uncharacterized protein |
|  |  |  |  | Achn301801 | 0 | 0 | Unknown |
|  |  |  |  | Achn301811 | 0.52 | -0.5 | TBC1 domain family member 13 |
|  |  |  |  | Achn301821 | 0 | 0 | Uncharacterized protein |
|  |  |  |  | Achn301831 | -0.63 | 0.36 | Hypothetical protein |
|  |  |  |  | Achn301841 | 1.15 | 1.62 | Hypothetical protein |
|  |  |  |  | Achn301851 | 8.96 | 0 | SPX domain-containing protein 1 |
|  |  |  |  | Achn301861 | 2.17 | 2.13 | Unnamed protein product |
|  |  |  |  | Achn301871 | -2.69 | -0.09 | Uncharacterized protein |
|  |  |  |  | Achn383271 | 0.66 | 0.06 | Uncharacterized protein |
|  |  |  |  | Achn383281 | -1.17 | -0.46 | 17.6 kDa class II heat shock protein |
|  |  |  |  | Achn383291 | 0 | 0 | Unknown |
|  |  |  |  | Achn383301 | 1.05 | 1.64 | Nuclear pore complex protein NUP1 isoform X1 |
|  |  |  |  | Achn383311 | 0.5 | -1.12 | Methyl-CPG-binding domain 10 |
|  |  |  |  | Achn383321 | 0.96 | 0.9 | Beta-1,3-glucanase 1 |
|  |  |  |  | Achn383951 | 0 | 0 | Unknown |
|  |  |  |  | Achn383961 | 0 | 0 | Unknown |
|  |  |  |  | Achn383971 | 0 | 0 | Thylakoid lumenal 16.5 kDa protein |
|  |  |  |  | Achn383981 | -1.08 | -3.07 | Axial regulator YABBY 5-like |
|  |  |  |  | Achn383991 | -0.88 | -0.44 | CBL-interacting serine/threonine-protein kinase 24 |
|  |  |  |  | Achn384001 | 1.29 | 2.19 | Transmembrane protein 230 |
| TCONS_00094824 | XLOC_042139 | 3.98 | 5.18 | Achn349241 | -0.05 | 1.03 | Pentatricopeptide repeat-containing protein |
|  |  |  |  | Achn349251 | 1.5 | 0.62 | E3 ubiquitin-protein ligase RGLG2-like isoform X2 |
|  |  |  |  | Achn349261 | 0 | 0 | Vegetative incompatibility protein HET-E-1 |
|  |  |  |  | Achn350991 | 0.66 | -0.96 | Unnamed protein product |
|  |  |  |  | Achn351001 | 0.44 | 0.76 | Protein-tyrosine-phosphatase MKP1-like |
|  |  |  |  | Achn390061 | 0 | 0 | TRANSPARENT TESTA 12-like |
|  |  |  |  | Achn390071 | 0.7 | 0.22 | Hypothetical protein |
|  |  |  |  | Achn390081 | 0 | 0 | Ethylene-responsive transcription factor ESR2-like |
|  |  |  |  | Achn390091 | -1.75 | 1.81 | Uncharacterized protein |
|  |  |  |  | Achn390101 | 1.27 | 0.39 | Uncharacterized protein |
|  |  |  |  | Achn390111 | -1.25 | 0.13 | Uncharacterized protein |
|  |  |  |  | Achn390121 | -0.28 | -0.8 | Uncharacterized protein |
|  |  |  |  | Achn390131 | -0.05 | 0.12 | Coilin isoform X3 |
|  |  |  |  | Achn390141 | 0.21 | 0.61 | Protein-tyrosine-phosphatase MKP1-like |
|  |  |  |  | Achn390151 | 0.58 | 1.68 | Unnamed protein product |
|  |  |  |  | Achn390161 | 1.06 | 0.3 | Chloroplast sensor kinase |
|  |  |  |  | Achn390171 | -0.52 | -1.34 | Unnamed protein product |
|  |  |  |  | Achn390181 | 0.54 | 2.18 | Uncharacterized protein |
| TCONS_00330037 | XLOC_140553 | 4.55 | 4.25 | Achn102661 | 0.03 | 0.05 | Uncharacterized protein |
|  |  |  |  | Achn102671 | 2.65 | -0.2 | Hypothetical protein |
|  |  |  |  | Achn102681 | 0 | 0 | Unknown |
|  |  |  |  | Achn102691 | 0 | 0 | Pectinesterase-like |
|  |  |  |  | Achn102701 | 0.5 | 0.7 | Craniofacial development protein 1 |
|  |  |  |  | Achn102711 | 3.18 | 3.22 | Pectinesterase inhibitor |
|  |  |  |  | Achn102721 | 0.48 | 0.77 | 30S ribosomal protein S1 |
|  |  |  |  | Achn102731 | 0 | 0 | R2R3 transcription factor MYB108-like protein 1 |
|  |  |  |  | Achn102741 | 0 | 0 | Unknown |
|  |  |  |  | Achn103531 | 0 | 0 | Gibberellin 2-beta-dioxygenase 2 |
|  |  |  |  | Achn103541 | 0.31 | -0.14 | E3 ubiquitin-protein ligase UPL6 |
|  |  |  |  | Achn103551 | 0 | inf | Serine carboxypeptidase S10 family protein |
|  |  |  |  | Achn103561 | -0.15 | -1.74 | Probable protein Pop3 |
|  |  |  |  | Achn103571 | 0.66 | -0.71 | Hypothetical protein |
|  |  |  |  | Achn103581 | 0.86 | -1.05 | Putative branched-chain alpha-keto acid dehydrogenase E3 subunit |
|  |  |  |  | Achn103591 | 0 | 0 | Hypothetical protein |
| TCONS_00376254 | XLOC_155131 | -8.16 | -6.45 | Achn213141 | 0 | 0 | Unknown |
|  |  |  |  | Achn213151 | 3.5 | -0.13 | Unnamed protein product |
|  |  |  |  | Achn213161 | 0.84 | 3.04 | Unnamed protein product |
|  |  |  |  | Achn213171 | 0 | 0 | Hypothetical protein |
|  |  |  |  | Achn213181 | 0.18 | 2.02 | Hypothetical protein |
|  |  |  |  | Achn213191 | 0 | 0 | LEAFY-like protein |
|  |  |  |  | Achn213201 | -0.03 | -1.44 | Pentatricopeptide repeat-containing protein |
|  |  |  |  | Achn213211 | -0.46 | 2.33 | Catalytic, putative |
|  |  |  |  | Achn213221 | -4.14 | -3.39 | Rub |
|  |  |  |  | Achn213231 | -0.69 | 2.64 | Uncharacterized protein |
|  |  |  |  | Achn213241 | 1.54 | 0.14 | Calcium-binding mitochondrial carrier protein SCaMC-2-B-like |
|  |  |  |  | Achn213251 | 0.3 | 0.03 | Unnamed protein product |
|  |  |  |  | Achn213261 | 0 | 0 | Inositol polyphosphate multikinase beta |
|  |  |  |  | Achn213271 | #NAME? | #NAME? | Uncharacterized protein |
|  |  |  |  | Achn213281 | 0.41 | -0.02 | Uncharacterized protein |
|  |  |  |  | Achn271041 | 0 | 0 | Uncharacterized protein |
|  |  |  |  | Achn271321 | -0.09 | 1.05 | Glycine--tRNA ligase 1 |
| TCONS_00611883 | XLOC_255841 | -7.31 | -7.16 | Achn106211 | 0 | 0 | Unknown |
|  |  |  |  | Achn106221 | 0.71 | 0.16 | Shugoshin-1-like isoform X2 |
|  |  |  |  | Achn225101 | 0 | 0 | Unknown |
|  |  |  |  | Achn225111 | #NAME? | #NAME? | Squamosa promoter-binding-like protein 7 |
|  |  |  |  | Achn225601 | -1.07 | -0.94 | Squamosa promoter-binding-like protein 8 |
|  |  |  |  | Achn225611 | 0 | 0 | Metalloendoproteinase 1 precursor |
|  |  |  |  | Achn225621 | 0 | 0 | T-complex protein 1 subunit alpha |
|  |  |  |  | Achn225631 | 0 | 0 | GATA transcription factor 4 |
| TCONS_00696001 | XLOC_270586 | 3.9 | -5.68 | Achn264141 | 1.2 | 2.23 | F-box and wd40 domain protein |
|  |  |  |  | Achn264151 | 0 | 0 | Unknown |
|  |  |  |  | Achn264161 | -1.31 | 0.2 | Uncharacterized protein C20orf24 homolog |
|  |  |  |  | Achn264171 | 1.89 | 1.69 | Uncharacterized protein |
|  |  |  |  | Achn264181 | 1.04 | 2 | Probable receptor-like protein kinase |
|  |  |  |  | Achn264191 | -1.69 | -0.6 | Hypothetical protein |
|  |  |  |  | Achn264201 | -0.05 | 0.83 | Protein kinase APK1B |
|  |  |  |  | Achn264211 | 0 | 0 | Unknown |
|  |  |  |  | Achn264221 | inf | 0 | Hypothetical protein |
|  |  |  |  | Achn264231 | 0.48 | -0.07 | Oxysterol-binding protein-related protein 3C |
|  |  |  |  | Achn264241 | 2.04 | 1.47 | Unnamed protein product |
|  |  |  |  | Achn264251 | 1.59 | -0.31 | Scarecrow-like protein 14 |
| TCONS_00781977 | XLOC_294112 | -3.94 | -3.87 | Achn387231 | 0.76 | 0.15 | Exocyst complex component SEC5A |
|  |  |  |  | Achn387241 | 2.33 | 3.17 | Sequence-specific DNA binding transcription factors |
|  |  |  |  | Achn387251 | -0.1 | -1.37 | Hypothetical protein |
|  |  |  |  | Achn387261 | 0.18 | 0.88 | Tubby-like F-box protein 8 |
|  |  |  |  | Achn387271 | inf | 0 | ABC transporter F family member 4 |
|  |  |  |  | Achn387281 | -0.08 | 0.96 | Sterol 3-beta-glucosyltransferase UGT80B1 isoform X1 |
|  |  |  |  | Achn387291 | -0.63 | 0.16 | Hypothetical protein |
|  |  |  |  | Achn387301 | 3.64 | -0.22 | Abscisic stress-ripening protein 2-like |
|  |  |  |  | Achn387311 | 3.2 | 0.05 | Hypothetical protein |
|  |  |  |  | Achn387321 | inf | 0 | Uncharacterized protein |
|  |  |  |  | Achn387331 | 4.17 | 4.09 | ASR3 |
|  |  |  |  | Achn387341 | 4.61 | 4.07 | ASR3 |
|  |  |  |  | Achn387351 | inf | 0 | Abscisic stress-ripening protein 2-like |
|  |  |  |  | Achn387361 | -0.88 | 1 | ASR3 |
|  |  |  |  | Achn388401 | 0.54 | 0.69 | Unnamed protein product |
|  |  |  |  | Achn388411 | 0 | 0 | Uncharacterized protein |
|  |  |  |  | Achn388421 | 0 | 0 | WAT1-related protein |
