# Supplementary material for: Integrated analysis of lncRNA and mRNA transcriptomes reveals the potential regulatory role of lncRNA in kiwifruit ripening and softening
Source: Sci Rep. 2021 Jan 18;11:1671. doi: 10.1038/s41598-021-81155-1 (PMC7814023; doi:10.1038/s41598-021-81155-1)
Supplement: Supplementary file 14 — Supplementary Table S12. [file 41598_2021_81155_MOESM14_ESM.doc]

**Table S12. Oligonucleotide primers used for real-time quantitative PCR analysis**

| **Gene ID** | **Forward primer (5′-3′)** | **Reverse primer (5′-3′)** |
| --- | --- | --- |
| Achn024671 | CGCCGGATTCTCTTCCTAATAC | TCGTCGAAGCTCCAAAGTTC |
| Achn132211 | CAGCTCCAACCTTCAACATTTC | CCTGAGAGTGCCACTAAGTCATC |
| Achn187281 | AGTGCTACATGGCTTCACTCC | CAGCCGCCACTTTCTTCTC |
| Achn042871 | CGGAATTCGGCTGCTACTTA | GCTCAAACCTACGCCTTCTTC |
| Achn111051 | TTGGCGAGCAAGAGTGGTA | CTTATCGGTCCCAGTAGCTTTC |
| Achn379971 | GGCACGACGATGAGCAATAC | TGAGGACTAGGTGGTGGATGAA |
| Achn294421 | ATTCACCAAAGACCCGTAGAAG | CCTAATCAAGCCGACCATGTAG |
| Achn235381 | CACTGCAAATGCTCCAAAG | CACTGCAAATGCTCCAAAG |
| Achn107181 | GTGCTCAGTGGTGGTTCAA | GACGCTGTATTTCCTCTCAG |
| TCONS_00299670 | CGGAACCAGTGTATTGCTCATA | TGTTCCTCTGCTGTTGCCTAG |
| TCONS_00301130 | GGAAACTTTCGCAGGAATCA | GGGACTCTTCGTTCTTCTTGTC |
| TCONS_00045079 | TGAGATACGCTGACCCAAATC | CACATTTGCTGCGTTCTTGTA |
| TCONS_00518923 | TCGCAACCACAGACTCAAACTA | GGTCTTGACTGGATGAGAATGTC |
| TCONS_00293626 | CCTTCTTTGGCAGCATGAAAG | GACGAAGTTACCCAGAAGATCG |
| TCONS_00619335 | TCGCCTGTATCGTCACTAGAG | ACCACCAACCATGAGCATTA |
| TCONS_00613757 | CGTGAGTCTAGAGGCGATTTAAC | GGAGATAATACAAGTGCAGGTACA |
| TCONS_00635380 | TGGGTATTAGGAGGAGGATGAG | CAGTAGCCGAGTCTGGTCCTTA |
| TCONS_00611457 | GCAGATCAGCGCGTAGCTA | GATCACCATTCGATGAGAACC |
